# Structure and Reactivity of [Ru–Al] and [Ru–Sn] Heterobimetallic PPh_3_-Based Complexes

**DOI:** 10.1021/acs.organomet.2c00344

**Published:** 2022-09-21

**Authors:** Connie J. Isaac, Fedor M. Miloserdov, Anne-Frédérique Pécharman, John P. Lowe, Claire L. McMullin, Michael K. Whittlesey

**Affiliations:** Department of Chemistry, University of Bath, Bath BA2 7AY, U.K.

## Abstract

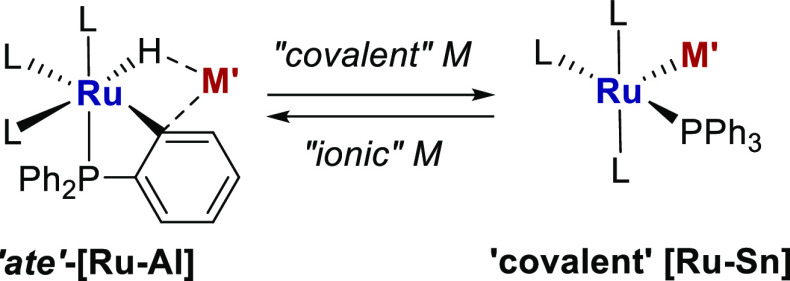

Treatment of [Ru(PPh_3_)(C_6_H_4_PPh_2_)_2_H][Li(THF)_2_] with AlMe_2_Cl and SnMe_3_Cl leads to elimination of LiCl and CH_4_ and formation of the heterobimetallic complexes [Ru(C_6_H_4_PPh_2_)_2_{PPh_2_C_6_H_4_AlMe(THF)}H] **5** and [Ru(PPh_3_)(C_6_H_4_PPh_2_)(PPh_2_C_6_H_4_SnMe_2_)] **6**, respectively. The pathways to **5** and **6** have been probed by variable temperature NMR studies, together with input from DFT calculations. Complete reaction of H_2_ occurs with **5** at 60 °C and with **6** at room temperature to yield the spectroscopically characterized trihydride complexes [Ru(PPh_2_)_2_{PPh_2_C_6_H_4_AlMe}H_3_] **7** and [Ru(PPh_2_)_2_{PPh_2_C_6_H_4_SnMe_2_}H_3_] **8**. In the presence of CO, **6** forms the acylated phosphine complex, [Ru(CO)_2_(C(O)C_6_H_4_PPh_2_)(PPh_2_C_6_H_4_SnMe_2_)] **9**, through a series of intermediates that were identified by NMR spectroscopy in conjunction with ^13^CO labeling. Complex **6** undergoes addition and substitution reactions with the N-heterocyclic carbene 1,3,4,5-tetramethylimidazol-2-ylidene (IMe_4_) to give [Ru(IMe_4_)_2_(PPh_2_C_6_H_4_)(PPh_2_C_6_H_4_SnMe_2_)] **10**, which converted via rare N-Me group C–H activation to [Ru(IMe_4_)(PPh_3_)(IMe_4_)′(PPh_2_C_6_H_4_SnMe_2_)] **11** upon heating at 60 °C and to a mixture of [Ru(IMe_4_)_2_(IMe_4_)′(PPh_2_C_6_H_4_SnMe_2_)] **12** and [Ru(PPh_3_)(PPh_2_C_6_H_4_)(IMe_4_-SnMe_2_)′] **13** at 120 °C.

## Introduction

**Scheme 1:**
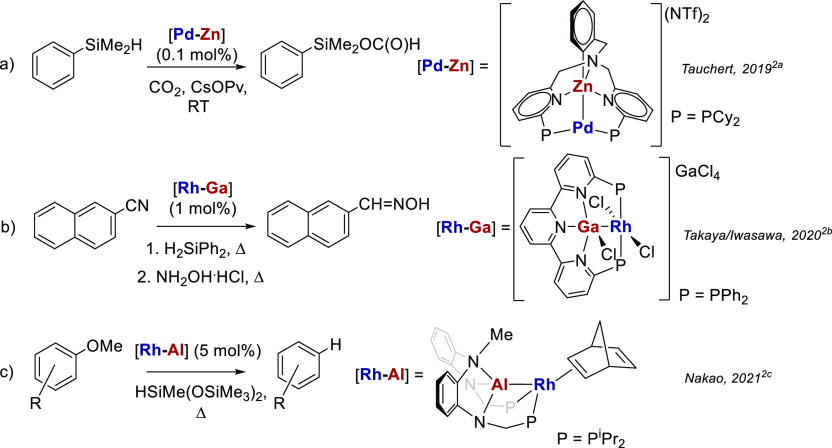
Examples of Catalytic Transformations Mediated by [TM-M′] Heterobimetallic Complexes

Heterobimetallic (HBM) complexes featuring a transition metal (TM) center in combination with a Lewis acidic *s*- or *p*-block metal (M′) continue to be the subject of considerable interest, primarily due to the ability of such species to bring about small molecule activation chemistry.^1,2^ The TM-Zn, -Ga and -Al complexes shown in Scheme 1 represent three recent examples in which [TM-M′] HBM complexes have been employed to bring about not only small molecule activation but also a subsequent catalytic functionalization step.

**Scheme 2:**
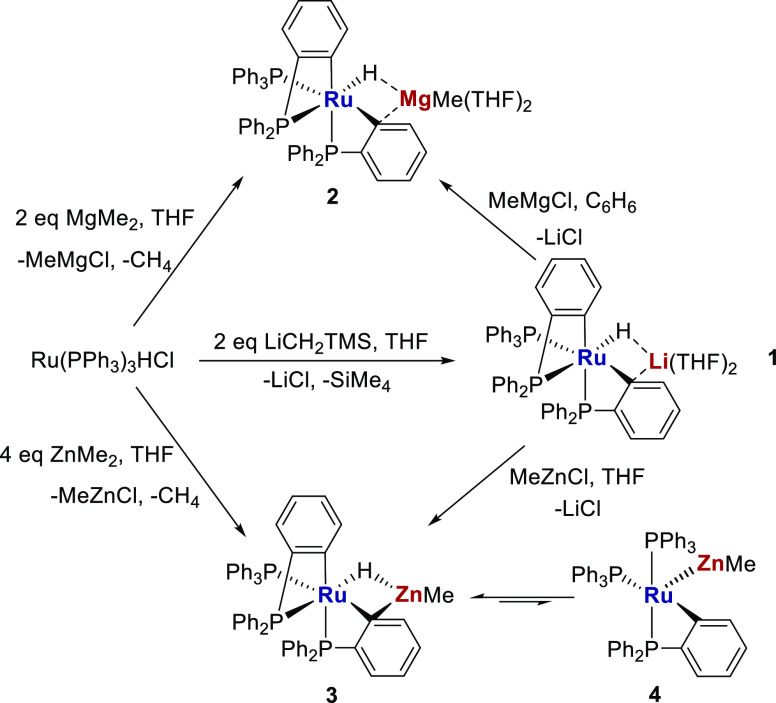
Synthesis of [Ru-M′] Complexes **1** (M′ = Li(THF)_2_), **2** (M′ = MgMe(THF)_2_) and **3** (M′ = ZnMe) and Equilibrium of the Latter with **4**

A commonly employed preparative route to [TM-M′] HBM complexes involves the reaction of a TM-hydride precursor with a Lewis acidic metal alkyl reagent to give a [TM-M′] product following elimination of an alkane.^3^ In a recent study,^4^ we employed such a reaction of [Ru(PPh_3_)_3_HCl] with LiMe, MgMe_2_, and ZnMe_2_ to give the bis-cyclometalated complexes^5^ [Ru(PPh_3_)(C_6_H_4_PPh_2_)_2_H][M′] (M′ = Li(THF)_2_**1**, MgMe(THF)_2_**2**, and ZnMe **3**) shown in Scheme 2. A combination of X-ray crystallography and DFT calculations showed that the level of interaction between Ru–H and M′ increased in the order of **1** < **2** < **3** such that **1** and **2** were best considered as ruthenate anions with a group 1 or 2 countercation, whereas Zn compound **3** exhibited far more covalent character. As a result, the latter proved susceptible to reductive elimination of the hydride ligand onto one of the metalated phosphines, to yield the “dual unsaturated” isomer, [Ru(PPh_3_)_2_(C_6_H_4_PPh_2_)(ZnMe)] **4**, which although only present in ca. 2%, allowed **3** to react with H_2_ at −40 °C, ca. 100 °C lower than the temperature required with either **1** and **2**.

Prompted by the enhanced reactivity of the [Ru–Zn] complex, we have extended our studies to [Ru-M′] complexes in which M′ = Al and Sn, on the basis that they would also exhibit strong covalent interactions with the Ru center. We now describe the synthesis and reactivity of the [Ru–Al] and [Ru–Sn] heterobimetallic complexes [Ru(C_6_H_4_PPh_2_)_2_{PPh_2_C_6_H_4_AlMe(THF)}H] **5** and [Ru(PPh_3_)(C_6_H_4_PPh_2_)(PPh_2_C_6_H_4_SnMe_2_)] **6**.

## Results and Discussion

### Synthesis and Characterization of [Ru(C_6_H_4_PPh_2_)_2_{PPh_2_C_6_H_4_AlMe(THF)}H] and [Ru(PPh_3_)(C_6_H_4_PPh_2_)(PPh_2_C_6_H_4_SnMe_2_)]

**Scheme 3:**
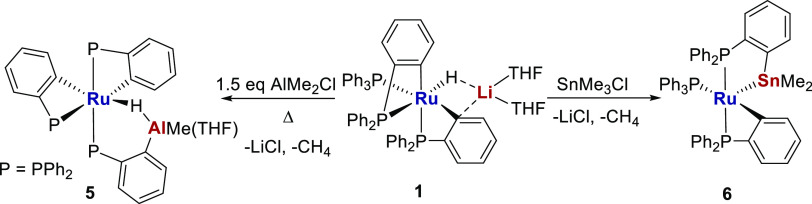
Synthesis of [Ru(C_6_H_4_PPh_2_)_2_{PPh_2_C_6_H_4_AlMe(THF)}H] **5** and [Ru(PPh_3_)(C_6_H_4_PPh_2_)(PPh_2_C_6_H_4_SnMe_2_)] **6**

We showed previously that the [Ru–Li] salt **1** was a convenient precursor to both **2** and **3** upon treatment with MgMeCl and ZnMeCl, respectively (Scheme 2), thanks to the relative ease of removal of the LiCl byproduct.^4^ Heating **1** with AlMe_2_Cl at 60 °C led to full conversion through to yellow [Ru(C_6_H_4_PPh_2_)_2_{PPh_2_C_6_H_4_AlMe(THF)}H] **5**, which was isolated in 69% yield, whereas SnMe_3_Cl reacted with **1** at room temperature to generate deep-blue [Ru(PPh_3_)(C_6_H_4_PPh_2_)(PPh_2_C_6_H_4_SnMe_2_)] **6** in a near quantitative amount (Scheme 3). Comparison of Schemes 2 and 3 shows that while both reactions were indeed accompanied by loss of LiCl, the availability of additional M′-Me groups on moving from ZnMeCl to AlMe_2_Cl and SnMe_3_Cl allowed elimination of an extra molecule of CH_4_, resulting in metalation of a further phosphine ligand (vide infra).^6^

**Figure 1:**
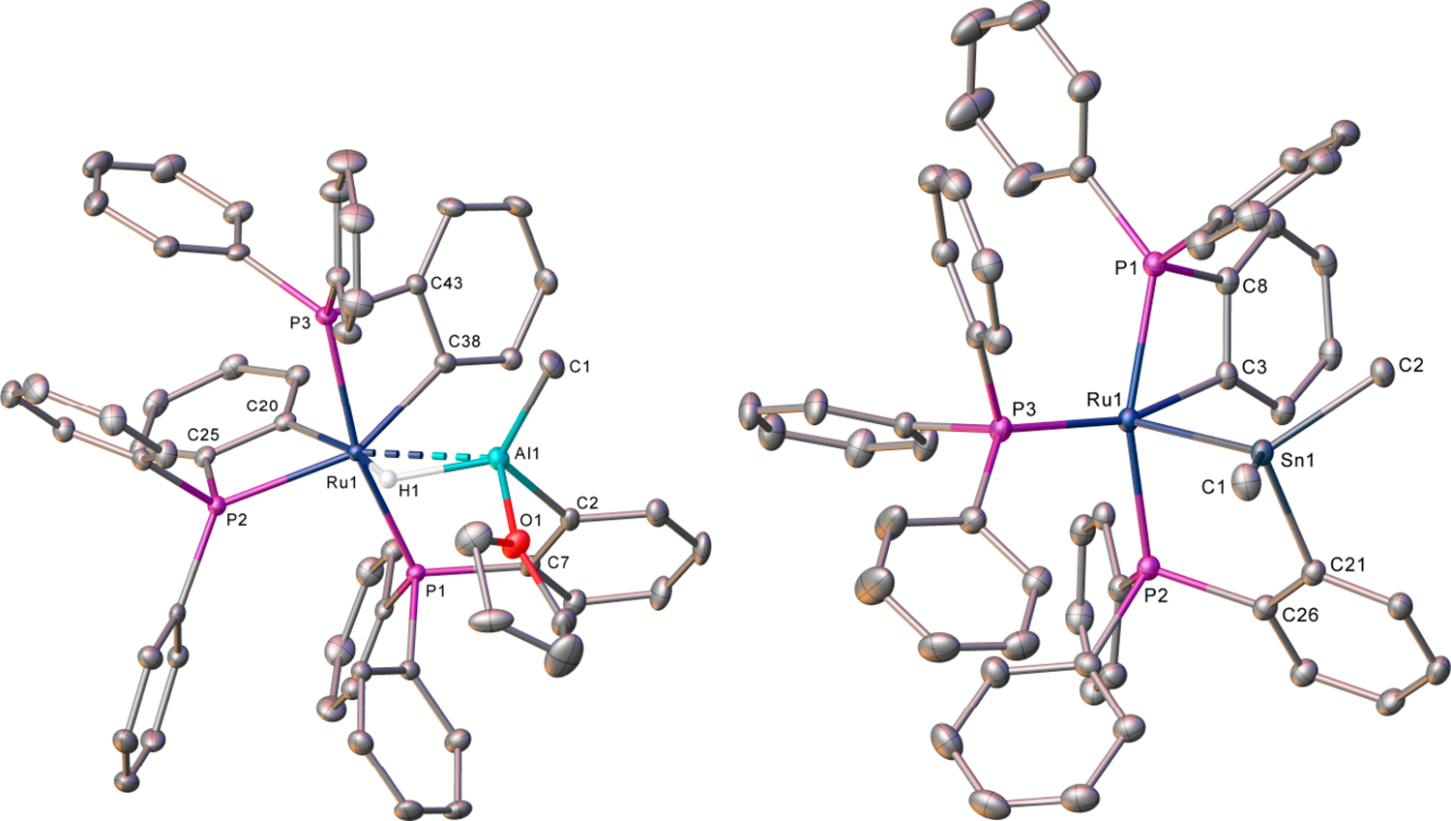
Molecular structures of (left) **5** and (right) **6**. Ellipsoids at 30% level; all H atoms, except Ru–H–Al, omitted for clarity. In **5**, the solvent and the minor disordered component have also been omitted for clarity.

The X-ray crystal structure of **5** (Figure 1, Table 1) showed a Ru atom at the center of a highly distorted octahedral arrangement of ligands (e.g., P(2)–Ru(1)–C(38) = 150.40(6)°), including three metalated phosphines^7^ in a *mer*-arrangement (cf. *fac*-RuP_3_ geometry of both **1** and **2**). The ruthenium and aluminum centers formed part of a 6-membered ring dimetalacycle in which the Al was attached to both a bridging hydride ligand (located and refined with an Al(1)–H(1) distance of 1.83(3) Å) and a phosphine phenyl group (Al(1)–C(2) = 1.991(3) Å).^8,9^ The structure of **6** (Figure 1, Table 1) contained a 5-coordinate Ru center (thus resembling **4**) with a direct Ru–Sn interaction (Ru(1)–Sn(1) = 2.5686(2) Å).^10^ Phosphine metalation occurred onto Sn to generate a cyclostannylated phosphine ligand, which bridges across the basal and axial sites of the square pyramidal Ru complex. A related, albeit coordinatively saturated, osmium derivative, [Os(PPh_3_)(CO)(C_6_H_4_PPh_2_)(PPh_2_C_6_H_4_SnMe_2_)], has been reported by Roper to form as a minor product upon refluxing [Os(PPh_3_)_2_(CO)(SnMe_3_)Cl] with PPh_3_.^11−13^

**Table 1:** Selected Bond Lengths (Å) and Angles (deg) in **5** and **6**

	**5**	**6**
Ru-PPh_3_	-	2.3504(6)
Ru-PPh_2_(C_6_H_4_*Ru*)	2.3854(6), 2.3555(6)	2.3616(7)
Ru-PPh_2_(C_6_H_4_*Al*)	2.3202(6)	-
Ru-PPh_2_(C_6_H_4_*Sn*)	-	2.3245(6)
Ru···E	(E = Al) 2.5911(7)	(E = Sn) 2.5686(2)
*trans*–P–Ru-P	166.16(2)	158.22(2)

The solution NMR spectra of **5** and **6** (Figures S1–S7) were consistent with their solid-state structures.^14^ Thus, the ^1^H NMR spectrum of **5** showed a broad triplet of doublets Ru-*H*-Al signal at δ −6.29^15^ with small ^2^*J*_HP_ splittings (12 and 6 Hz) to the three cis-phosphorus nuclei. In the ^31^P{^1^H} NMR spectrum, there were three doublets, with those at δ 70 and δ −15 assigned to the phosphines metalated onto Al and Ru respectively, based on their mutually large (trans) coupling of 266 Hz, as well as the established upfield shift associated with phosphines metalated onto a TM center and downfield shift arising from 6-membered ring phosphine chelates.^16,17^ The ^1^H NMR spectrum of **6** yielded very little in the way of diagnostic information, but the presence of high (δ 75) and low (δ −29) frequency ^31^P{^1^H} NMR signals with a large mutual ^2^*J*_PP_ splitting of 240 Hz was consistent with the presence of cyclostannylated and cycloruthenated ligands respectively.

### Pathways to Formation of **5** and **6**

**Scheme 4:**
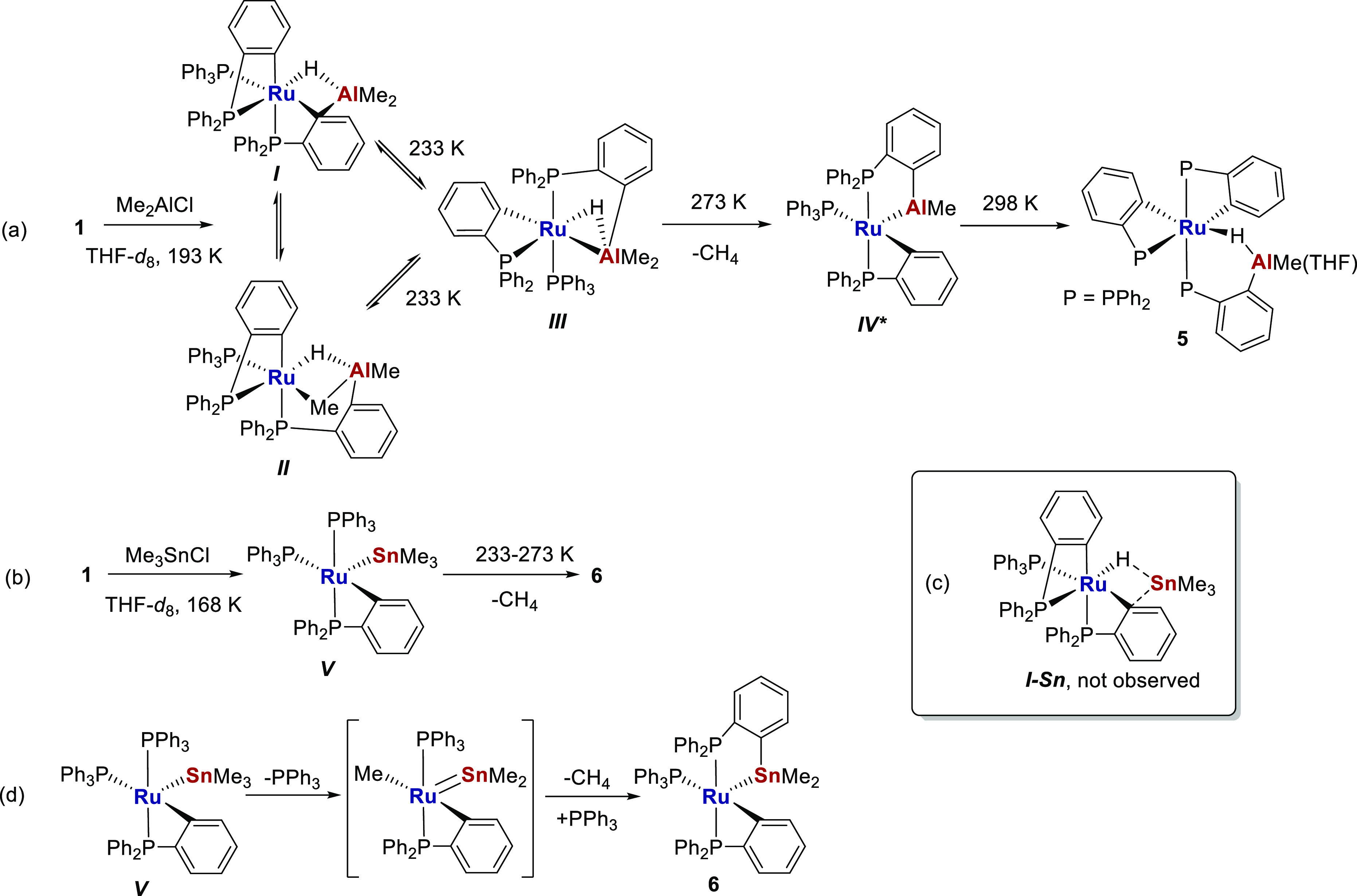
Proposed Structures of Intermediates in the Formation of **5** and **6** (Based on Low-Temperature NMR Studies) Are Shown in (a–c), with a Proposed Pathway from ***V*** to **6** Illustrated in (d) The * on ***IV*** in part (a) denotes uncertainty as to whether THF is or is not bound on Al.

The very different structures of **5** and **6** led us to investigate their pathways to formation using variable temperature NMR spectroscopy. Introduction of a frozen, yellow-orange THF-*d*_8_ solution of **1** and AlMe_2_Cl into a precooled (193 K) NMR probe revealed the rapid formation of a 1:1 ratio of two intermediates, assigned as the structures ***I*** and ***II*** shown in Scheme 4a. Characterization of these species (Figures S8–S12), as well as the higher temperature intermediates ***III*** and ***IV*** (Scheme 4a), was based on (i) the number of ^31^P NMR resonances and their relative chemical shifts,^16,18^ (ii) the relative magnitudes of ^2^*J*_PP_/^2^*J*_HP_ couplings, and (iii) ^31^P–^1^H HMQC connectivities.

Intermediate ***I*** results from substitution of the Li(THF)_2_ moiety in **1** by AlMe_2_ and was assigned based on the retention of a *fac*-RuP_3_ arrangement, comparable ^31^P chemical shifts to those of **1** (especially the two low frequency resonances for the metalated phosphines) and the presence of a low frequency hydride resonance (δ −10.50 cf. δ −9.62 in **1**),^4^ attributed to the bridging Ru-*H*-Al interaction. Intermediate ***II*** showed a broad ^1^H singlet at δ −2.53, in a 3:1 ratio with doublet of doublet of doublets Ru-*H*-Al signal at δ −13.92, suggestive of it being an isomer with a bridging Ru-*Me*-Al group in place of the Ru-*C*_*6*_*H*_*4*_-Al bridge in ***I***. The replacement of one of the low-frequency ^31^P signals in ***I*** by a new high frequency signal for ***II*** supported the presence of a phosphine metalated onto just Al.

Isomerization of ***I*** and ***II*** to the mer-***III*** was seen at 233 K, while further warming (to 273 K) generated a deeper-red colored solution, consistent with formation of a coordinatively unsaturated isomer ***IV***. This showed just a single Al*Me* proton resonance (cf. two resonances in ***III***), consistent with a structure arising out of the combination of the Ru-*H*-Al and one of the two Al*Me* groups in ***III*** and subsequent reductive elimination of methane. After 1 h at 273 K, ***I***-***III*** had been fully consumed, and ***IV*** represented ca. 80% of the reaction mixture. A final color change from red to orange was observed at 298 K, concomitant with the formation of the final product **5** through metalation of the third phosphine ligand. After ca. 40 min at 298 K, **5** comprised ca. 65% of all species in solution.^19^

An analogous study of the formation of **6** (Scheme 4b; Figures S13–S16) failed to show any spectroscopic evidence for the comparable initial substitution product ***I–Sn*** (Scheme 4c). This may imply that reductive elimination of Ru–H onto RuC_6_H_4_PPh_2_ in such a species is very fast, supporting further the analogous behavior of [Ru–Sn] and [Ru–Zn] species. Only a single (deep-blue) intermediate was observed between 168 and 273 K, which we propose is [Ru(PPh_3_)_2_(C_6_H_4_PPh_2_)SnMe_3_] (***V***) based on (i) the presence of only a single Sn*Me* resonance in the ^1^H NMR spectrum (cf. two signals in **6** for the diastereotopic Me groups) and (ii) the observation of a single low frequency (δ −29) ^31^P{^1^H} NMR signal for a cycloruthenated phosphine, together with two “medium” frequency signals (δ 49, 41–cf. ***IV***) arising from two PPh_3_ ligands. **6** began to appear above 273 K (Figures S13–S16). Following studies by Wada^20^ and Roper,^11b^ a possible pathway for the transformation of ***V*** to **6** involves Me group transfer from Sn to Ru and generation of a transient Ru stannylene intermediate, (Scheme 4d) that could generate the final cyclostannylated phosphine containing complex **6** through attack on a phosphine phenyl C–H bond, followed by elimination of methane.

**Scheme 5:**
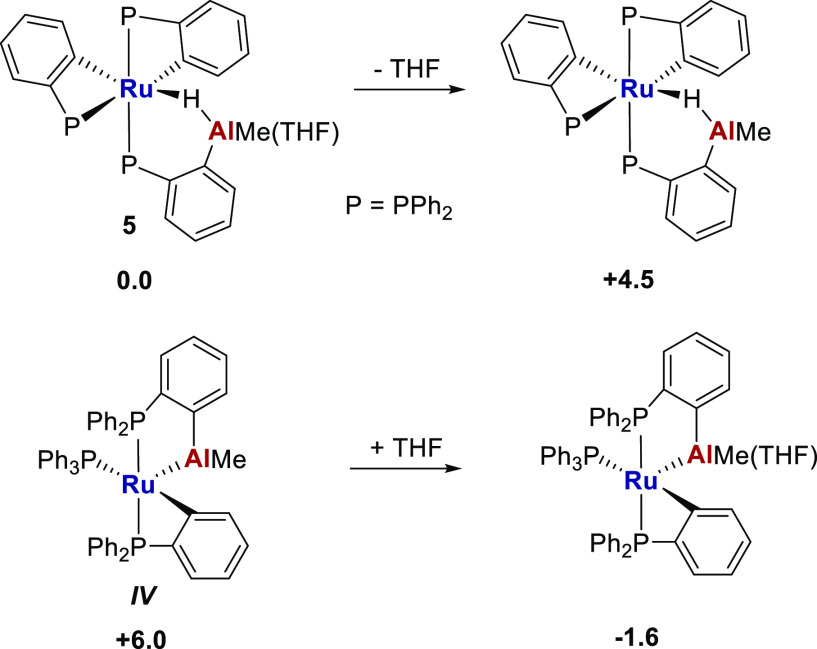
Free Energies (BP86-D3BJ(C_6_H_6_)/BS2//BP86/BS1) Relative to **5** (kcal/mol)

**Scheme 6:**
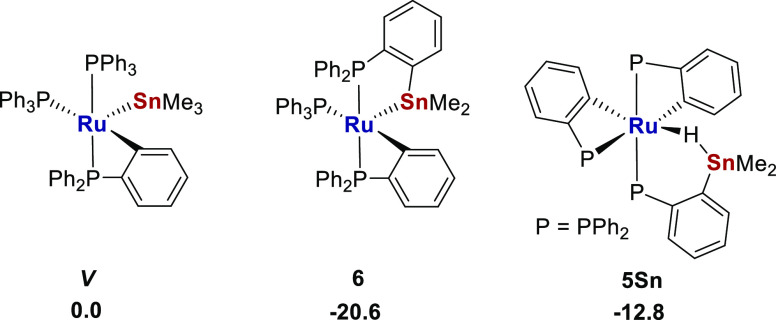
Free Energies (BP86-D3BJ(C_6_H_6_)/BS2//BP86/BS1) Relative to ***V*** (kcal/mol)

Determination of the free energies of ***IV***, ***V***, **5**, and **6** by density functional theory (DFT) calculations (BP86-D3BJ(C_6_H_6_)/BS2//BP86/BS1) were in agreement with the experimental findings. Thus, the free energy of ***IV*** was computed to be higher than that of **5** (Scheme 5; see also Supporting Information for further details). Unsurprisingly, coordination of THF stabilized both structures. **6** was calculated to be more stable than ***V***, as well as 7.8 kcal/mol more stable than **5Sn**, the Sn analogue of [Ru–Al] complex **5** (Scheme 6).

### Reactivity of **5** and **6** with H_2_

**Scheme 7:**
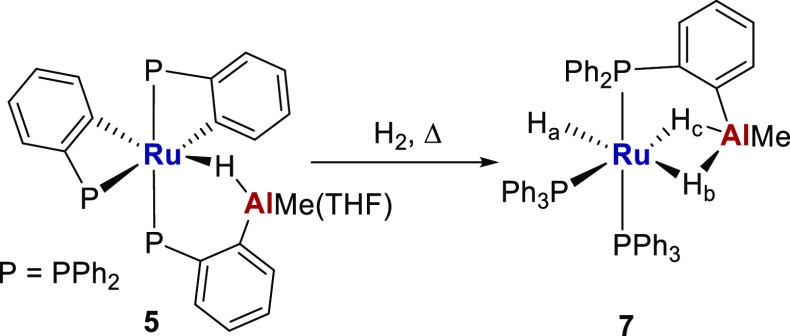
Proposed Structure of **7** from Reaction of **5** with H_2_ (1 atm, 60°C)

Complex **5** showed a similar reluctance to **1** and **2** in reacting with H_2_ only at elevated temperature (60 °C) to yield a single product, which was characterized as the trihydride species [Ru(PPh_2_)_2_{PPh_2_C_6_H_4_AlMe}H_3_] **7** (Scheme 7) based on NMR spectroscopy (Figures S17–S22).^21^ We were unable to crystallize the product which decomposed in the absence of a H_2_ atmosphere to a mixture of species, two of which were identified as [Ru(PPh_3_)_3_(η^2^-H_2_)H_2_] and [Ru(PPh_3_)_4_H_2_].^22^ The fate of the aluminum was not determined.

The ^31^P{^1^H} NMR spectrum of **7** exhibited three signals with *J*_PP_ values indicative of a *mer*-RuP_3_ geometry. Based on the findings for **5**, the high frequency (δ 74, doublet of doublets) signal was attributed to the Al-metalated phosphine, with a doublet of doublets at δ 62 and a triplet at δ 59 arising from the two PPh_3_ ligands. The low frequency region of the room temperature ^1^H NMR spectrum of **7** showed an Al*Me* resonance at δ −0.39, which integrated to 3 relative to three hydride signals (each of relative integral 1) at δ −8.46 (broad doublet), −8.72 (triplet of doublets) and −11.07 (doublet of triplets).^23,24^ The lowest frequency hydride signal was assigned to the bridging hydride H_c_ (Scheme 7) based on the presence of (i) a NOESY peak to the Al*Me* resonance and (ii) a 54 Hz ^2^*J*_HP_ doublet splitting, indicative of a pseudo-trans PPh_3_ ligand. The well-resolved appearance of this signal suggests it is more closely associated with Ru than quadrupolar Al. The highest frequency hydride signal was assigned to H_b_ based on the presence of a NOESY peak to the Al*Me* signal. The signal stayed broad between 223 and 337 K (Figure S19), consistent with it being associated more with Al (i.e., Ru···*H*-Al).^25^ The magnitude of the ^2^*J*_HP_ splittings (28 and 14 Hz) on the resonance at δ −8.72 (H_a_) support it being cis to three phosphine ligands.^26^ No ^2^*J*_HH_ coupling was observed on any of the hydride resonances in the ^1^H{^31^P} NMR spectrum.^27^

NMR spectra of the analogous reaction with D_2_ showed that the three hydride signals were present in the proton NMR spectrum, but all in an integral ratio of <1 relative to the Al*Me* resonance. This, together with broad ^31^P resonances, indicates that both H_2_/D_2_ addition as well as phosphine cyclometalation must be reversible, allowing H/D exchange to take place into the ortho-positions of PPh_3_ ligands.

**Scheme 8:**
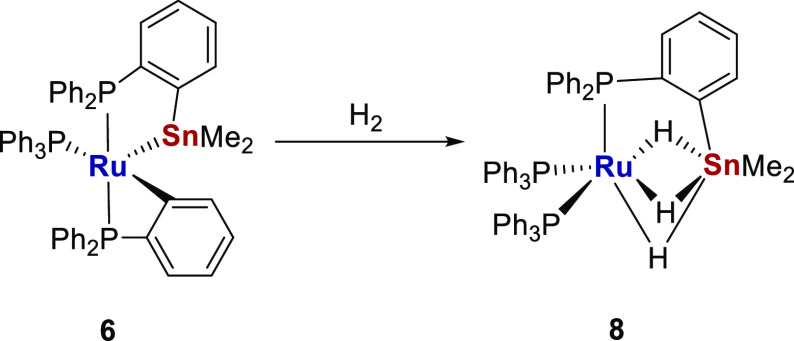
Proposed Structure of **8** from the Room Temperature Reaction of **6** with H_2_ (1 atm)

In contrast to **5**, [Ru–Sn] complex **6** showed behavior that aligned with [Ru–Zn] complex **3** in reacting with H_2_ at room temperature, to yield what we assign as the trihydride complex [Ru(PPh_2_)_2_{PPh_2_C_6_H_4_SnMe_2_}H_3_] **8** (Scheme 8). A gradual color change from a blue to a colorless/pale-yellow was observed when H_2_ was allowed to diffuse slowly through a sample of **6**, although if H_2_ was added and the sample shaken vigorously, a colorless precipitate (which failed to redissolve in most common solvents) formed almost instantaneously. The IR spectra of the precipitate and material from solution were identical,^28^ implying that **8** is the product in both cases. We assume that **8** sits right on the edge of solubility and that shaking results in precipitation.

The ^1^H NMR spectrum of a homogeneous solution of **8** formed upon slow diffusion of H_2_ exhibited a single Sn*Me*_2_ resonance (cf. two different Sn*Me* resonances for **6**) of integral 6 at room temperature, along with a single, broad (fwhm = 36 Hz) hydride signal at δ −7.6 of relative integral 3 (Figures S23–S27) with a ^1^*J*_HSn_ coupling of 184 Hz. The magnitude is suggestive of some degree of interaction between Ru-*H* and Sn centers,^29,30^ although the hydride *T*_1_ value of 390 ms (400 MHz, 298 K) would exclude any appreciable nonclassical behavior. Variable temperature NMR measurements were consistent with **8** being fluxional in solution. Thus, cooling to 223 K (THF) only broadened the hydride signal, whereas warming to 332 K resolved it into a single doublet of triplets, with *J*_HP_ values of 16 and 7 Hz, indicative of the hydride ligands being cis to all three phosphorus nuclei. The fluxionality was mirrored in the ^31^P{^1^H} NMR spectrum, which comprised at low temperature of a triplet (δ 85, cyclostannylated phosphine), together with a broad singlet (δ 56, two PPh_3_ ligands) that resolved into a doublet upon warming to (or above) room temperature. The mutual ^2^*J*_PP_ splitting of 98 Hz is in-between the values typically associated with trans- and cis–P–Ru−P arrangements.^31^

The ease with which **8** precipitated thwarted all attempts to generate single crystals suitable for X-ray crystallography, even via a solid-state transformation.^32^Scheme 8 shows a structure for **8** (Table S2) that is based on other group 8 metal derivatives [Ru(PR_3_)_3_(ER_3_′)H_3_] (ER_3_′ = SiR_3_′, SnR_3_′),^29a,30,33^ which all feature a common tetrahedral arrangement of Si/Sn and 3PR_3_ units with hydride ligands capping the Si/Sn(PR_3_)_2_ faces.

### Reactivity of **6** with Lewis Bases

#### CO

**Scheme 9:**
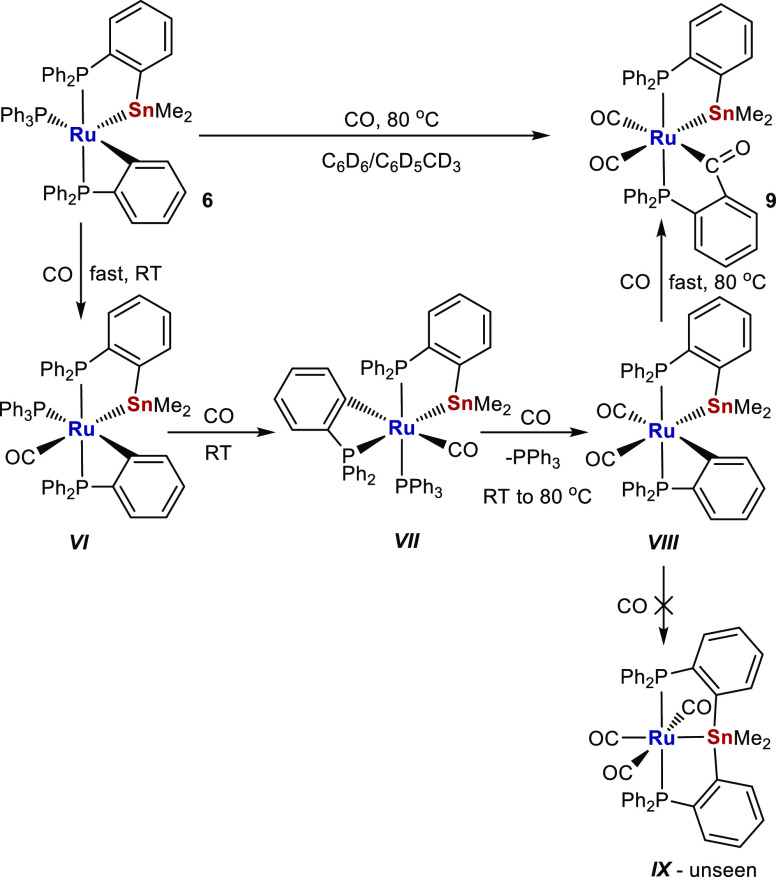
Spectroscopically Detected Intermediates ***VI***–***VIII*** in the Formation of **9**

Additional studies of small molecule reactivity focused on [Ru–Sn] precursor **6**. As shown in Scheme 9, both addition and insertion of CO took place when **6** was heated under 1 atm CO at 80 °C, to ultimately form the acylated phosphine complex [Ru(CO)_2_(C(O)C_6_H_4_PPh_2_)(PPh_2_C_6_H_4_SnMe_2_)] **9**, which could be isolated in 60% yield. Typically, acylated phosphine ligands are generated by oxidative addition of phosphino substituted aldehydes,^34^ rather than by CO insertion into a M–aryl bond,^35^ although the latter route does have precedence with ruthenium.^35b^

**Figure 2:**
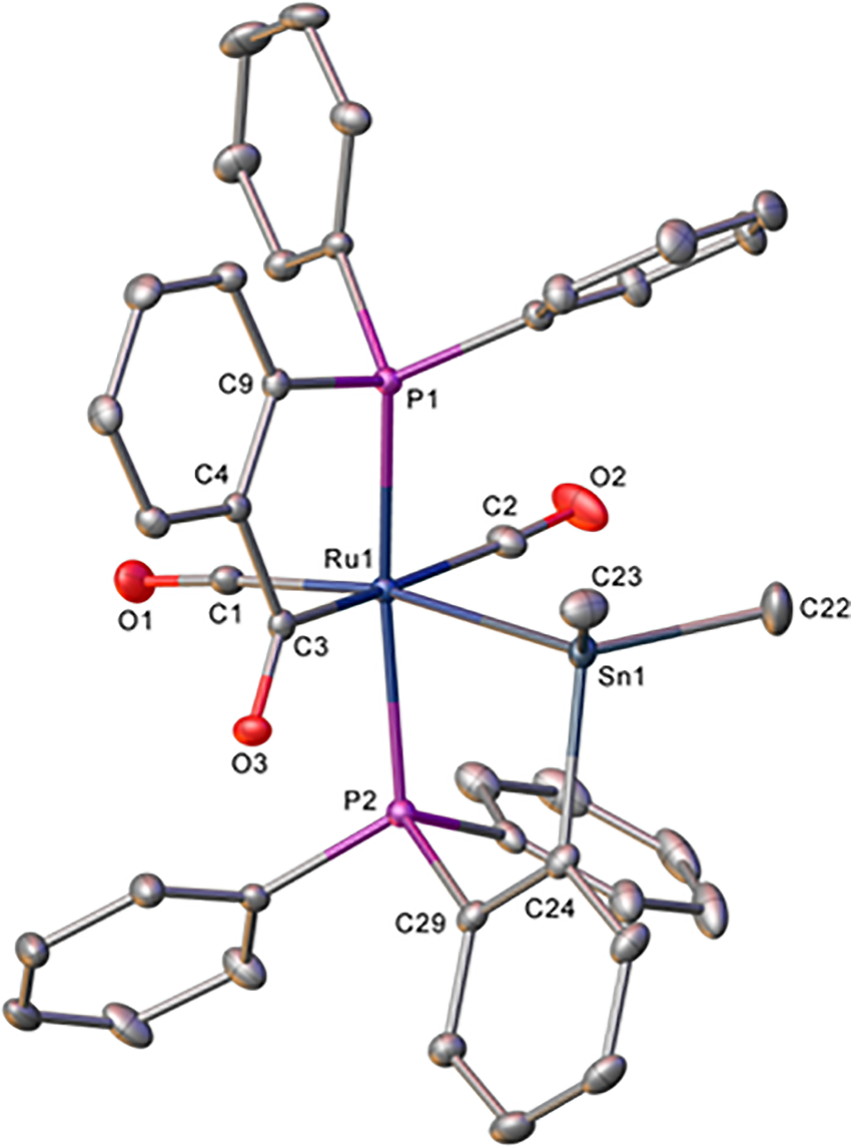
Molecular structure of **9**. Ellipsoids are shown at 30% level with all H atoms omitted for clarity. Selected bond lengths (Å) and angles (deg): Ru1–P1 2.3214(6), Ru1–P2 2.3869(6), Ru1–C1 1.920(2), Ru1–C2 1.942(2), Ru1–C3 2.129(2), Ru1–Sn1 2.6879(2), P1–Ru1–P2 170.79(2).

The X-ray structure (Figure 2) of **9** revealed an octahedral coordination sphere with a trans-arrangement of the P atoms of the stannylated and acylated phosphines, leaving the -SnMe_2_ and -C(O)(aryl) groups trans to the two carbonyl ligands. As a result of this geometry, the Ru–Sn (2.6879(2) Å) and Ru–P (2.3869(6) Å) distances of the stannylated phosphine were significantly longer than in **6**. The Ru–C(O) distance (2.129(2) Å) was comparable to that in [Ru(PPh_3_)(CO)_2_(C(O)C_6_H_4_PPh_2_)H] (2.110(1) Å).^34c^ In the ^31^P NMR spectrum, there was only a minor change in the chemical shift of the stannylated phosphine relative to **6**, whereas the phosphine metalated onto Ru moved ca. 100 ppm to higher frequency as a result of CO insertion (Figures S32–S35).^35b^

In situ NMR measurements, in conjunction with ^13^CO labeling, revealed a series of intermediates on the pathway to **9** (Scheme 9; Figures S37–S42). Thus, shaking **6** with ^13^CO (1 atm) brought about an instantaneous change in color at room temperature from blue to yellow, concomitant with formation of the 18-electron CO addition species ***VI***. This was identified by the appearance of three doublet of doublet of doublet ^31^P resonances, each with a cis-sized ^2^*J*_PC_ coupling (8–11 Hz) to a single ^13^CO ligand, which resonated in the ^13^C{^1^H} NMR spectrum at δ 207 as a doublet of triplets. Isomerization of ***VI*** occurred overnight at room temperature to yield ***VII***, which exhibited one metalated phosphorus signal with a much greater ^2^*J*_PSn_ splitting (970 Hz vs 180 Hz), consistent with a change in orientation to trans P–Ru–SnMe_2_. There was also a small amount of the dicarbonyl species ***VIII*** (identified on the basis of two multiply coupled high frequency signals in the ^13^C{^1^H} NMR spectrum), which increased in intensity upon heating at 80 °C, leaving it as the main product in solution after 1 h. Further heating converted ***VIII*** to the final product **9**. No signals attributable to ***IX*** (Scheme 9), a “Ru-SnPhos” analogue of [Ru(ZnPhos)(CO)_3_], which we have shown to be the product formed when a mixture of [Ru(PPh_3_)_3_HCl] and LiCH_2_TMS/ZnMe_2_ was heated under CO,^36^ were observed at any point in the overall reaction.

#### 1,3,4,5-Tetramethylimidazol-2-ylidene (IMe_4_)

**Scheme 10:**
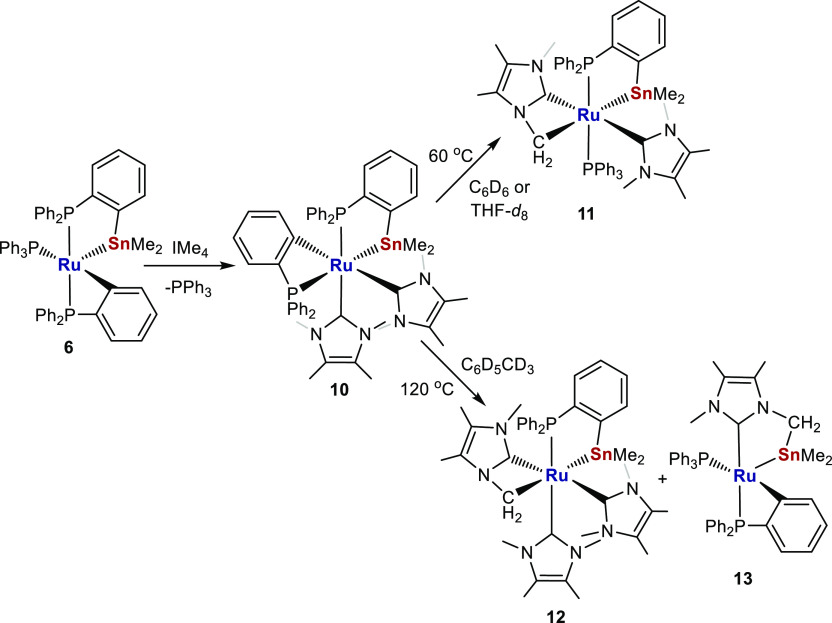
Synthesis of bis-IMe_4_ Complex **10** and Formation of **11**–**13** upon Heating

Treatment of **6** with ca. 3 equiv of the N-heterocyclic carbene 1,3,4,5-tetramethylimidazol-2-ylidene (IMe_4_) led to full consumption of the starting material over the course of ca. 1 h to form the coordinatively saturated product [Ru(IMe_4_)_2_(PPh_2_C_6_H_4_)(PPh_2_C_6_H_4_SnMe_2_)] **10** (Scheme 10), which was isolated as an orange microcrystalline solid in 55% yield. The ^31^P{^1^H} NMR spectrum of **10** displayed doublets at both high (δ 69) and low (δ −36) frequency, consistent with retention of both the cyclostannylated and cycloruthenated phosphines, although the magnitude of ^2^*J*_PP_ (18 Hz) now implied they were in a cis-configuration (Figures S43–S48). Two inequivalent IMe_4_ ligands were evident from the appearance of four N*Me* and four NC*Me* resonances in the ^1^H NMR spectrum and the presence of two ^13^C carbenic resonances (δ 191, ^2^*J*_CP_ = 86 and 16 Hz; δ 200, ^2^*J*_CP_ = 8 and 2 Hz).

**Figure 3:**
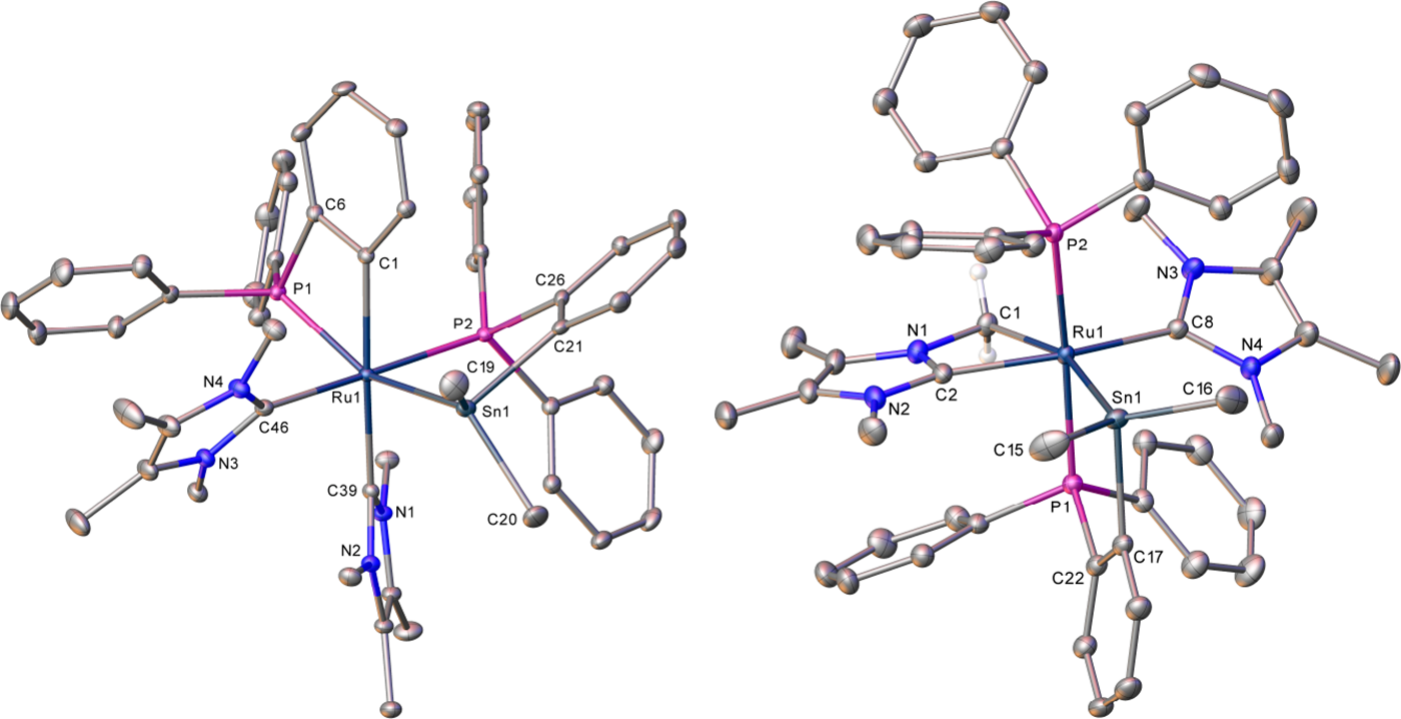
Molecular structures of one of the molecules in the asymmetric unit of (left) **10** and (right) **11**. Ellipsoids at 30% level. All H atoms and solvent have been omitted for clarity in **10**, while all hydrogens, with the exception of those attached to C1, have been omitted in **11**.

As shown in Figure 3 and Table 2, X-ray crystallography revealed that the Ru center in **10** was significantly distorted from regular octahedral. Accommodation of the two IMe_4_ ligands caused a reduction (relative to **6**) in the bite angles of both the cyclostannylated (84.371(17)° to 80.092(15)°) and cycloruthenated (67.93(7)° to 66.42(6)° respectively) phosphines, as well as an acute *trans*–P–Ru−Sn angle (155.047(15)°). Incorporation of a (phosphine) ligand trans to tin increased the Ru–Sn distance to 2.6345(2) Å from the value of 2.5686(2) Å in **6**.

**Table 2:** Selected Bond Lengths (Å) and Angles (deg) in the IMe_4_ Complexes **10**–**13**

	**10**	**11**	**12**	**13**
Ru-C_IMe4_	2.121(2), 2.132(2)	2.119(3)	2.109(2), 2.087(3)	-
Ru-C_IMe4′_	-	2.091(3)	2.089(3)	2.032(3)
Ru-CH_2_	-	2.246(3)	2.224(3)	-
Ru-*P*C_6_H_4_	2.4073(6)	-	-	2.3445(9)
Ru-*C*_6_H_4_P	2.132(2)	-	-	2.084(3)
Ru-*P*C_6_H_4_Sn	2.3451(5)	2.3162(8)	2.3267(6)	-
Ru-PPh_3_	-	2.3296(7)	-	2.3118(8)
Ru-Sn	2.6345(2)	2.6435(3)	2.6604(3)	2.5223(4)
C_IMe4_-Ru-C_IMe4_	88.50(8)	-	-	-
C_IMe4′_-Ru-Sn	-	99.05(9)	96.38(8)	81.25(11)

Heating **10** at 60 °C in THF or benzene brought about metalation of one of the IMe_4_ ligands to give [Ru(IMe_4_)(PPh_3_)(IMe_4_)′(PPh_2_C_6_H_4_SnMe_2_)] (**11**, Scheme 10). The ^31^P{^1^H} NMR spectrum showed replacement of the low frequency signal for the cycloruthenated phosphine in **10** by a resonance at δ 54, arising from a Ru-PPh_3_ resulting from reductive elimination of Ru–H (resulting from IMe_4_ activation) onto the Ph_2_PC_6_H_4_Ru ligand. The ^1^H NMR spectrum exhibited a total of seven carbene methyl resonances, and also showed two doublets of doublets at δ 2.42 and 2.22 (each of relative integral 1) for the diastereotopic protons of the Ru-CH_2_ arm (Figures S49–S51).

NMR monitoring of the reaction indicated that optimum conversion of the starting material (ca. 80–85%) occurred over ca. 2 h at 60 °C to yield **11** as the main reaction product, although always alongside a number of other, smaller, unidentifiable species, which became more prominent with longer heating. While we were therefore unable to isolate **11** as an analytically pure material, a combination of multinuclear NMR spectra and a crystal structure determination (achieved by picking of a single crystal) identified **11** unequivocally.

In contrast to the well-known metalation of NHCs bearing N-aryl or bulky N-alkyl substituents,^37^ C–H activation of N-methylated carbenes is restricted to a very small number of examples,^38^ most likely because of the severe structural constraints imposed by forming a four-membered ring metalacycle. These structural impositions are apparent in the X-ray structure of **11** (Figure 3), which shows a dramatically tilted carbene ring with very different N(1)–C(2)–Ru(1) and N(2)–C(2)–Ru(1) angles (99.3(2)° and 156.1(3)° respectively, Δ = 56.8°). The C1–Ru–C2 angle subtended at Ru (63.36(12)°) is more similar to that in [Os(P^*i*^Pr_3_)_2_(CO)(IMe_2_)′Cl] (63.01(16)/63.11(16)°) reported by Esteruelas^38b^ than [Tp^*t*Bu,Me^Yb(IMe_4_)(IMe_4_)′] (55.4(2)°) described by Ferrence et al.,^38a^ most likely due to the presence of both the bigger lanthanide and the very different ligand coordination spheres.

When **10** was heated to 120 °C in toluene, very different activation chemistry of the carbene took place with the IMe_4_ ligands from two molecules of **10** undergoing redistribution to give a mixture of the six-coordinate, tris-carbene product [Ru(IMe_4_)_2_(IMe_4_)′(PPh_2_C_6_H_4_SnMe_2_)] **12** and five-coordinate, monocarbene species [Ru(PPh_3_)(PPh_2_C_6_H_4_)(IMe_4_-SnMe_2_)′] **13** (Scheme 10). As for **11**, we were able to manually separate yellow crystals of **12** and purple crystals of **13** to allow their structural characterization, but were unable to separate enough clean material for elemental analyses or measurement of pristine NMR spectra. Purple **13** was more obvious to identify and manually separate, resulting in NMR spectra that were typically cleaner than those of **12** (Figures S52–S59).

**Figure 4:**
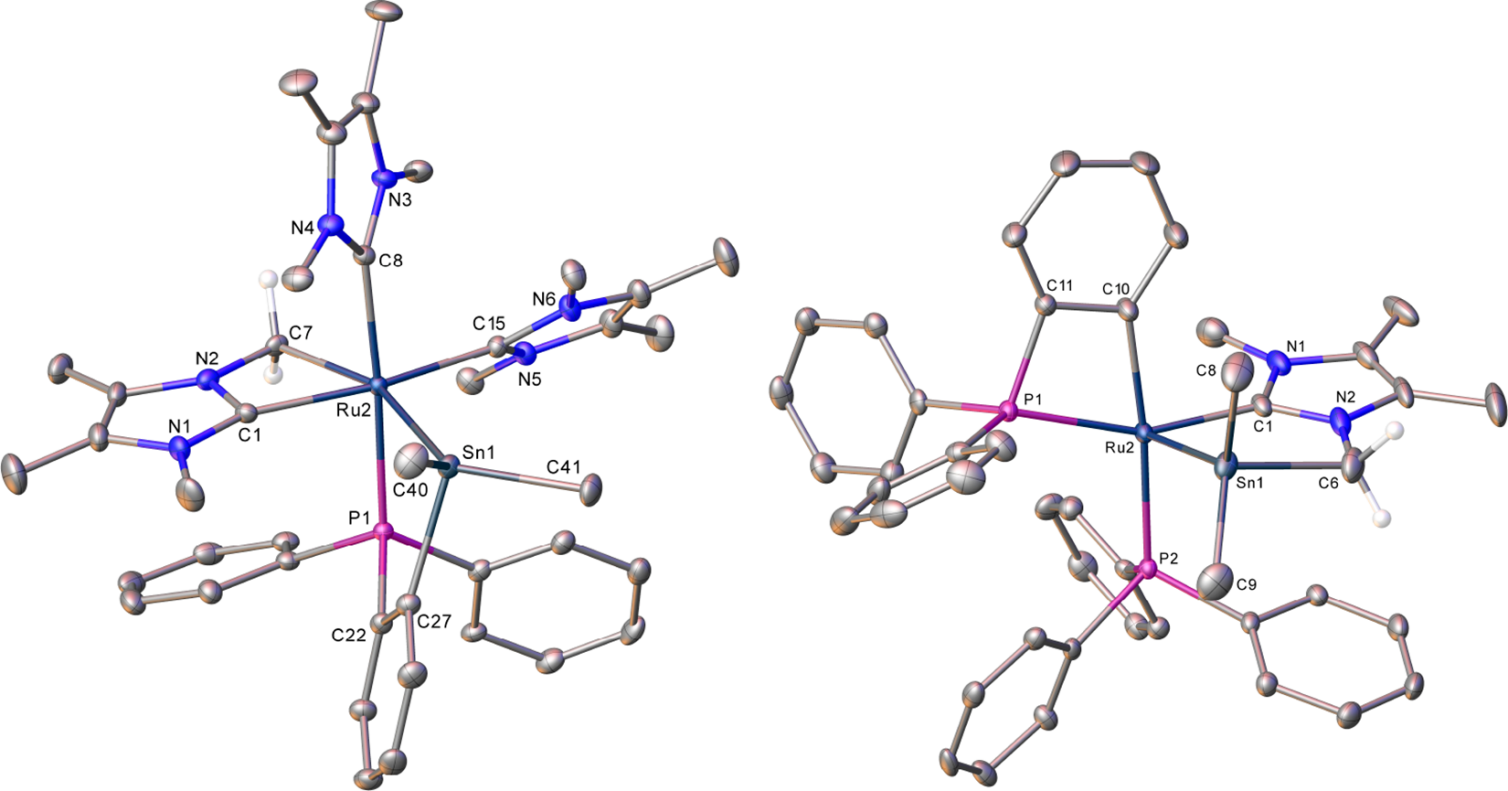
Molecular structures of one of the molecules in the asymmetric unit in (left) **12** and (right) **13**. Ellipsoids at 30% level. In **12**, the minor disordered component and hydrogens, with the exception of those attached to C7, have been omitted for clarity. In **13**, the minor disordered component and hydrogens, with the exception of those attached to C6, have been omitted for clarity.

The structure of **12**, which is shown in Figure 4 (metrics in Table 2), displayed a cis-arrangement of two intact IMe_4_ ligands, one of which was trans to the carbenic carbon of the third, metalated IMe_4_. The difference in the two Ru–C–N angles (Δ = 57.2°) showed that this was even more distorted in terms of coordination than that in **11**, although the bite angle did not change (C(1)–Ru(2)–C(7) = 63.47(11)°). A cyclostannylated phosphine occupied the last two coordination sites of the highly distorted (e.g., C(7)–Ru(2)–Sn(1) = 159.35(8)°) octahedral Ru coordination sphere. In accord with the structure, the ^1^H and ^13^C NMR spectra showed separate resonances for each of the 11 inequivalent N*Me* and NC*Me* groups.

The most striking feature of **13** (Figure 4, Table 2) was the formation of a novel chelating stannylcarbene ligand, attached to Ru at the apical and equatorial positions of a distorted square pyramid through very short Ru–C and Ru–Sn bond lengths (Ru(2)–C(1) = 2.032(3) Å, Ru(2)–Sn(1) = 2.5223(4) Å). While metal-bound NHCs with p-block functionalized N-substituents are quite common, they are typically preformed prior to either addition onto or substitution at a metal center,^39^ as opposed to through a bond activation reaction as seen here.^40^ We are unaware of any examples of bidentate NHC-Sn ligands prepared by any route,^41^ although Tilley has recently described a bidentate P–Sn ligand arising from C–H activation of an Fe-PMe^i^Pr_2_ ligand onto Sn.^42^

## Summary and Conclusions

The synthesis and reactivity of the heterobimetallic PPh_3_-derived [Ru–Al] and [Ru–Sn] complexes [Ru(C_6_H_4_PPh_2_)_2_{PPh_2_C_6_H_4_AlMe(THF)}H] **5** and [Ru(PPh_3_)(C_6_H_4_PPh_2_)(PPh_2_C_6_H_4_SnMe_2_)] **6** has been described in a study that represents a continuation of our ongoing research line, in which we attempt to describe and rationalize the effects of M′ on heterobimetallic Ru-main group metal M′ complexes. In conjunction with previous studies on [Ru–Li], [Ru–Mg] and [Ru–Zn] systems 1–3 (Scheme 1), we can conclude that

**Scheme 11:**
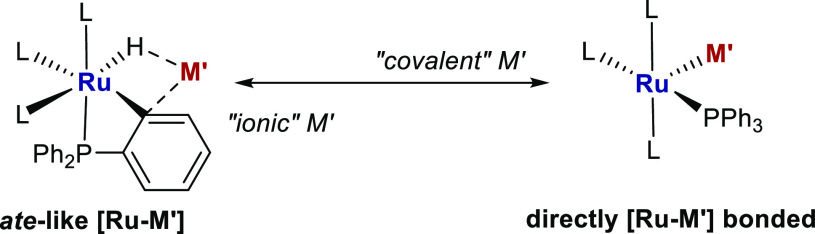
Representation of the Two Extremes of [Ru-M′] Complexes Arsing in Our Work

1. The nature of M′ strongly affects both the structure and reactivity of such heterobimetallic complexes, with *ate*-type chemistry predominant in the case of more “ionic” M′ metals such as Li, Mg and Al, whereas more “covalent” behavior is observed for M′ = Sn and Zn, with direct Ru–Sn (and Ru–Zn) bonds prevalent (Scheme 11).
2. Reactivity toward H_2_ provides a means to discriminate *ate*-[Ru-M′] from bonded [Ru-M′] type complexes; the former react sluggishly, while the latter react instantaneously, as a result of the presence of a coordinatively unsaturated Ru center.
3. Combining (reversibly) cyclometalated, and substitutionally labile, Ru-PPh_3_ ligands and an increasing number of M′-Me groups across M′ = Li, Mg, Zn, Al, and Sn provides a valuable route to heterobimetallic [Ru-M′] containing new ligand frameworks (e.g., chelating stannylcarbene ligand in **13**) with potential for further interesting small molecule reactivity.

## Experimental Section

### General Comments

All manipulations were carried out at room temperature under argon using standard Schlenk, high vacuum, and glovebox techniques using dry and degassed solvents. C_6_D_6_, C_6_D_5_CD_3_, and THF-*d*_8_ were vacuum transferred from potassium. NMR spectra were recorded at 298 K (unless otherwise stated) on Bruker Avance 400 and 500 MHz NMR spectrometers and referenced as follows: C_6_D_6_ (^1^H, δ 7.16; ^13^C, δ 128.0), C_6_D_5_CD_3_ (^1^H, δ 2.09), THF-*d*_8_ (^1^H, δ 3.58; ^13^C, δ 25.3). ^31^P{^1^H} spectra were referenced externally to 85% H_3_PO_4_ and ^119^Sn to SnMe_4_. IR spectra were recorded on a Nicolet Nexus spectrometer and UV–vis spectra on a PerkinElmer Lambda 35 spectrometer. Elemental analyses were performed by Elemental Microanalysis Ltd., Okehampton, Devon, U.K. [Ru(PPh_3_)_3_HCl]·toluene,^43^ [Ru(PPh_3_)(C_6_H_4_PPh_2_)_2_H][Li(THF)_2_] (**1**)^4^ and IMe_4_,^44^ were prepared according to literature methods. Prior to use, [Ru(PPh_3_)_3_HCl]·toluene was dried under high vacuum and ground to a fine powder affording a material with ca. 1 molecule of toluene per Ru (based on ^1^H NMR analysis). IMe_4_ was purified by sublimation. LiCH_2_TMS was used as a colorless solid obtained upon cooling a commercial 1.0 M solution in pentane at −32 °C, separating the resulting colorless crystals by decantation in a glovebox and drying under vacuum. AlMe_2_Cl (1.0 M solution in hexane, Merck) and SnMe_3_Cl (Merck) were used as received.

### [Ru(C_6_H_4_PPh_2_)_2_{PPh_2_C_6_H_4_AlMe(THF)}H] **5**

AlMe_2_Cl (135 μL of a 1.0 M solution in hexane, 0.135 mmol) was added to an agitated suspension of [Ru(PPh_3_)(C_6_H_4_PPh_2_)_2_H][Li(THF)_2_] (**1**, 94 mg, 0.09 mmol) and PPh_3_ (47 mg, 0.18 mmol)^45^ in benzene (1.5 mL), and the reaction mixture was heated at 60 °C for 1 h. After the mixture was cooled to room temperature, the precipitate of LiCl was separated by cannula filtration and the filtrate reduced to dryness. The residue was dissolved in THF (2 mL), layered with hexane (2 mL), and left to crystallize at −20 °C (3 days). The yellow crystalline product was separated by decantation, washed with hexane (2 × 1 mL), and dried under vacuum. Yield: 71 mg (69%; contains ca. 3 molecules of THF per Ru based on ^1^H NMR analysis; Figure S1). ^1^H NMR (500 MHz, C_6_D_6_): δ 8.32 (t, *J* = 9.0 Hz, 2H, Ar), 7.90 (d, *J* = 6.9 Hz, 1H, Ar), 7.84–7.74 (m, 4H, Ar), 7.50 (t, *J* = 6.9 Hz, 1H, Ar), 7.40 (br s, 1H, Ar), 7.30–7.14 (m, 7H, Ar; partially overlapped with residual C_6_D_5_H), 7.05−6.99 (m, 4H, Ar), 6.93 (br m, 2H, Ar), 6.88–6.78 (m, 5H, Ar), 6.75–6.61 (m, 10H, Ar), 6.45 (t, *J* = 7.9 Hz, *J* = 2.3 Hz, 2H, Ar), 6.06 (t, *J* = 9.0 Hz, 2H, Ar), 5.83 (br m, 1H, Ar), 3.56 (m, 11H, THF), 1.40 (m, 11H, THF), −1.00 (s, 3H, Al*Me*), −6.30 (m, 1H, Ru*H*). ^31^P{^1^H} NMR (202 MHz, C_6_D_6_): δ 70.0 (dd, ^2^*J*_PP_ = 266 Hz, ^2^*J*_PP_ = 23 Hz), −15.4 (dd, ^2^*J*_PP_ = 266 Hz, ^2^*J*_PP_ = 30 Hz), −25.9 (dd, ^2^*J*_PP_ = 30 Hz, ^2^*J*_PP_ = 23 Hz). Anal. Calcd. for C_55_H_46_AlP_3_Ru·2.75THF (1126.1): C 70.38, H 6.09. Found: C 70.49, H 6.28.

### [Ru(PPh_3_)(C_6_H_4_PPh_2_)(PPh_2_C_6_H_4_SnMe_2_)] **6**

A THF suspension (10 mL) of [Ru(PPh_3_)_3_HCl]·toluene (509 mg, 0.50 mmol) was treated with LiCH_2_TMS (97 mg, 1.03 mmol) and stirred for 30 min a J. Young resealable ampule to afford an orange solution. A solution of SnMe_3_Cl (100 mg in 3 mL C_6_H_6_, 0.50 mmol) was added dropwise over ca. 3 min with stirring (the vial containing the SnMe_3_Cl solution was washed with C_6_H_6_ (2 × 1 mL), and the washings added to the reaction). The resulting dark blue solution was stirred (2 h), and the volatiles were then removed under vacuum. The residual blue oil was treated with 20 mL hexane and 10 mL of benzene to precipitate LiCl. The suspension was cannula filtered, the residue washed with hexane (5 mL), and the combined filtrate and washings were concentrated under vacuum to yield a blue oil. Recrystallization from benzene/hexane (1:2 ratio) at room temperature for 24 h, and then at −20 °C for 72 h, afforded **6** as dark blue crystals, which were separated, washed with hexane (2 × 1 mL), and dried under vacuum to give 520 mg of product (94% yield). **6** is present in solution together with ca. 5% of a minor isomer, which we propose to have the structure **6′** shown in Figure S4. Data for **6**: ^1^H NMR (500 MHz, THF-*d*_8_): δ 7.74 (t, *J* = 6.5 Hz, 1H, Ar), 7.66 (t, *J* = 8.4 Hz, 2H, Ar), 7.58 (d, *J* = 6.9 Hz (*J*_HSn_ = 25.0 Hz), 1H, Ar), 7.47 (t, *J* = 8.8 Hz, 2H, Ar), 7.34–7.16 (m, 9H (partially overlaps with C_6_H_6_), Ar), 7.14–6.99 (m, 5H, Ar), 6.95–6.84 (m, 14H, Ar), 6.77–6.66 (m, 2H, Ar), 6.64 (t, *J* = 7.3 Hz, 1H, Ar), 6.55 (t, *J* = 7.5 Hz, 2H, Ar), 6.40–6.28 (m, 4H, Ar), 0.40 (s (^2^*J*_HSn_ = 41 Hz), 3H, Sn*Me*), −1.10 (s (^2^*J*_HSn_ = 46 Hz), 3H, Sn*Me*). ^31^P{^1^H} NMR (202 MHz, THF-*d*_8_): δ 74.8 (dd, ^2^*J*_PP_ = 241 Hz, ^2^*J*_PP_ = 16 Hz (^2^*J*_PSn_ = 150 Hz)), 39.9 (dd, ^2^*J*_PP_ = 25 Hz, ^2^*J*_PP_ = 16 Hz (^2^*J*_PSn_ = 76 Hz)), −28.5 (dd, ^2^*J*_PP_ = 241 Hz, ^2^*J*_PP_ = 25 Hz (^2^*J*_PSn_ = 148 Hz)). ^119^Sn{^1^H} NMR (187 MHz, THF-*d*_8_): δ 21.4 (td, ^2^*J*_SnP_ = 150 Hz, ^2^*J*_SnP_ = 78 Hz). UV/vis (toluene, nm): λ_max_ = 600 (ε = 2080 dm^3^ mol^–1^ cm^–1^), 486 (ε = 1890 dm^3^ mol^–1^ cm^–1^). Anal. Calcd. for C_56_H_49_P_3_RuSn·C_6_H_6_ (1112.8): C 66.92, H 4.98. Found: C 68.36, H 5.17. Repeated attempts at analysis gave consistently a high%C value, which might be attributable to an incorrect formulation for **6′**. Selected NMR data for **6′**. ^1^H NMR (500 MHz, THF-*d*_8_): δ 0.11 (s (^2^*J*_HSn_ = 47 Hz), 3H, Sn*Me*), −0.20 (s (^2^*J*_HSn_ = 44 Hz), 3H, Sn*Me*). ^31^P{^1^H} NMR (202 MHz, THF-*d*_8_): δ 50.1 (dd, ^2^*J*_PP_ = 243 Hz, ^2^*J*_PP_ = 17 Hz), 37.4 (dd, ^2^*J*_PP_ = 27 Hz, ^2^*J*_PP_ = 17 Hz), 3.5 (dd, ^2^*J*_PP_ = 243 Hz, ^2^*J*_PP_ = 27 Hz).

### Variable Temperature NMR Study of the Formation of **5**

AlMe_2_Cl (52 μL of 1.0 M hexane solution, 0.05 mmol) was vacuum transferred into a J. Young resealable NMR tube containing a THF-*d*_8_ (0.5 mL) solution of **1** (11 mg, 0.01 mmol). The yellow-orange solution was maintained at 193 K prior to insertion into a precooled (193 K) NMR spectrometer. ^1^H, ^31^P{^1^H}, ^1^H{^31^P}, and ^1^H–^31^P HMQC NMR spectra acquired over the temperature range of 193–298 K (Figures S8–S12) showed the formation of intermediates ***I***–***IV***. Selected ^1^H NMR data for ***I***. ^1^H NMR (400 MHz, THF-*d*_8_, 193 K): δ −10.49 (ddd, ^2^*J*_HP_ = 45 Hz, 20 Hz, 5 Hz, 1H, Ru*H*).^46^^31^P{^1^H} NMR (162 MHz, THF-*d*_8_, 193 K): δ 46.6 (br), −27.3 (br), −28.5 (t, ^2^*J*_PP_ = 21 Hz). ^1^H NMR (400 MHz, THF-*d*_8_, 233 K): δ −10.18 (ddd, ^2^*J*_HP_ = 49.7, ^2^*J*_HP_ = 21.8 Hz, ^2^*J*_HP_ = 8.0 Hz, 1H, Ru*H*). ^31^P{^1^H} NMR (162 MHz, THF-*d*_8_, 233 K): δ 51.1 (t, ^2^*J*_PP_ = 21 Hz), −22.6 (t, ^2^*J*_PP_ = 18 Hz), −30.9 (t, ^2^*J*_PP_ = 21 Hz). Selected ^1^H NMR data for ***II***. ^1^H NMR (400 MHz, THF-*d*_8_, 193 K): δ −0.58 (br t, ^3^*J*_HP_ = 7 Hz, 3H, Ru*Me*), −13.92 (apparent dd, ^2^*J*_HP_ = 47 Hz, ^2^*J*_HP_ = 19 Hz, 1H, Ru*H*).^53^^31^P{^1^H} NMR (162 MHz, THF-*d*_8_, 193 K): δ 51.1 (br), 50.3 (br), −33.2 (t, ^2^*J*_PP_ = 19 Hz). ^1^H NMR (400 MHz, THF-*d*_8_, 233 K): δ −0.80 (Ru*Me*, overlapped with Al*Me* signals, based on ^31^P HMQC), −13.73 (ddd, ^2^*J*_HP_ = 49.7 Hz, ^2^*J*_HP_ = 19.4 Hz, ^2^*J*_HP_ = 6.9 Hz, 1H, RuH). ^31^P{^1^H} NMR (162 MHz, THF-*d*_8_, 233 K): δ 56.5 (t, ^2^*J*_PP_ = 21 Hz), 48.6 (t, ^2^*J*_PP_ = 17 Hz), −35.5 (dd, ^2^*J*_PP_ = 23 Hz, ^2^*J*_PP_ = 17 Hz). Selected ^1^H NMR data for ***III***. ^1^H NMR (400 MHz, THF-*d*_8_, 233 K): δ 5.51 (t, *J* = 7.8 Hz, 2H, Ar), −1.00 (s, 3H, Al*Me*), −2.02 (s, 3H, Al*Me*), −12.20 (dt, ^2^*J*_HP_ = 56.9 Hz, ^2^*J*_HP_ = 18.4 Hz, 1H, Ru*H*). ^31^P{^1^H} NMR (162 MHz, THF-*d*_8_, 233 K): δ 56.7 (dd, ^2^*J*_PP_ = 252 Hz, ^2^*J*_PP_ = 25 Hz), 48.7 (dd, ^2^*J*_PP_ = 252 Hz, ^2^*J*_PP_ = 22 Hz), −36.1 (t, ^2^*J*_PP_ = 24 Hz). Selected ^1^H NMR data for ***IV***. ^1^H NMR (400 MHz, THF-*d*_8_, 273 K): δ −0.45 (s, 3H, Al*Me*). ^31^P{^1^H} NMR (162 MHz, THF-*d*_8_, 273 K): δ 67.4 (dd, ^2^*J*_PP_ = 228 Hz, ^2^*J*_PP_ = 17 Hz), 46.9 (dd, ^2^*J*_PP_ = 26 Hz, ^2^*J*_PP_ = 17 Hz), −31.0 (dd, ^2^*J*_PP_ = 228 Hz, ^2^*J*_PP_ = 26 Hz).

### Variable Temperature NMR Study of the Formation of **6**

SnMe_3_Cl (2.5 mg, 0.012 mmol, in 0.1 mL THF-*d*_8_) was injected into a J. Young resealable NMR tube containing a frozen THF-*d*_8_ (0.4 mL) solution of **1** (11.5 mg, 0.011 mmol). The reaction mixture was warmed until the THF melted (165 K), at which point the color changed from yellow-orange to dark blue. The blue solution was inserted into a precooled (193 K) NMR spectrometer and ^1^H, ^31^P{^1^H} and ^1^H{^31^P} NMR spectra were acquired over the range 193–233 K. A single species assigned as ***V*** (Scheme 4) was observed up to 273 K, at which point, the final product **6** was also observed (Figures S13–S16). Selected NMR data for ***V***. ^1^H NMR (400 MHz, THF-*d*_8_, 193 K): δ −0.21 (s (^2^*J*_HSn_ = 38 Hz), 9H, Sn*Me*_3_). ^31^P{^1^H} NMR (162 MHz, THF-*d*_8_, 193 K): δ 48.6 (dd, ^2^*J*_PP_ = 241 Hz, ^2^*J*_PP_ = 14 Hz (^2^*J*_PSn_ = 177 Hz)), 40.6 (dd, ^2^*J*_PP_ = 24 Hz, ^2^*J*_PP_ = 14 Hz (^2^*J*_PSn_ = 155 Hz)), −36.1 (dd, ^2^*J*_PP_ = 241 Hz, ^2^*J*_PP_ = 24 Hz (^2^*J*_PSn_ = 198 Hz)).

### [Ru(PPh_3_)_2_(PPh_2_C_6_H_4_AlMe)H_3_] **7**

A C_6_D_6_ (0.5 mL) solution of **5** (10 mg, 0.009 mmol) in a J. Young resealable NMR tube was freeze–pump–thaw degassed (×3) and placed under 1 atm H_2_. Heating at 60 °C for 2 h resulted in complete conversion (based on ^31^P{^1^H} NMR spectroscopy) to [Ru(PPh_3_)_2_(PPh_2_C_6_H_4_AlMe)H_3_] **7**, which was characterized by ^1^H and ^31^P{^1^H} NMR spectroscopy. Selected NMR data for **7**: ^1^H NMR (500 MHz, C_6_D_6_): δ −0.39 (s, 3H, Al*Me*), −8.46 (br d, 1H, Ru···*H*-Al), −8.72 (td, ^2^*J*_HP_ = 28.2 Hz, ^2^*J*_HP_ = 13.9 Hz, 1H, Ru-*H*), −11.07 (dt, ^2^*J*_HP_ = 53.5 Hz, ^2^*J*_HP_ = 23.3 Hz, 1H, Ru-*H*···Al). ^31^P{^1^H} NMR (202 MHz, C_6_D_6_): δ 73.5 (dd, ^2^*J*_PP_ = 240 Hz, ^2^*J*_PP_ = 28 Hz), 62.1 (dd, ^2^*J*_PP_ = 240 Hz, ^2^*J*_PP_ = 23 Hz), 58.8 (br t, ^2^*J*_PP_ = 25 Hz). Exposure of the sample to vacuum for 2 h followed by redissolution of the residue in C_6_D_6_ revealed complete degradation of **7** and appearance of a number of resonances in both the ^1^H and ^31^P NMR spectra (Figure S22), including signals for both [Ru(PPh_3_)_3_(η^2^-H_2_)H_2_] (^1^H: δ −7.08 (s); ^31^P{^1^H}: δ 57.7 (s)) and [Ru(PPh_3_)_4_H_2_] (^1^H: δ −10.13 (m); ^31^P{^1^H}: δ 49.3 (t), 41.1 (t)).^25^ The fate of the Al metal was not established.

### [Ru(PPh_3_)_2_(PPh_2_C_6_H_4_SnMe_2_)H_3_] **8**

A C_6_D_6_ (0.5 mL) or THF-*d*_8_ (0.5 mL) solution of **6** (12 mg, 0.011 mmol) in a J. Young resealable NMR tube was placed under 1 atm of H_2_, which was then allowed to slowly diffuse through the sample to yield a pale yellow-colorless homogeneous solution of [Ru(PPh_3_)_2_(PPh_2_C_6_H_4_SnMe_2_)H_3_] **8**. This was characterized by ^1^H and ^31^P{^1^H} variable temperature NMR spectroscopy. ^1^H NMR (500 MHz, C_6_D_6_): δ 8.0 (d, *J*_HH_ = 7.0 Hz (*J*_HSn_ = 32 Hz), 1H, Ar), 7.51 (dd, *J*_HH_ = 7.8, *J*_HH_ = 4.5 Hz, 1H, Ar), 7.39 (t, *J*_HH_ = 8.9 Hz, 4H, Ar), 7.28 (t, *J*_HH_ = 8.8 Hz, 12H, Ar), 7.02 (t, *J* = 7.3 Hz, 1H, Ar), 6.94 (t, *J*_HH_ = 7.6 Hz, 6H, Ar), 6.88–6.79 (m, 18H, Ar), −0.50 (s (^2^*J*_HSn_ = 47 Hz), 6H, Sn*Me*), −7.51 (br m (^2^*J*_HSn_ = 183 Hz), 3H, Ru*H*). ^31^P{^1^H} NMR (162 MHz, C_6_D_6_): δ 84.7 (t, ^2^*J*_PP_ = 99 Hz (^2^*J*_PSn_ = 124 Hz)), 56.6 (d, ^2^*J*_PP_ = 99 Hz (^2^*J*_PSn_ = 95 Hz)). ^1^H NMR (500 MHz, THF-*d*_8_): δ 7.89 (d, *J*_HH_ = 7.3 Hz (*J*_HSn_ = 33 Hz), 1H, Ar), 7.39 (m, 1H, Ar), 7.13 (t, *J*_HH_ = 7.2 Hz, 7H, Ar), 7.08 (t, *J*_HH_ = 9.0 Hz, 3H, Ar), 7.04–6.89 (m, 23H, Ar), 0.05 (s (^2^*J*_HSn_ = 48 Hz), 6H, Sn*Me*), −7.91 (br m (^2^*J*_HSn_ = 179 Hz), 3H, Ru*H*). ^31^P{^1^H} NMR (162 MHz, THF-*d*_8_): δ 85.1 (t, ^2^*J*_PP_ = 97 Hz (^2^*J*_PSn_ = 127 Hz)), 56.2 (d, ^2^*J*_PP_ = 97 Hz (*J*_PSn_ = 89 Hz)). Selected ^1^H NMR (500 MHz, THF-*d*_8_, 332 K): δ 0.04 (s (^2^*J*_HSn_ = 48 Hz), 6H, Sn*Me*), −7.95 (dt, ^2^*J*_HP_ = 16.4 Hz, ^2^*J*_HP_ = 7.4 Hz (^2^*J*_HSn_ = 180 Hz), 3H, Ru*H*). ^31^P{^1^H} NMR (162 MHz, THF-*d*_8_, 332 K): δ 84.0 (t, ^2^*J*_PP_ = 96 Hz (*J*_PSn_ = not determined)), 54.6 (d, ^2^*J*_PP_ = 96 Hz (*J*_PSn_ ∼ 96 Hz)). IR (KBr, cm^–1^): 1967 (ν_RuHSn_), 1746 (ν_RuHSn_).

The formation of **8** could also be performed in the solid-state. Stirring a microcrystalline sample of **6** (15 mg, 0.013 mmol) under 1 atm of H_2_ in a J. Young resealable ampule for 24 h brought about a color change from purple to off-white. Conversion to **8** was proven by IR spectroscopy (Figure S30). In an attempt to prepare an isolable derivative of **8**, an excess of pyridine (50 μL, 0.49 mmol) was added to a C_6_D_6_ (0.5 mL) solution of **6** (10 mg, 0.022 mmol) to give [Ru(NC_5_H_5_)(C_6_H_4_PPh_2_)(PPh_2_C_6_H_4_SnMe_2_)] (Figure S31), assigned from the appearance of two doublets (δ 80.1 (d, ^2^*J*_PP_ = 285 Hz), −26.1 (d, ^2^*J*_PP_ = 285 Hz)) in the ^31^P{^1^H} NMR spectrum. Addition of 1 atm H_2_ to the crude sample rapidly yielded ^31^P{^1^H} NMR signals of **8** at ca. δ 84 and 55.

### [Ru(CO)_2_(C(O)C_6_H_4_PPh_2_)(PPh_2_C_6_H_4_SnMe_2_)] **9**

A benzene (2 mL) solution of **6** (111 mg, 0.10 mmol) was placed under CO (1 atm), and the solution was stirred at 80 °C for 4 h. The resulting yellow solution was filtered through a pad of Celite. The pad was washed with 1 mL C_6_H_6_ and the combined filtrate and washings layered with hexane (6 mL). An initial batch of yellow crystals of **9** were formed. Treatment with additional hexane (6 mL) and cooling to −32 °C for 24 h afforded yellow crystalline needles. The yellow solids were combined, washed with hexane (2 × 1 mL) and dried under vacuum. Yield: 54 mg (63%). ^1^H NMR (400 MHz, C_6_D_6_): δ 8.30–8.23 (m, 2H, Ar), 7.89–7.79 (m, 3H, Ar), 7.74 (d, *J* = 7.3 Hz (*J*_HSn_ = 26 Hz), 1H, Ar), 7.58–7.46 (m, 3H, Ar), 7.42 (t, *J* = 7.2 Hz, 1H, Ar), 7.27–6.87 (m, 18H, Ar), 0.24 (s (^2^*J*_HSn_ = 41 Hz), 3H, Sn*Me*), −0.24 (s (^2^*J*_HSn_ = 43 Hz), 3H, Sn*Me*). ^31^P{^1^H} NMR (162 MHz, C_6_D_6_): δ 66.9 (d, ^2^*J*_PP_ = 210 Hz (^2^*J*_P-117Sn_ = 140 Hz, ^2^*J*_P-119Sn_ = 146 Hz)), 64.2 (d, ^2^*J*_PP_ = 210 Hz (^2^*J*_P-117Sn_ = 134, ^2^*J*_P-119Sn_ = 140 Hz)). ^13^C{^1^H} NMR (126 MHz, C_6_D_6_): δ 202.9 (t, ^2^*J*_CP_ = 8 Hz, Ru-*C*O), 200.0 (t, ^2^*J*_CP_ = 10 Hz, Ru-*C*O), 158.1 (d, *J*_CP_ = 40 Hz, Ar), 155.5 (dd, *J*_CP_ = 61 Hz, *J*_CP_ = 4 Hz, Ar), 140.7 (d, *J*_CP_ = 42 Hz, Ar), 140.1 (d, *J*_CP_ = 4 Hz, Ar), 139.6 (br m, Ar), 139.3 (br m, Ar), 138.0 (dd, *J*_CP_ = 42 Hz, *J*_CP_ = 4 Hz, Ar), 136.2 (d, *J*_CP_ = 24 Hz, Ar), 135.5 (d, *J*_CP_ = 9 Hz, Ar), 134.8 (dd, *J*_CP_ = 39 Hz, *J*_CP_ = 3 Hz, Ar), 132.9 (d, *J*_CP_ = 13 Hz, Ar), 132.6 (d, *J*_CP_ = 9 Hz, Ar), 132.5 (d, *J*_CP_ = 9 Hz, Ar), 131.7 (d, *J*_CP_ = 13 Hz, Ar), 131.6 (d, *J*_CP_ = 11 Hz, Ar), 130.6 (d, *J*_CP_ = 9 Hz, Ar), 130.4 (d, *J*_CP_ = 5 Hz, Ar), 130.1 (s, Ar), 129.9 (s, Ar), 129.4 (s, Ar), 128.7 (d, *J*_CP_ = 11 Hz, Ar), 122.0 (d, *J*_CP_ = 17 Hz, Ar), −5.8 (s, Sn*Me*), −8.2 (s, Sn*Me*). ^119^Sn{^1^H} NMR (187 MHz, C_6_D_6_): δ 115.3 (t, ^2^*J*_SnP_ = 143 Hz). IR (KBr, cm^–1^): 2010 (ν_CO_), 1966 (ν_CO_), 1963 (ν_CO_), 1954 (ν_CO_), 1596 (ν_C(O)C6H4_), 1568 (ν_C(O)C6H4_). IR (C_6_D_6_, cm^–1^): 2008 (ν_CO_), 1961 (ν_CO_). Anal. Calcd. for C_41_H_34_O_3_P_2_RuSn (856.4): C 57.50, H 4.00. Found: C 57.88, H 4.09.

### Variable Temperature/^13^CO NMR Study of the Formation of **9**

A C_6_D_6_ (0.5 mL) solution of **6** (11 mg, 0.010 mmol) was placed under 1 atm ^13^CO. Upon shaking, an instantaneous color change from dark blue to yellow took place. ^1^H, ^31^P{^1^H} and ^13^C{^1^H} NMR spectroscopy was used to follow the progress of the reaction and allow characterization of intermediates ***VI–*^*13*^*CO***, ***VII–*^*13*^*CO***, and ***VIII–*^*13*^*CO***, initially over 20 h at room temperature, and then at 80 °C. The reaction was repeated using ^12^CO (1 atm) with 10 mg **6** in 0.5 mL C_6_D_5_CD_3_ to afford spectra of non-^13^CO labeled ***VI***, ***VII*** and ***VIII***. ***VI–*^*13*^*CO***. Selected ^1^H NMR (400 MHz, C_6_D_6_): δ 0.67 (s (^2^*J*_HSn_ = 29 Hz), 3H, Sn*Me*), −0.25 (s (^2^*J*_HSn_ = 35 Hz), 3H, Sn*Me*). ^31^P{^1^H} NMR (162 MHz, C_6_D_6_): δ 76.1 (ddd, ^2^*J*_PP_ = 241 Hz, ^2^*J*_PP_ = 15 Hz, ^2^*J*_PC_ = 11 Hz), 40.2 (ddd, ^2^*J*_PP_ = 24 Hz, ^2^*J*_PP_ = 15 Hz, ^2^*J*_PC_ = 8 Hz), −35.2 (ddd, ^2^*J*_PP_ = 241 Hz, ^2^*J*_PP_ = 24 Hz, ^2^*J*_PC_ = 10 Hz). Selected ^13^C{^1^H} NMR (101 MHz, C_6_D_6_): δ 206.5 (td, ^2^*J*_CP_ = 11 Hz, ^2^*J*_CP_ = 8 Hz, Ru-*C*O). ***VI***: ^31^P{^1^H} NMR (202 MHz, C_6_D_5_CD_3_): δ 76.1 (dd, ^2^*J*_PP_ = 241 Hz, ^2^*J*_PP_ = 15 Hz (^2^*J*_PSn_ = 174 Hz)), 40.2 (dd, ^2^*J*_PP_ = 23 Hz, ^2^*J*_PP_ = 15 Hz (^2^*J*_PSn_ = 163 Hz)), −35.2 (dd, ^2^*J*_PP_ = 241 Hz, ^2^*J*_PP_ = 24 Hz (^2^*J*_PSn_ = 182 Hz)). ***VII–*^*13*^*CO***. Selected ^1^H NMR (400 MHz, C_6_D_6_): δ 0.62 (s (^2^*J*_HSn_ = 39 Hz), 3H, Sn*Me*), 0.18 (s (^2^*J*_HSn_ = 39 Hz), 3H, Sn*Me*). ^31^P{^1^H} NMR (162 MHz, C_6_D_6_): δ 58.9 (ddd, ^2^*J*_PP_ = 254 Hz, ^2^*J*_PP_ = 18 Hz, ^2^*J*_PC_ = 9 Hz), 43.4 (ddd, ^2^*J*_PP_ = 254 Hz, ^2^*J*_PP_ = 28 Hz, ^2^*J*_PC_ = 15 Hz), −33.7 (ddd, ^2^*J*_PP_ = 28 Hz, ^2^*J*_PP_ = 18 Hz, ^2^*J*_PC_ = 4 Hz). Selected ^13^C{^1^H} NMR (101 MHz, C_6_D_6_): δ 207.3 (ddd, ^2^*J*_CP_ = 15 Hz, ^2^*J*_CP_ = 9 Hz, ^2^*J*_CP_ = 4 Hz, Ru-*C*O). ***VII***. ^31^P{^1^H} NMR (202 MHz, C_6_D_5_CD_3_): δ 58.9 (dd, ^2^*J*_PP_ = 254 Hz, ^2^*J*_PP_ = 18 Hz (^2^*J*_PSn_ = 173 Hz)), 43.4 (dd, ^2^*J*_PP_ = 254 Hz, ^2^*J*_PP_ = 28 Hz (^2^*J*_PSn_ = 196 Hz)), −33.7 (dd, ^2^*J*_PP_ = 28 Hz, ^2^*J*_PP_ = 18 Hz (^2^*J*_PSn_ = 970 Hz)). ***VIII–*^*13*^*CO***. Selected ^1^H NMR (400 MHz, C_6_D_6_): δ 0.57 (s (^2^*J*_HSn_ obscured by overlap with other signals), 3H, Sn*Me*), −0.64 (s (^2^*J*_HSn_ = 44 Hz), 3H, Sn*Me*). ^31^P{^1^H} NMR (162 MHz, C_6_D_6_): δ 69.5 (ddd, ^2^*J*_PP_ = 227 Hz, ^2^*J*_PC_ = 9 Hz, ^2^*J*_PC_ = 7 Hz), −29.2 (dt, ^2^*J*_PP_ = 227 Hz, ^2^*J*_CP_ = 9 Hz). Selected ^13^C{^1^H} NMR (101 MHz, C_6_D_6_): δ 202.8 (m, Ru-*C*O), 200.4 (td, ^2^*J*_CP_ = 9 Hz, ^2^*J*_CC_ = 3 Hz, Ru-*C*O). ***VIII***. ^31^P{^1^H} NMR (202 MHz, C_6_D_5_CD_3_): δ 69.5 (d, ^2^*J*_PP_ = 228 Hz (^2^*J*_PSn_ = 143 Hz)), −29.2 (d, ^2^*J*_PP_ = 228 Hz (^2^*J*_PSn_ = 141 Hz)).

### [Ru(IMe_4_)_2_(C_6_H_4_PPh_2_)(PPh_2_C_6_H_4_SnMe_2_)] **10**

IMe_4_ (17 mg, 0.13 mmol) was added to an agitated blue solution of **6** (54 mg, 0.048 mmol) in benzene (3 mL). The resulting yellow-orange solution was stirred for 1 h and then treated with hexane (3 mL) and left to crystallize for 24 h. The yellow-orange crystals of product were separated and dried under vacuum. Yield: 29 mg (55%). ^1^H NMR (500 MHz, THF-*d*_8_): δ 7.65–7.58 (m, 1H, Ar), 7.37–7.58 (m, 3H, Ar, overlapped with C_6_H_6_), 7.19–7.13 (m, 1H, Ar), 7.06–6.95 (m, 5H, Ar), 6.93–6.40 (m, 17H, Ar), 6.36 (ddd, *J* = 9.1 Hz, *J* = 7.1 Hz, *J* = 1.5 Hz, 1H, Ar), 3.83 (s, 3H, N*Me*), 3.73 (s, 3H, N*Me*), 3.16 (s, 3H, N*Me*), 2.43 (s, 3H, N*Me*), 2.11 (s, 3H, NC*Me*), 2.00 (s, 3H, NC*Me*), 1.91 (s, 3H, NC*Me*), 1.39 (s, 3H, NC*Me*), 0.54 (s (^2^*J*_HSn_ = 24 Hz), 3H, Sn*Me*), −0.23 (s (^2^*J*_HSn_ = 23 Hz), 3H, Sn*Me*). ^31^P{^1^H} NMR (162 MHz, THF-*d*_8_): δ 69.1 (d, ^2^*J*_PP_ = 18 Hz (^2^*J*_P-119Sn_ = 257 Hz, ^2^*J*_P-117Sn_ = 220 Hz)), −36.4 (d, ^2^*J*_PP_ = 18 Hz (^2^*J*_P-119Sn_ = 1311 Hz, ^2^*J*_P-117Sn_ = 1254 Hz)). Selected ^13^C{^1^H} NMR (101 MHz, THF-*d*_8_): δ 199.9 (dd, ^2^*J*_CP_ = 8 Hz, ^2^*J*_CP_ = 2 Hz, Ru*C*_NHC_), 191.0 (dd, ^2^*J*_CP_ = 86 Hz, ^2^*J*_CP_ = 16 Hz, Ru*C*_NHC_), 178.5 (d, ^2^*J*_CP_ = 17 Hz, Ru*C*_Ar_), 41.2 (dd, ^4^*J*_CP_ = 9 Hz, ^4^*J*_CP_ = 6 Hz, N*Me*), 38.7 (s, N*Me*), 35.9 (d, ^4^*J*_CP_ = 2 Hz, N*Me*), 35.7 (d, ^4^*J*_CP_ = 8 Hz, N*Me*), 10.0 (s, NC*Me*), 9.9 (s, NC*Me*), 9.7 (s, NC*Me*), −0.8 (d, ^3^*J*_CP_ = 9 Hz, Sn*Me*), −3.5 (dd, ^3^*J*_CP_ = 11 Hz, ^3^*J*_CP_ = 3 Hz, Sn*Me*). ^119^Sn{^1^H} NMR (187 MHz, THF-*d*_8_): δ 51.0 (dd, ^2^*J*_SnP_ = 1315 Hz, ^2^*J*_SnP_ = 243 Hz). Anal. Calcd. for C_52_H_58_N_4_P_2_RuSn·2C_6_H_6_ (1177.0): C 65.31, H 5.99, N, 4.76. Found: C 65.22, H 6.12, N, 4.91.

### [Ru(IMe_4_)(PPh_3_)(IMe_4_′)(PPh_2_C_6_H_4_SnMe_2_)] **11**

A THF-*d*_8_ solution of **10** (37 mg, 0.034 mmol) in a J. Young resealable NMR tube was heated at 60 °C. Monitoring by ^1^H and ^31^P NMR spectroscopy showed ca. 85% conversion through to **11** after 135 min. The reaction was pumped to dryness and the residue dissolved in a minimum amount of benzene and layered with hexane to give 21 mg of orange/yellow product comprised ca. 90% **11**, which was spectroscopically characterized. Selected ^1^H NMR (500 MHz, THF-*d*_8_): δ 3.22 (s, 3H, N*Me*), 2.98 (s, 3H, N*Me*), 2.77 (s, 3H, N*Me*), 2.42 (dd, *J*_HH_ = 3.3 Hz (second *J* coupling obscured by overlap of signal with N-*Me* of side product), 1H, NC*H*H), 2.22 (dd, *J* = 7.4 Hz, *J* = 3.3 Hz, 1H, NCH*H*), 1.75 (s, 3H, NC*Me*), 1.73 (s, 3H, NC*Me*), 1.47 (s, 3H, NC*Me*), 0.23 (s (^2^*J*_HSn_ = 18 Hz), 3H, Sn*Me*), −0.02 (s (^2^*J*_HSn_ = 23 Hz), 3H, Sn*Me*). ^31^P{^1^H} NMR (202 MHz, THF-*d*_8_): δ 81.4 (d, ^2^*J*_PP_ = 294 Hz (^2^*J*_PSn_ = 204 Hz)), 54.9 (d, ^2^*J*_PP_ = 294 Hz (^2^*J*_PSn_ = 218 Hz)). Selected ^13^C{^1^H} NMR (101 MHz, THF-*d*_8_): δ 192.4 (t, ^2^*J*_CP_ = 13 Hz, Ru*C*_NHC_), 169.3 (dd, ^1^*J*_CP_ = 64 Hz, ^3^*J*_CP_ = 3 Hz, RuPPh_2_*C*), 163.1 (t, ^2^*J*_CP_ = 14 Hz, Ru*C*_NHC_), 38.1 (s, N*Me*), 36.8 (s, N*Me*), 32.8 (s, N*Me*), 21.9 (t, ^2^*J*_CP_ = 9 Hz, Ru*C*H_2_), 10.1 (s, NC*Me*), 9.8 (s, NC*Me*), 8.8 (s, NC*Me*), 6.1 (s, NC*Me*), 2.6 (s (^1^*J*_CSn_ = 46 Hz), Sn*Me*), 1.0 (s, Sn*Me*).

### [Ru(IMe_4_)_2_(IMe_4_′)(PPh_2_C_6_H_4_SnMe_2_)] **12** and [Ru(PPh_3_)(IMe_4_′-SnMe_2_)(C_6_H_4_PPh_2_)] **13**

A J. Young resealable NMR tube containing a C_6_D_5_CD_3_ (0.5 mL) solution of **9** (40 mg, 0.034 mmol) was heated at 120 °C and conversion to **12** and **13** monitored by ^1^H and ^31^P{^1^H} NMR spectroscopy. The reaction was stopped after 1 h, concentrated and layered with hexane to afford 15 mg of a mixture of yellow (**12**) and purple (**13**) crystals. These were separated manually to allow NMR characterization and to isolate single crystals suitable for X-ray crystallography. Selected ^1^H NMR data for **12**. (500 MHz, THF-*d*_8_): δ 3.89 (s, 3H, N*Me*), 3.37 (s, 3H, N*Me*), 3.36 (s, 3H, N*Me*), 2.84 (s, 3H, N*Me*), 2.73 (s, 3H, N*Me*), 2.09 (s, 3H, NC*Me*), 2.05 (s, 3H, NC*Me*), 1.97 (s, 3H, NC*Me*), 1.84 (s, 3H, NC*Me*), 1.80 (s, 3H, NC*Me*), 1.16 (s, 3H, NC*Me*), 0.42 (s (^2^*J*_HSn_ = 12 Hz), 3H, Sn*Me*), 0.33 (s (^2^*J*_HSn_ = 14 Hz), 3H, Sn*Me*). ^31^P{^1^H} NMR (162 MHz, THF-*d*_8_): δ 76.5 (s). Selected ^13^C{^1^H} NMR (101 MHz, THF-*d*_8_): δ 202.8 (d, ^2^*J*_CP_ = 11 Hz, Ru*C*_NHC_), 198.6 (d, ^2^*J*_CP_ = 116 Hz, Ru*C*_NHC_), 170. 0 (d, ^2^*J*_CP_ = 18 Hz, Ru*C*_NHC_), 168.9 (d, ^1^*J*_CP_ = 78 Hz, PC_6_H_4_Sn), 39.3 (s, N*Me*), 37.3 (d, ^4^*J*_CP_ = 4 Hz, N*Me*), 36.1 (s, N*Me*), 34.2 (s, N*Me*), 32.4 (s, N*Me*), 23.2 (d, ^2^*J*_CP_ = 6 Hz, Ru*C*H_2_), 10.4 (s, NC*Me*), 10.1 (s, NC*Me*), 9.9 (s, NC*Me*), 9.8 (s, NC*Me*), 6.6 (s, NC*Me*), −1.5 (d, ^3^*J*_CP_ = 5 Hz, Sn*Me*), −1.7 (s, Sn*Me*). ^119^Sn{^1^H} NMR (187 MHz, THF-*d*_8_): δ 63.6 (d, ^2^*J*_SnP_ = 195 Hz). Selected NMR data for **13**. ^1^H NMR (400 MHz, THF-*d*_8_): δ 7.61–7.53 (m, 2H, Ar), 7.34–7.24 (m, 5H, Ar), 7.22–7.10 (m, 11H, Ar), 7.08–7.00 (m, 6H, Ar), 6.97–6.86 (m, 2H, Ar), 6.84–6.74 (m 2H, Ar), 6.51 (m, 1H, Ar), 2.70 (d, ^2^*J*_HH_ = 11.2 Hz (^2^*J*_HSn_ = 32 Hz), 1H, NC*H*H), 2.55 (s, 3H, N*Me*), 2.18 (d, ^2^*J*_HH_ = 11.2 Hz (^2^*J*_HSn_ = 14 Hz), 1H, NC*H*H), 2.12 (s, 3H, N*Me*), 2.01 (s, 3H, N*Me*), −0.20 (s (^2^*J*_HSn_ = 41 Hz), 3H, Sn*Me*), −0.59 (s (^2^*J*_HSn_ = 44 Hz), 3H, Sn*Me*). ^31^P{^1^H} NMR (162 MHz, THF-*d*_8_): δ 48.5 (d, ^2^*J*_PP_ = 21 Hz (^2^*J*_PSn_ = 145 Hz)), −22.6 (d, ^2^*J*_PP_ = 21 Hz (^2^*J*_PSn_ = 124 Hz)). ^13^C{^1^H} NMR (101 MHz, THF-*d*_8_): δ 196.3 (dd, ^2^*J*_CP_ = 79 Hz, ^2^*J*_CP_ = 7 Hz, Ru*C*_NHC_), 183.5 (dd, ^2^*J*_CP_ = 66 Hz, ^2^*J*_CP_ = 5 Hz, RuC_Ar_), 158.3 (dd, ^1^*J*_CP_ = 41 Hz, ^3^*J*_CP_ = 3 Hz, PAr), 142.5 (d, ^1^*J*_CP_ = 25 Hz, P-*C*_ipso_), 140.9 (d, ^1^*J*_CP_ = 25 Hz, P-*C*_ipso_), 139.5 (d, *J*_CP_ = 14 Hz, PAr), 137.5 (dd, *J*_CP_ = 14 Hz, *J*_CP_ = 3 Hz, PAr), 134.5 (d, *J*_CP_ = 13 Hz, PAr), 134.2 (d, *J*_CP_ = 12 Hz, PAr), 133.5 (d, *J*_CP_ = 11 Hz, PAr), 129.4 (s, PAr), 129.0 (s, PAr), 128.9 (d, *J*_CP_ = 14 Hz, PAr), 128.6 (s, PAr), 128.5 (d, *J*_CP_ = 8 Hz, PAr), 127.1 (s, N*C*Me), 125.7 (d, *J*_CP_ = 8 Hz, PAr), 124.0 (s, N*C*Me), 123.5 (d, *J*_CP_ = 8 Hz, PAr) 34.6 (d, ^3^*J*_CP_ = 6 Hz, N*C*H_2_), 38.4 (s, N*Me*), 34.5 (s, N*Me*), 10.7 (s, NC*Me*), 9.0 (s, NC*Me*), −5.4 (d, ^3^*J*_CP_ = 4 Hz, Sn*Me*), −6.4 (d, ^3^*J*_CP_ = 3 Hz, Sn*Me*). ^119^Sn{^1^H} NMR (187 MHz, THF-*d*_8_): δ 41.5 (dd, ^2^*J*_SnP_ = 145 Hz, ^2^*J*_SnP_ = 128 Hz).

### X-ray Crystallography

Data for **5**, **9**, **10**, and **13** were collected on an Agilent Xcalibur diffractometer (using a Mo Kα radiation) while the **6**, **11**, and **12** data sets were obtained using an Agilent SuperNova instrument and a Cu Kα source (Table S1). All experiments were conducted at 150 K, solved by employing either the solution program native to Olex2^47^ or SHELXT.^48^ Refinements were conducted using SHELXL^49^ via the Olex2 interface. Convergence of the models was largely unremarkable and only exceptional points of note will be outlined herein. In particular, the asymmetric unit in **5** was seen to contain one molecule of the organometallic complex and two molecules of THF. The hydride ligand in the main feature was located and refined without restraints, while C58 from the ligated THF was modeled to take account of 55:45 disorder. One of the guest THF molecules also resolved satisfactorily into two disordered components (60:40 ratio) with the inclusion of some distance and ADP restraints in the final least-squares. The second solvent moiety was readily identifiable as a THF, but disorder was messy, and it prevailed beyond two fractions. As such, this was ultimately treated using the solvent mask algorithm in Olex2, and an allowance for the same was made in the formula as presented.

In **10**, the asymmetric unit was noted to comprise one molecule of the Sn–Ru complex, one full-occupancy molecule of benzene and another benzene moiety which was modeled to take account of 72:28 disorder. Each component of the latter was treated as a rigid hexagon in the refinement. The highest residual electron density peaks in the difference Fourier map are at chemically insignificant distances from atoms in the main feature. Indeed, they may point toward some very minor disorder, at a level which negates modeling.

The hydrogen atoms attached to C1 in **11** were located and refined subject to being a common distance from the parent atom. The highest residual electron density peak is located at a chemically insignificant distance from Sn1. One molecule of the organometallic complex and a region of solvent correspond to the asymmetric unit in the structure of **12**. The hydrogen atoms attached to C7 were located and refined freely. Analysis of the electron density indicated 5% disorder of the tin center (at location Sn1a) and this was accounted for in the model. However, no effort was made to model the necessary 5% disorder of the phosphine ligand attached the main group metal, as it would be imprudent to invest in location of 5% disorder for first row elements with the expectation of credibility. The solvent moiety was very disordered and was ultimately treated using the solvent mask algorithm available in Olex2, with an allowance made for the presence of one molecule of toluene, per asymmetric unit, in the formula as presented.

In **13**, the hydrogen atoms attached to C6 were located and refined freely. The electron density indicated 9% disorder of the tin center (at location Sn1a) and this was accounted for in the model. However, (using similar rationale to that employed for **6**) no effort was made to model the necessary 9% disorder of the phosphine ligand attached the minor tin component. Distance and ADP restraints were included for the minor tin component.

### Computational Methodology

DFT calculations were run with Gaussian 16 (C.01).^50^ The Al, P, Ru and Sn centers were described with the Stuttgart RECPs and associated basis sets,^51^ and the 6-31G** basis set was used for all other atoms (BS1).^52^ A polarization function was also added to Al (ζ_d_ = 0.190), P (ζ_d_ = 0.387) and Sn (ζ_d_ = 0.180).^53^ Initial BP86 optimizations^54^ were performed using the ‘grid = ultrafine’ option, with all stationary points being fully characterized via analytical frequency calculations as minima. All energies were recomputed with a larger basis set (BS2) featuring 6-311++G** basis sets on all atoms, with the exception of Ru and Sn which employed the basis set aug-cc-pVTZ-PP. Corrections for the effect of solvent (benzene: ε = 2.2706; THF: ε = 7.4257) solvent were employed using the polarizable continuum model and BS1.^55^ Single-point dispersion corrections to the BP86 results employed Grimme’s D3 parameter set with Becke-Johnson damping as implemented in Gaussian.^56^ Natural Bonding Orbital (NBO 3.1)^57^ analyses were performed on the BP86/BS1 optimized geometries at the BP86/BS2 level.

## Supporting Information Available

Structures and Cartesian coordinates (XYZ)
NMR spectra of compounds **5**–**13**, Table S1, and computational details (PDF)

## Supplementary Material

om2c00344_si_001.xyz
