# Supplementary material for: Structure and Reactivity of [Ru–Al] and [Ru–Sn] Heterobimetallic PPh3-Based Complexes
Source: Organometallics. 2022 Sep 21;41(19):2716–30. doi: 10.1021/acs.organomet.2c00344 (PMC9554919; doi:10.1021/acs.organomet.2c00344)
Supplement: Supplementary file 2 — om2c00344_si_002.pdf [file om2c00344_si_002.pdf]

SUPPORTING INFORMATION

Structure and Reactivity of [Ru-Al] and [Ru-Sn] Heterobimetallic PPh_3-Based Complexes

Connie J. Isaac, Fedor M. Miloserdov, Anne-Frédérique Pécharman, John P. Lowe, Claire L. McMullin, and Michael K. Whittlesey

Department of Chemistry, University of Bath, Claverton Down, Bath BA2 7AY, UK

Contents

NMR spectra S-2

Table S1 S-61

Computational Details S-62

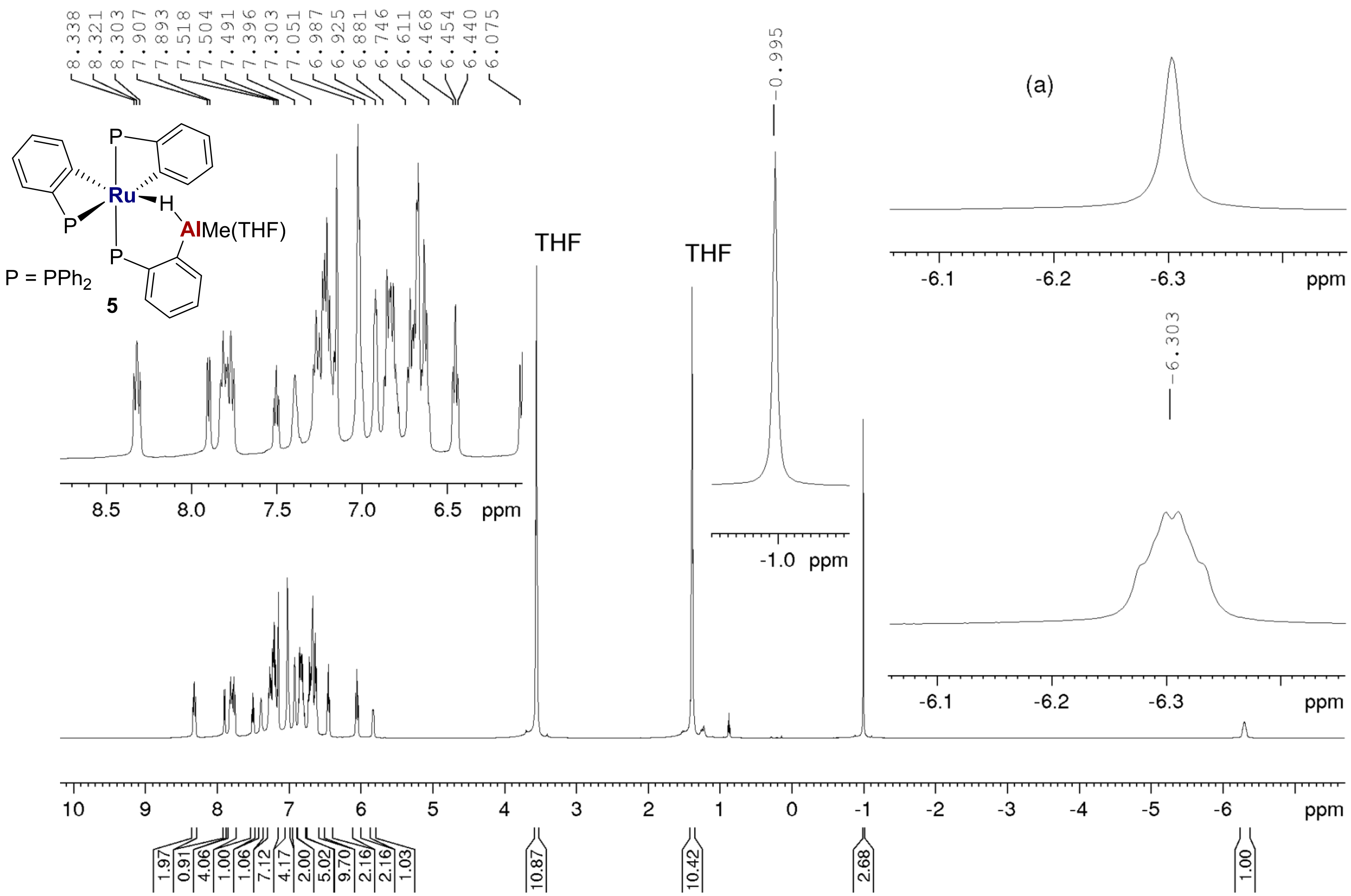

Figure S1. ^{1}H NMR spectrum (500 MHz, C_6D_6, 298 K) of $[Ru(C_6H_4PPh_2)_2\{PPh_2C_6H_4AlMe(THF)\}H]$ **5**, with expansions of aromatic, Al*Me* and Ru-*H*-Al regions. Inset (a) shows the latter signal in the $^{1}H\{^{31}P\}$ NMR spectrum (500 MHz, C_6D_6, 298 K).

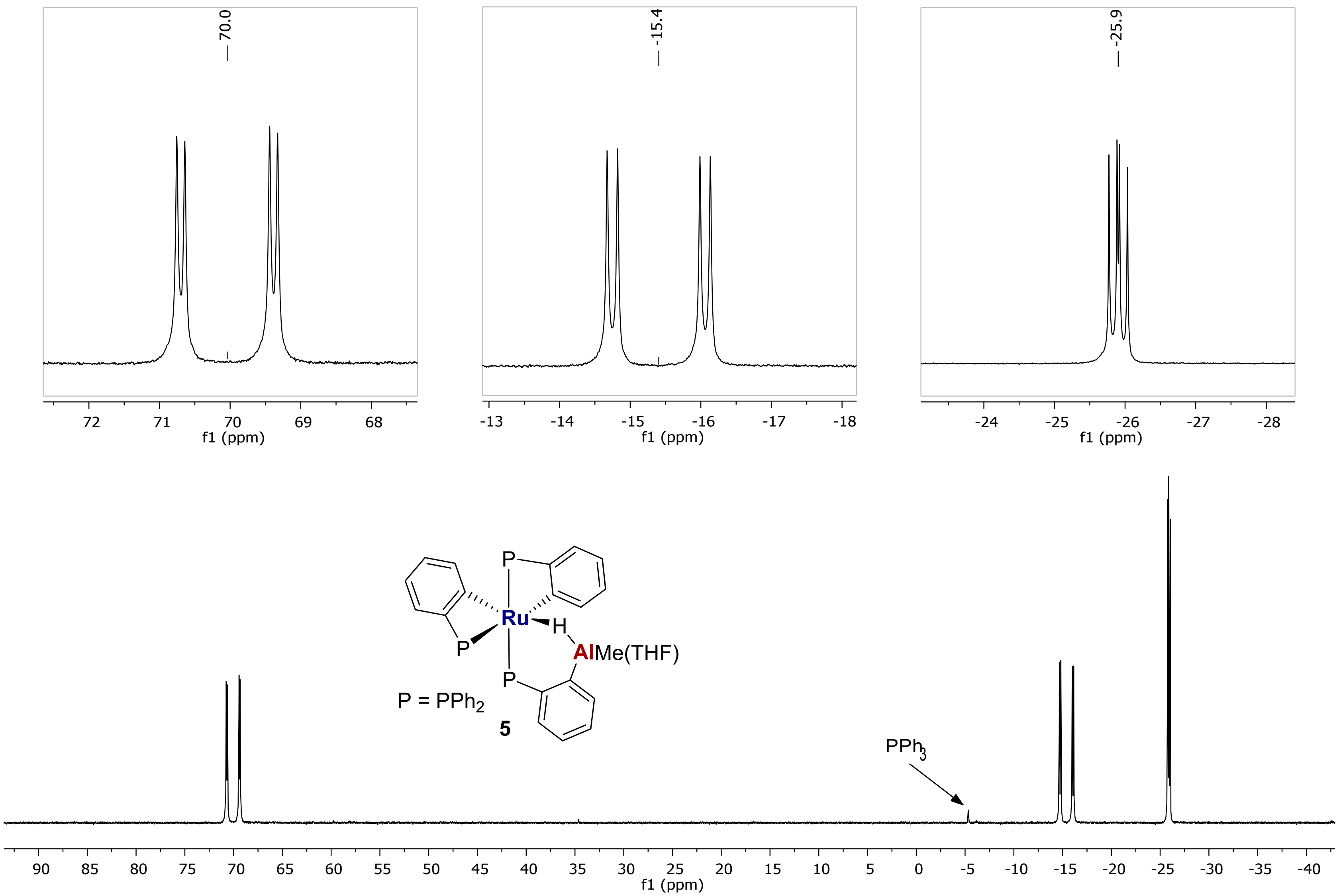

Figure S2. $^{31}P\{^1H\}$ NMR spectrum (202 MHz, C_6D_6, 298 K) of $[Ru(C_6H_4PPh_2)_2\{PPh_2C_6H_4AlMe(THF)\}H]$ **5**.

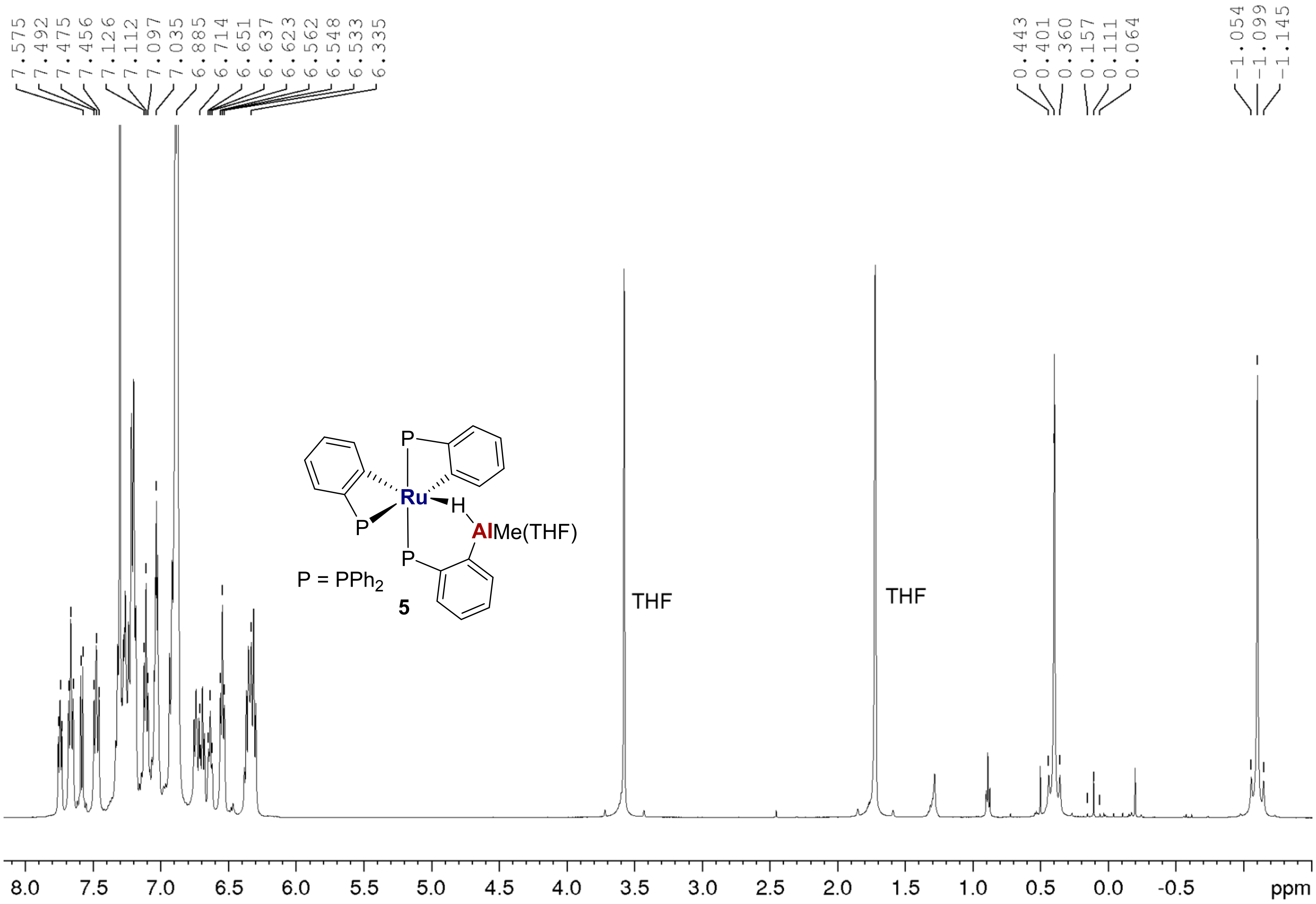

Figure S3. ^{1}H NMR spectrum (500 MHz, THF-d_8, 298 K) of [Ru(PPh_3)($C_6H_4PPh_2$){$PPh_2C_6H_4SnMe_2$}] **5**.

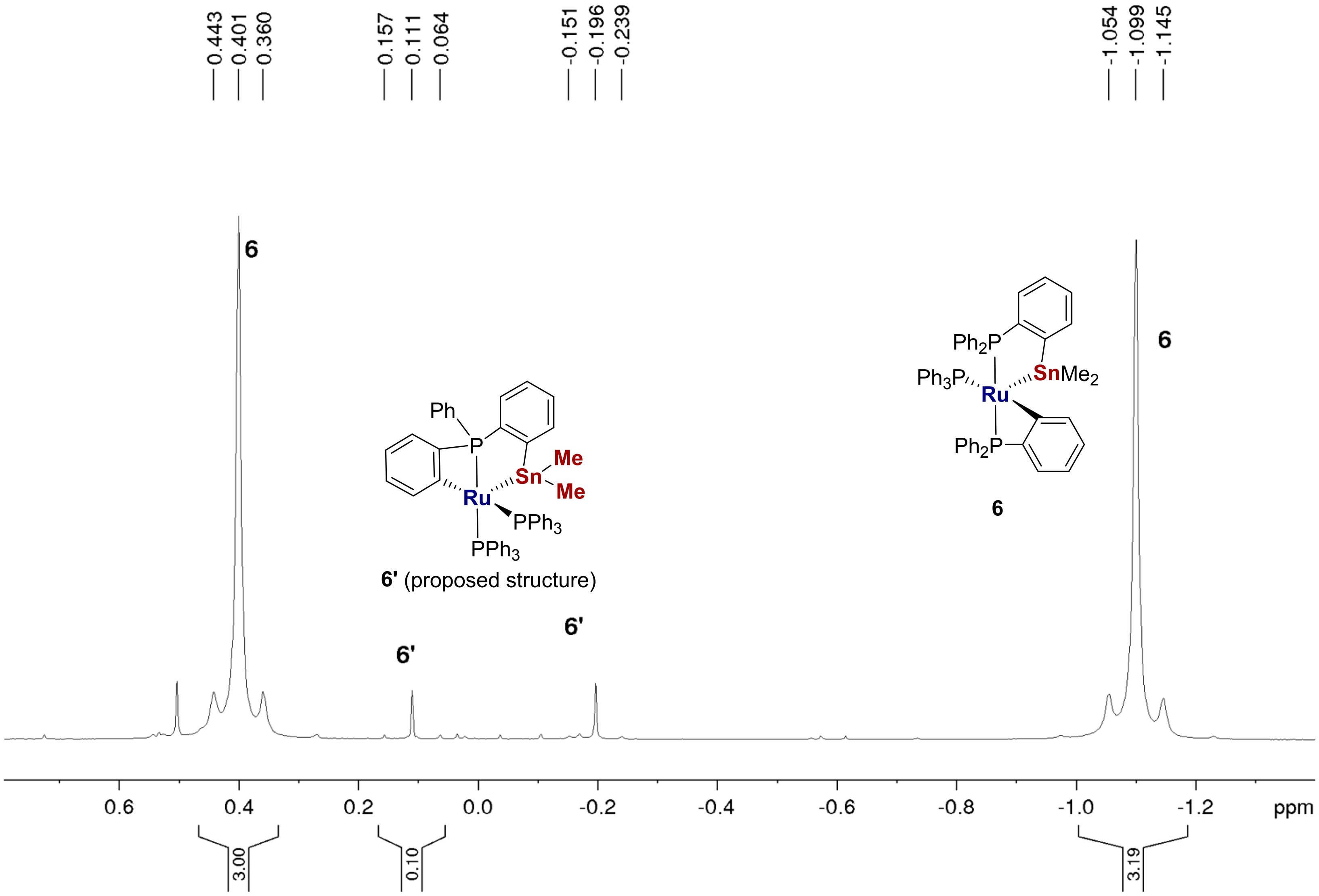

Figure S4. Partial ^{1}H NMR spectrum (500 MHz, THF-d_8, 298 K) of [Ru(PPh_3)($C_6H_4PPh_2$){$PPh_2C_6H_4SnMe_2$}] **6** showing Sn*Me* signals for **6** and proposed minor isomer **6'**.

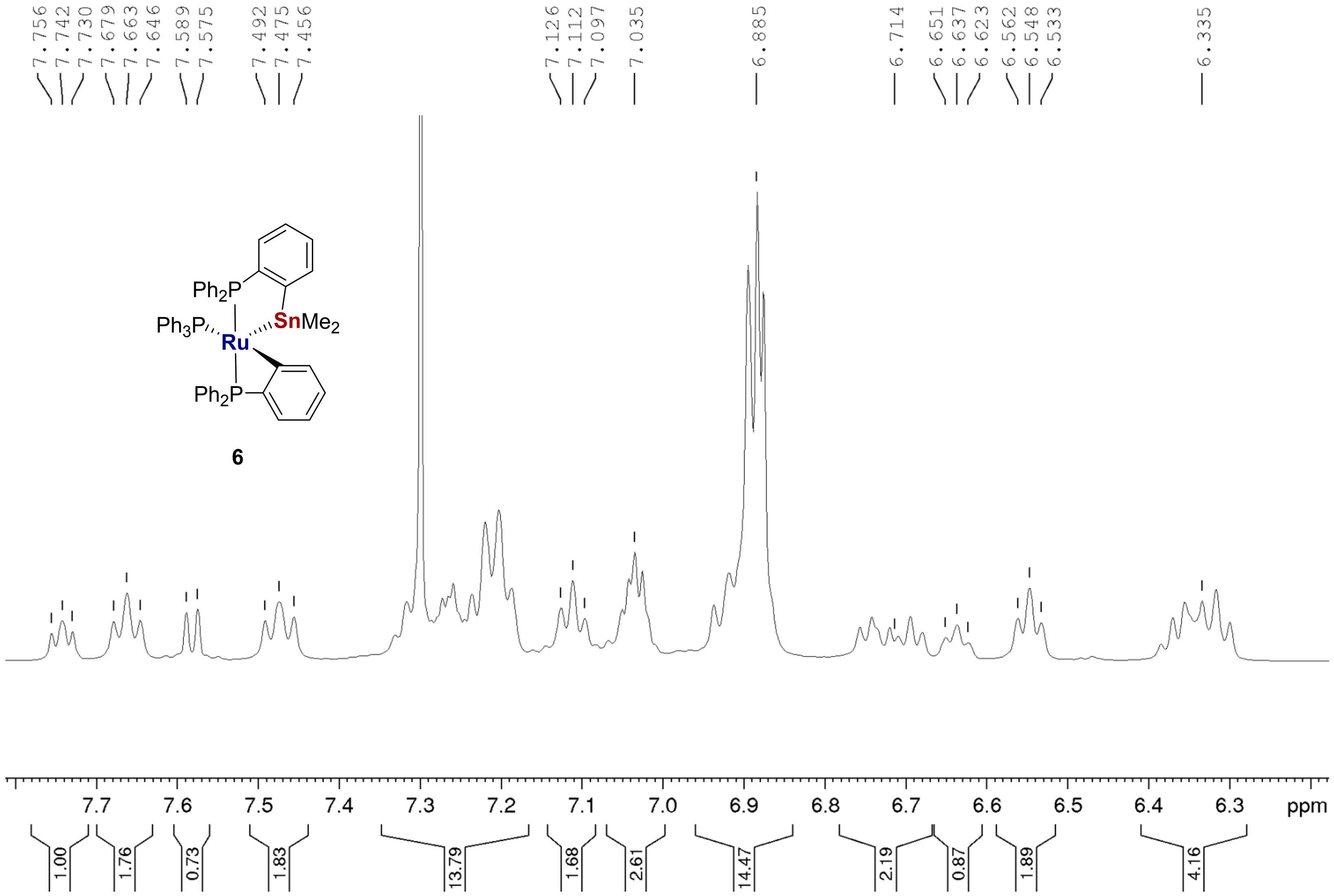

Figure S5. Aromatic region of the ^{1}H NMR spectrum (500 MHz, THF-d_8, 298 K) of $[Ru(PPh_3)(C_6H_4PPh_2)\{PPh_2C_6H_4SnMe_2\}]$ **6**.

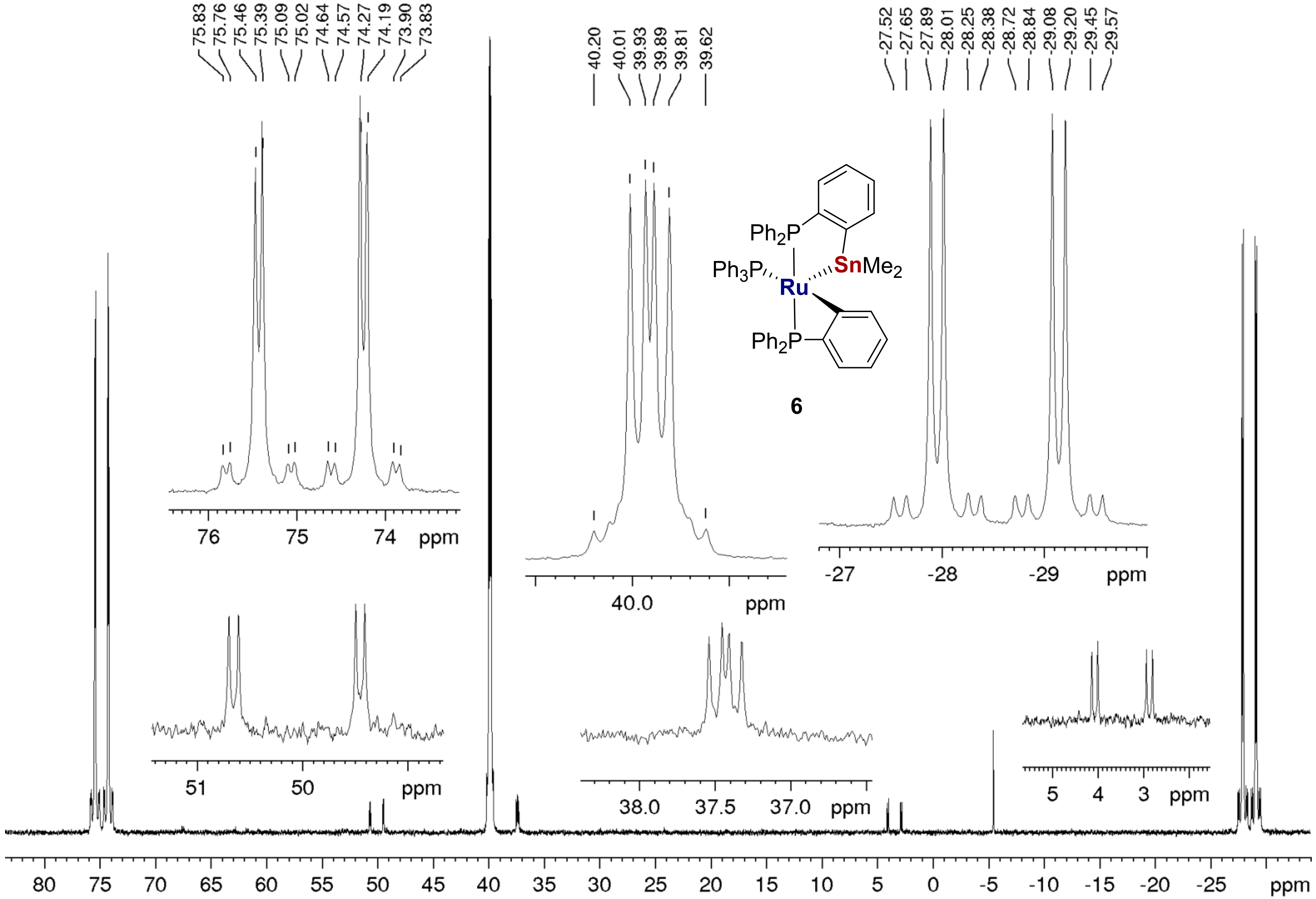

Figure S6. $^{31}P\{^1H\}$ NMR spectrum (202 MHz, THF-d_8, 298 K) showing resonances for major species $[Ru(PPh_3)(C_6H_4PPh_2)\{PPh_2C_6H_4SnMe_2\}]$ **6**. Smaller resonances arise from the proposed minor species **6′**.

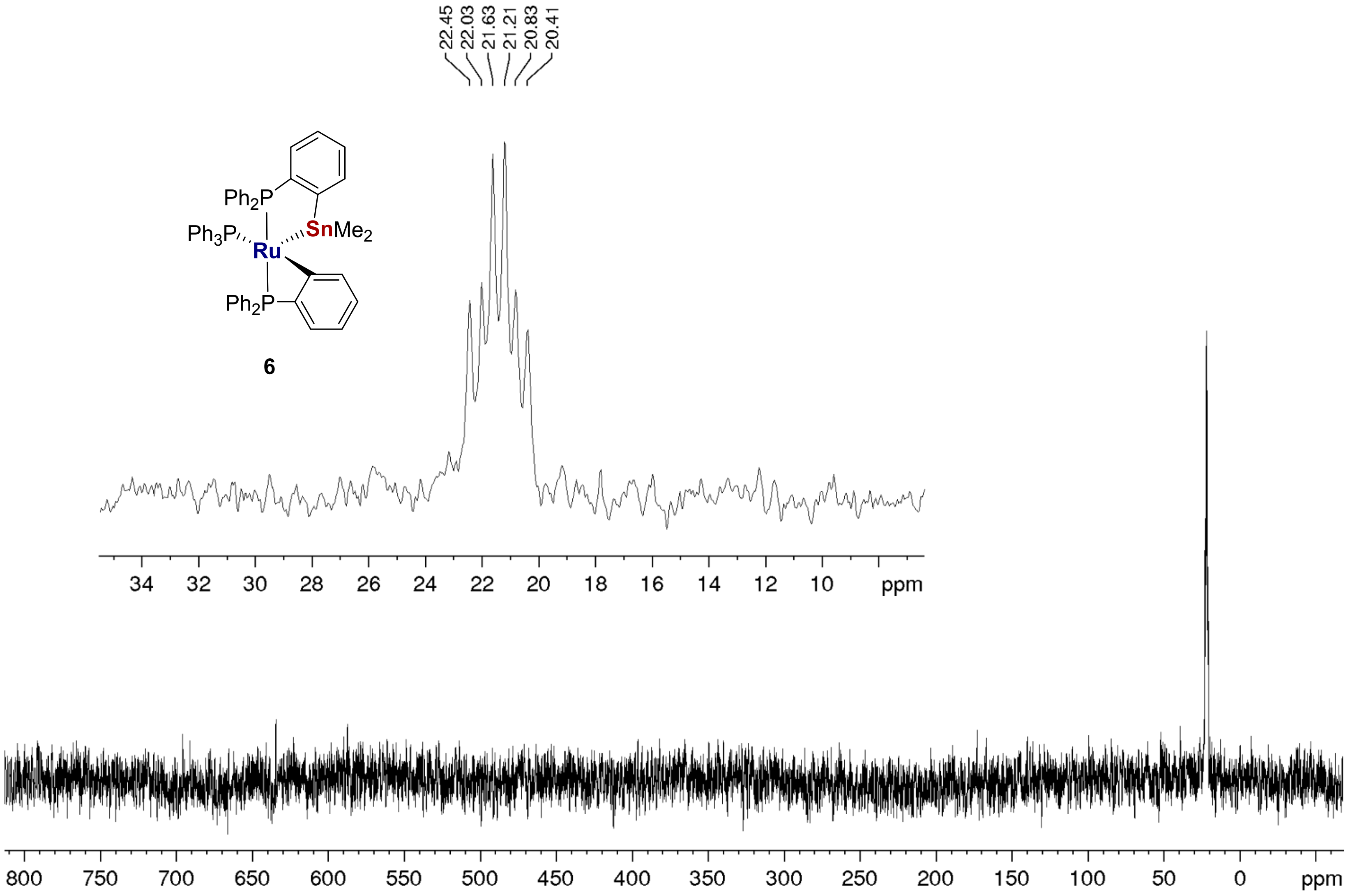

Figure S7. $^{119}Sn\{^1H\}$ NMR spectrum (187 MHz, THF-d_8, 298 K) of [Ru(PPh_3)($C_6H_4PPh_2$){$PPh_2C_6H_4SnMe_2$}] **6**.

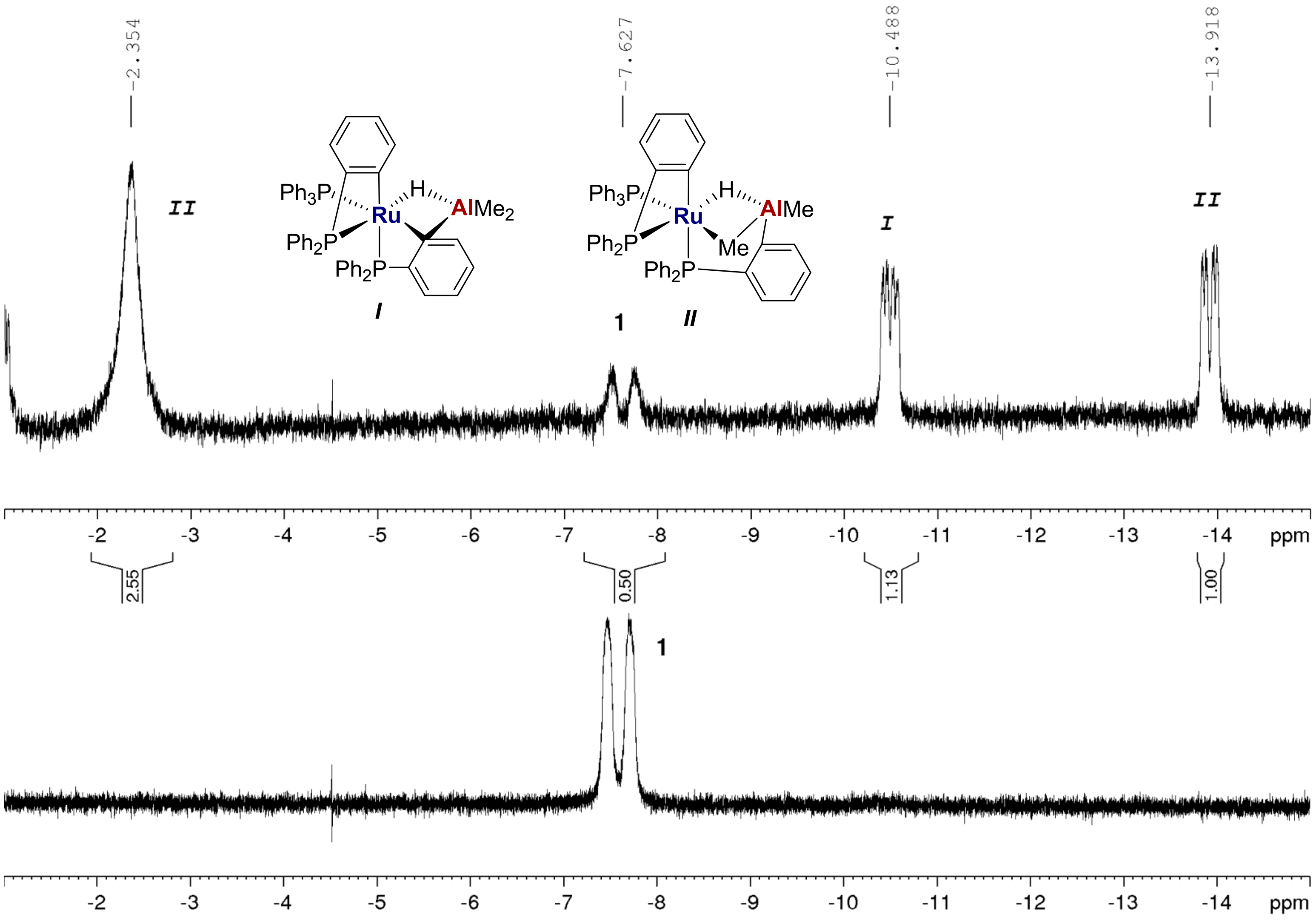

Figure S8. Low frequency region of the ^{1}H NMR spectrum (400 MHz, THF-d_8, 193 K) of (bottom) **1** and (top) intermediates ***I*** and ***II*** formed upon addition of Me_2AlCl (15 min at 193 K).

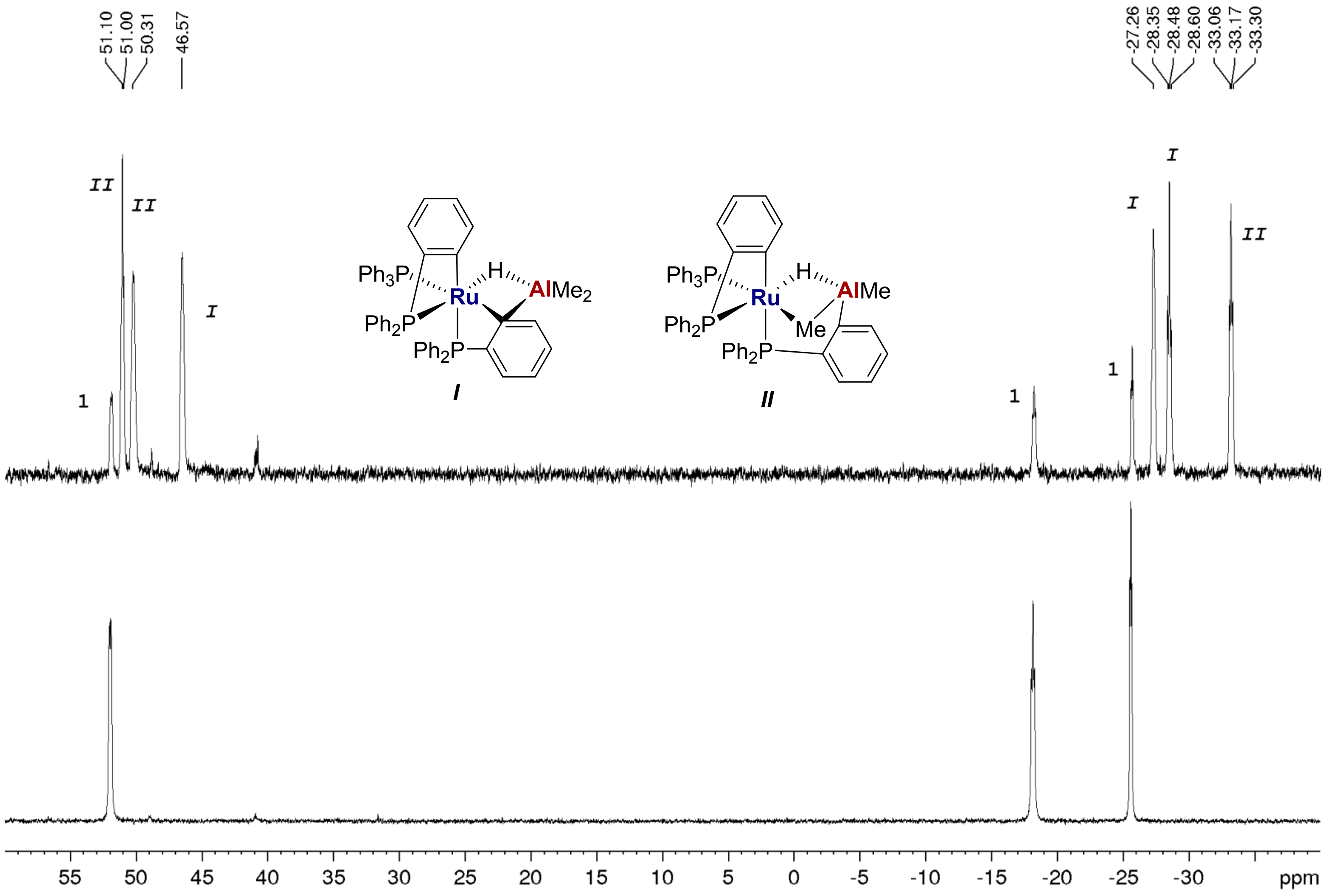

Figure S9. $^{31}P\{^{1}H\}$ NMR spectrum (162 MHz, THF-d_8, 193 K) of (bottom) **1** and (top) intermediates ***I*** and ***II*** formed upon addition of Me_2AlCl (15 min at 193 K).

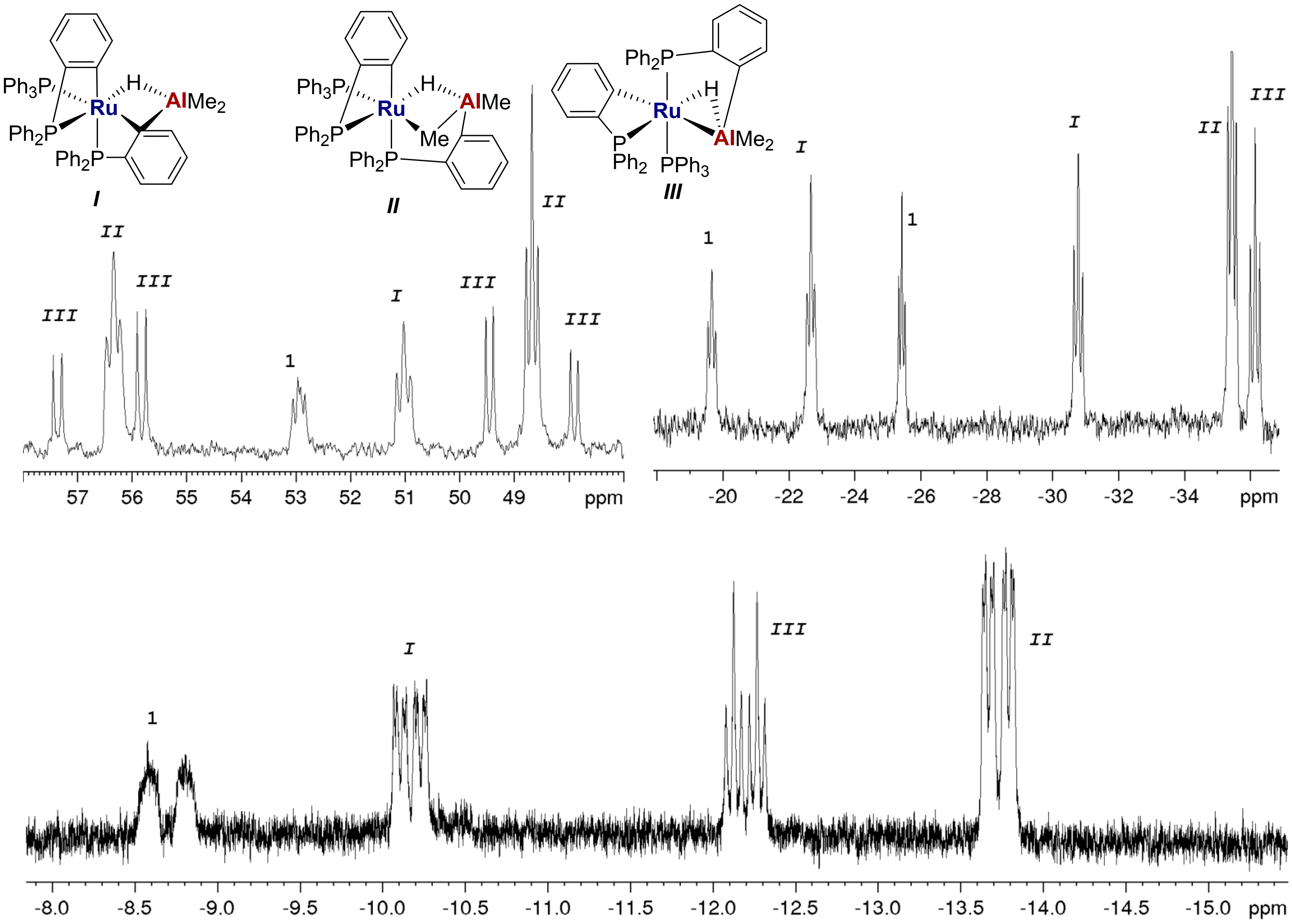

Figure S10. (Bottom) Hydride region of the ^{1}H NMR spectrum (400 MHz, THF-d_8) and (top) $^{31}P\{^{1}H\}$ NMR spectrum (162 MHz, THF-d_8) of the reaction of **1** and Me_2AlCl recorded after 1 h at 233 K showing the presence of intermediates ***I-III***.

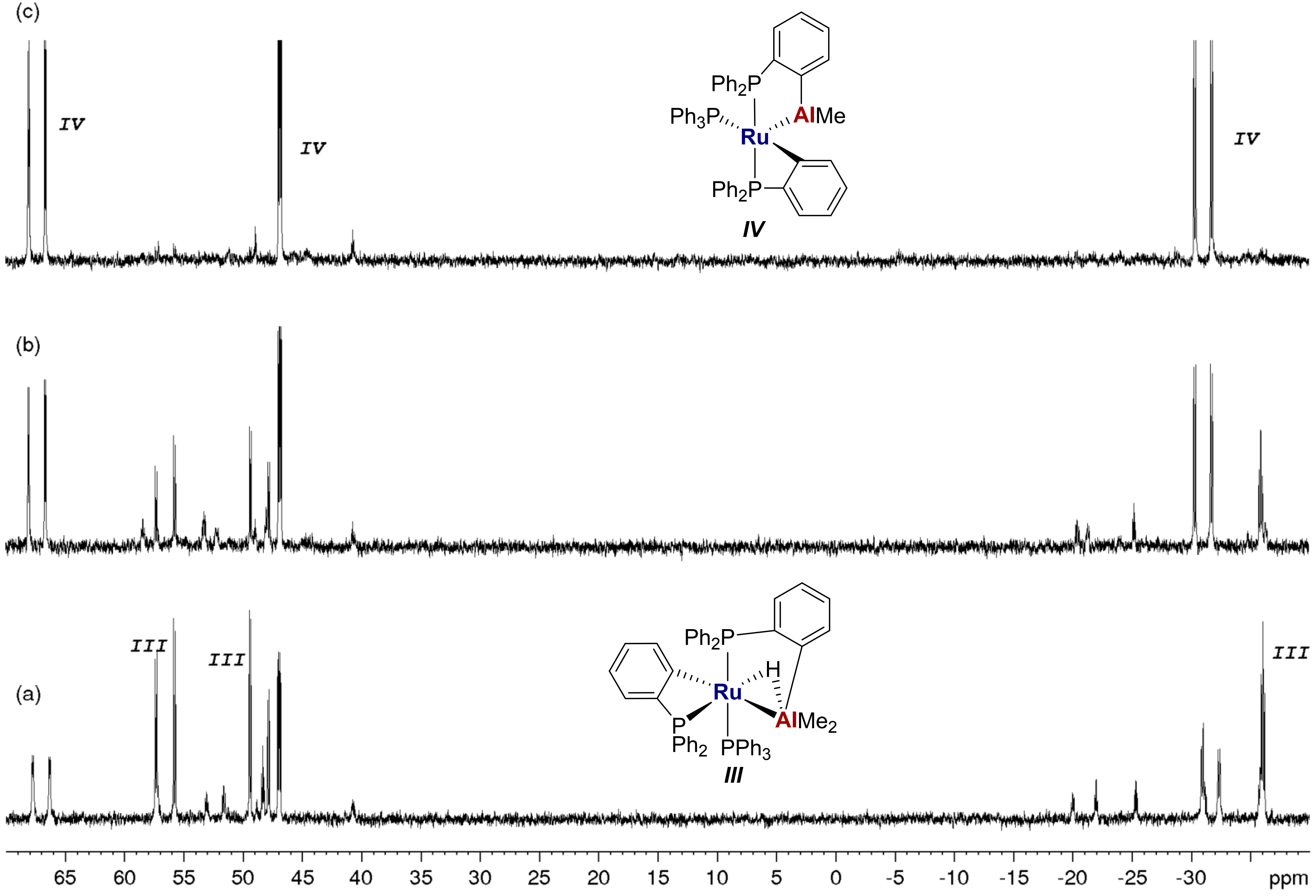

Figure S11. $^{31}P\{^1H\}$ NMR spectra (162 MHz, THF-d_8) of intermediates ***III*** and ***IV*** formed upon 193 K addition of Me_2AlCl to **1**. Spectra recorded (a) after leaving for 2 h at 253 K, (b) 15 min at 273 K and (c) 1 h at 273 K.

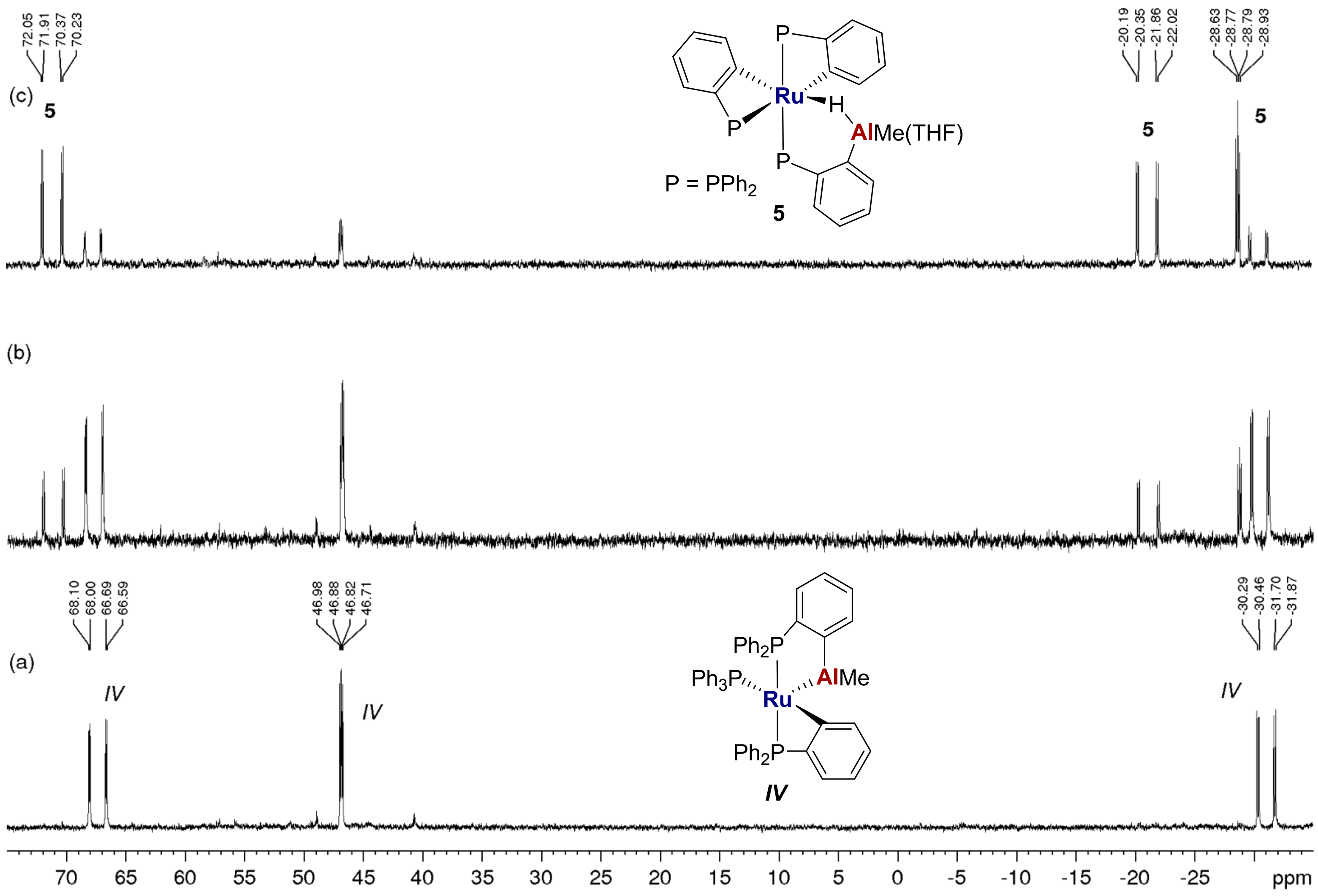

Figure S12. $^{31}P\{^{1}H\}$ NMR spectra (162 MHz, THF-d_8) of the 193 K reaction of Me_2AlCl with **1** showing the conversion of intermediate ***IV*** to **5**. Spectra recorded (a) at 273 K, (b) after 15 min at 298 K and (c) after 45 min at 298 K.

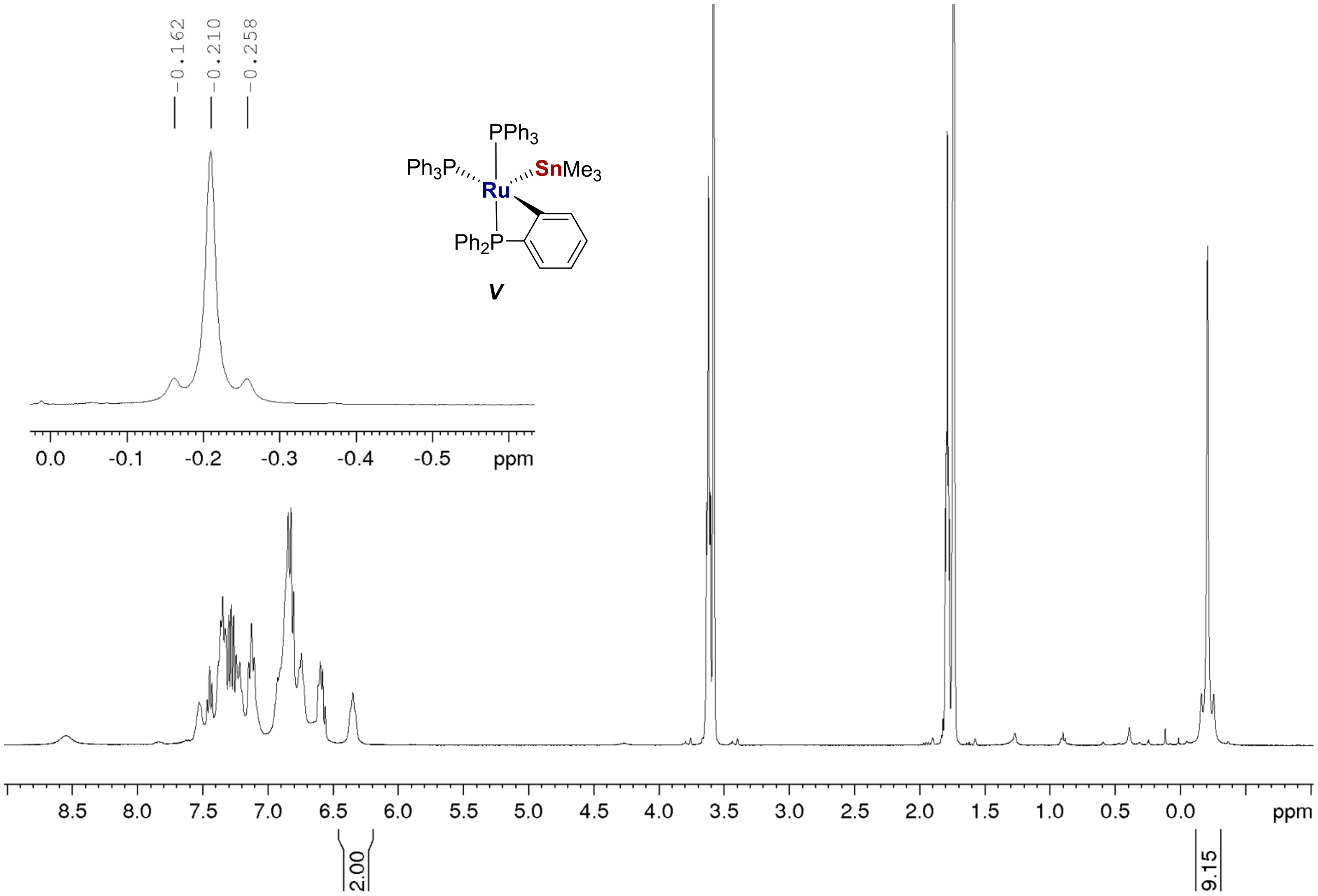

Figure S13. ^{1}H NMR spectrum (400 MHz, THF-d_8, 193 K) of the reaction of **1** with Me_3SnCl to give intermediate ***V***. Inset shows expansion of the SnMe_3 signal.

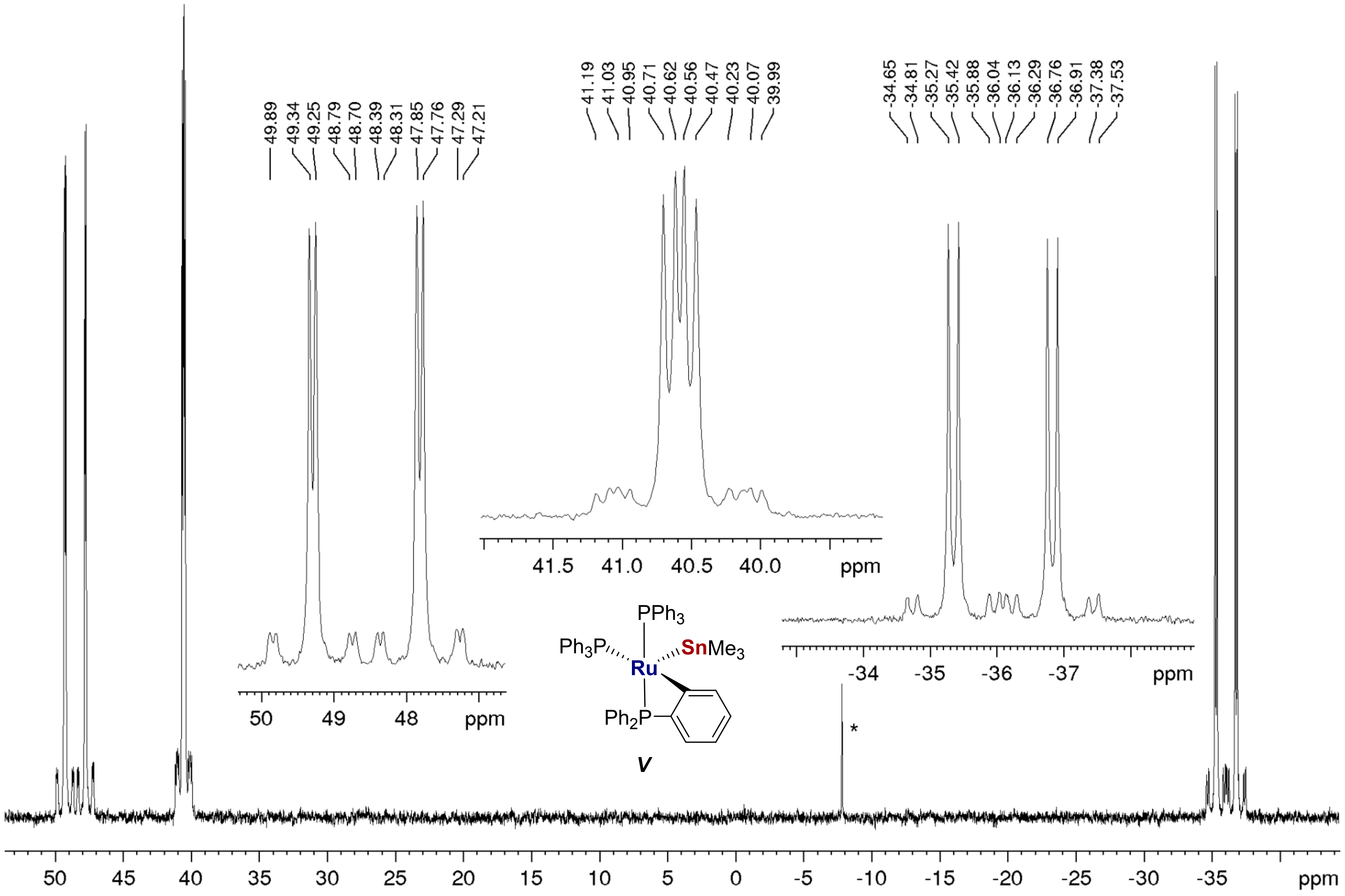
49.89
49.34
49.25
48.79
48.70
48.39
48.31
47.85
47.76
47.29
47.21
41.19
41.03
40.95
40.71
40.62
40.56
40.47
40.23
40.07
39.99
-34.65
-34.81
-35.27
-35.42
-35.88
-36.04
-36.13
-36.29
-36.76
-36.91
-37.38
-37.53
50
49
48
ppm
41.5
41.0
40.5
40.0
ppm
-34
-35
-36
-37
ppm
PPh3
Ph3P
SnMe3
Ru
Ph2P
V
*
50
45
40
35
30
25
20
15
10
5
0
-5
-10
-15
-20
-25
-30
-35
ppm

Figure S14. $^{31}P\{^1H\}$ NMR spectrum (162 MHz, THF-d_8, 193 K) of the reaction of **1** with Me_3SnCl to give intermediate ***V***. Insets show expansions of the three resonances. * = PPh_3.

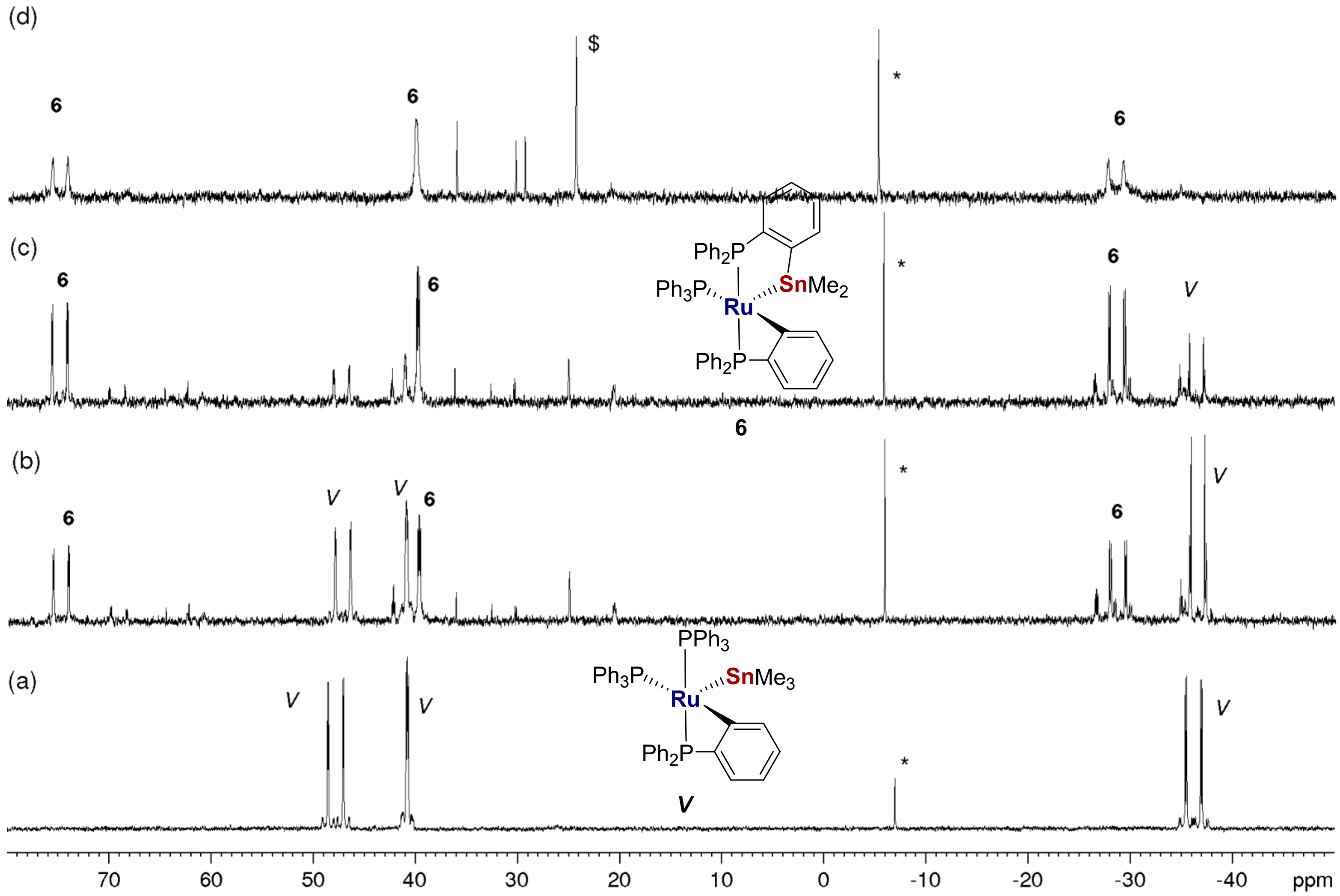

Figure S15. $^{31}P\{^1H\}$ NMR spectra (162 MHz, THF-d_8) of the reaction of **1** with Me_3SnCl at 193 K. (a) Spectrum (233 K) showing presence of ***V***, at 273 K for (b) 5 min and (c) 30 min with transformation of ***V*** to **6** and (d) at 298 K. * = PPh_3; $ = $OPPh_3$.

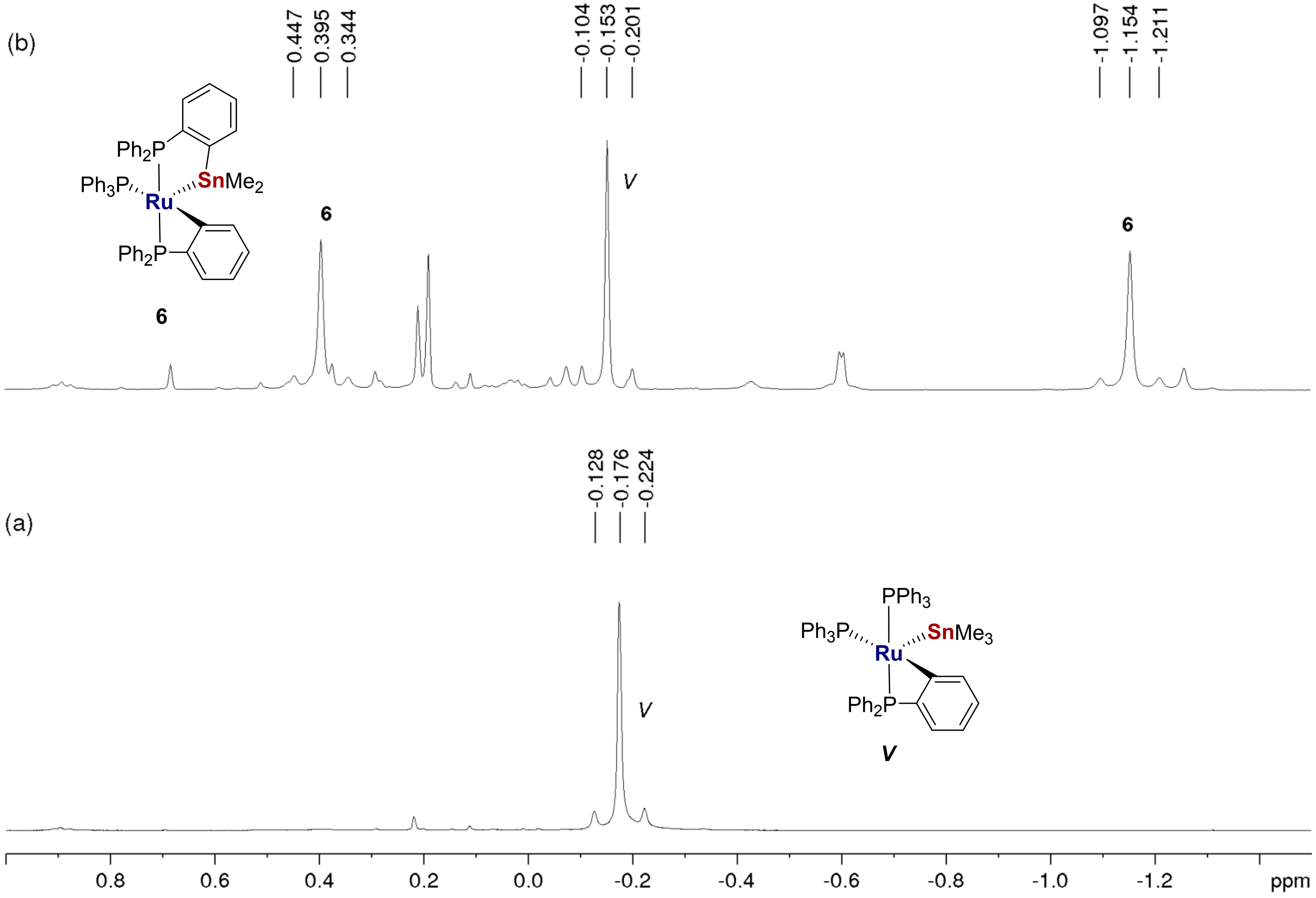

Figure S16. Partial ^{1}H NMR spectra (400 MHz, THF-d_8) of the reaction of **1** with Me_3SnCl at 193 K showing (a) SnMe_3 signal of ***V*** at 233 K and (b) appearance of the two resonances of the SnMe_2 group of **6** upon warming to 273 K.

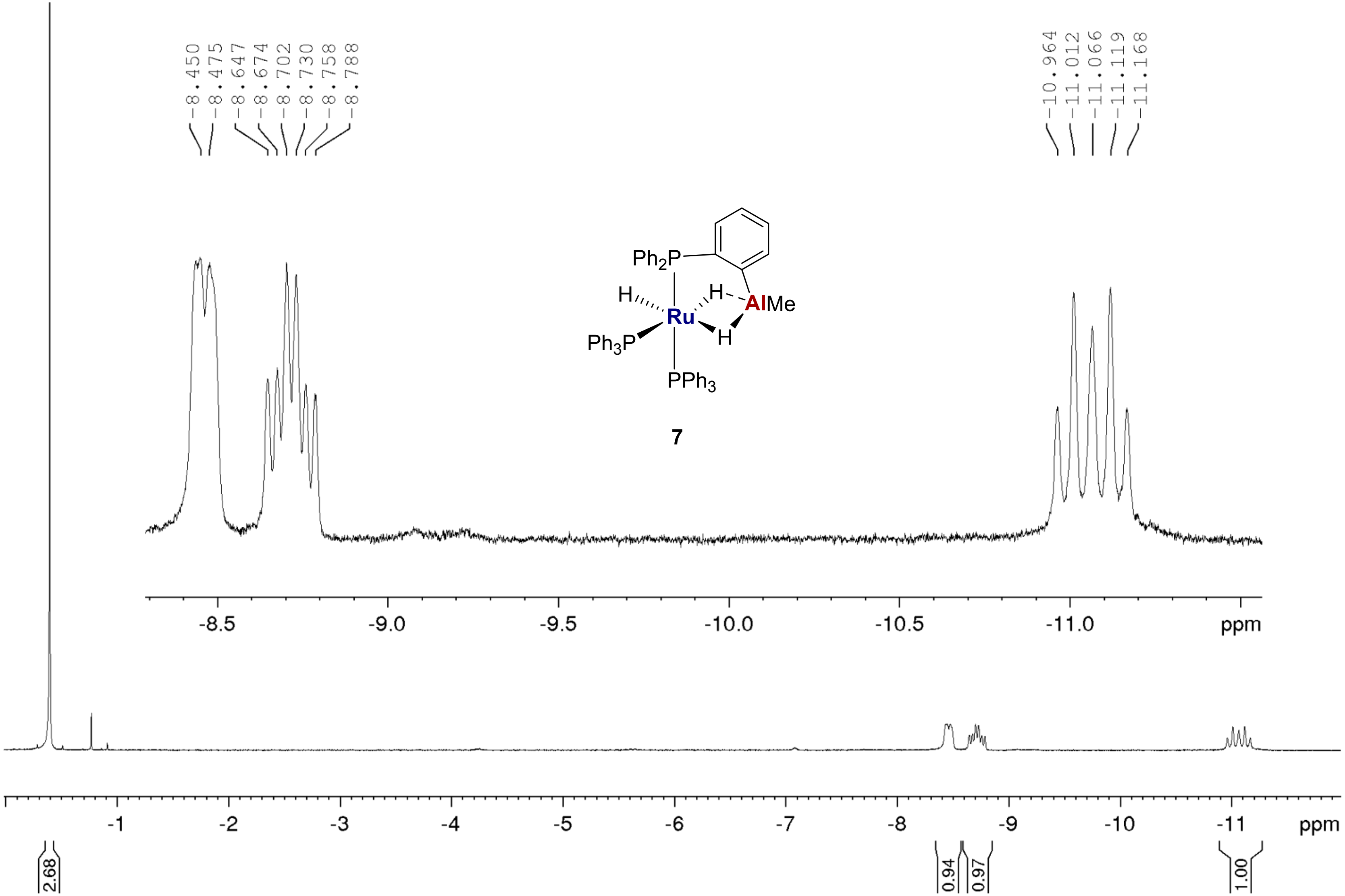

Figure S17. Low frequency region of the ¹H NMR spectrum (500 MHz, C_6D_6, 298 K) from the reaction of $[Ru(C_6H_4PPh_2)_2\{PPh_2C_6H_4AlMe(THF)\}H]$ **5** with H_2 at 60 °C to give $[Ru(PPh_3)_2\{PPh_2C_6H_4AlMe\}H_3]$ **7**, with expansion of the hydride signals.

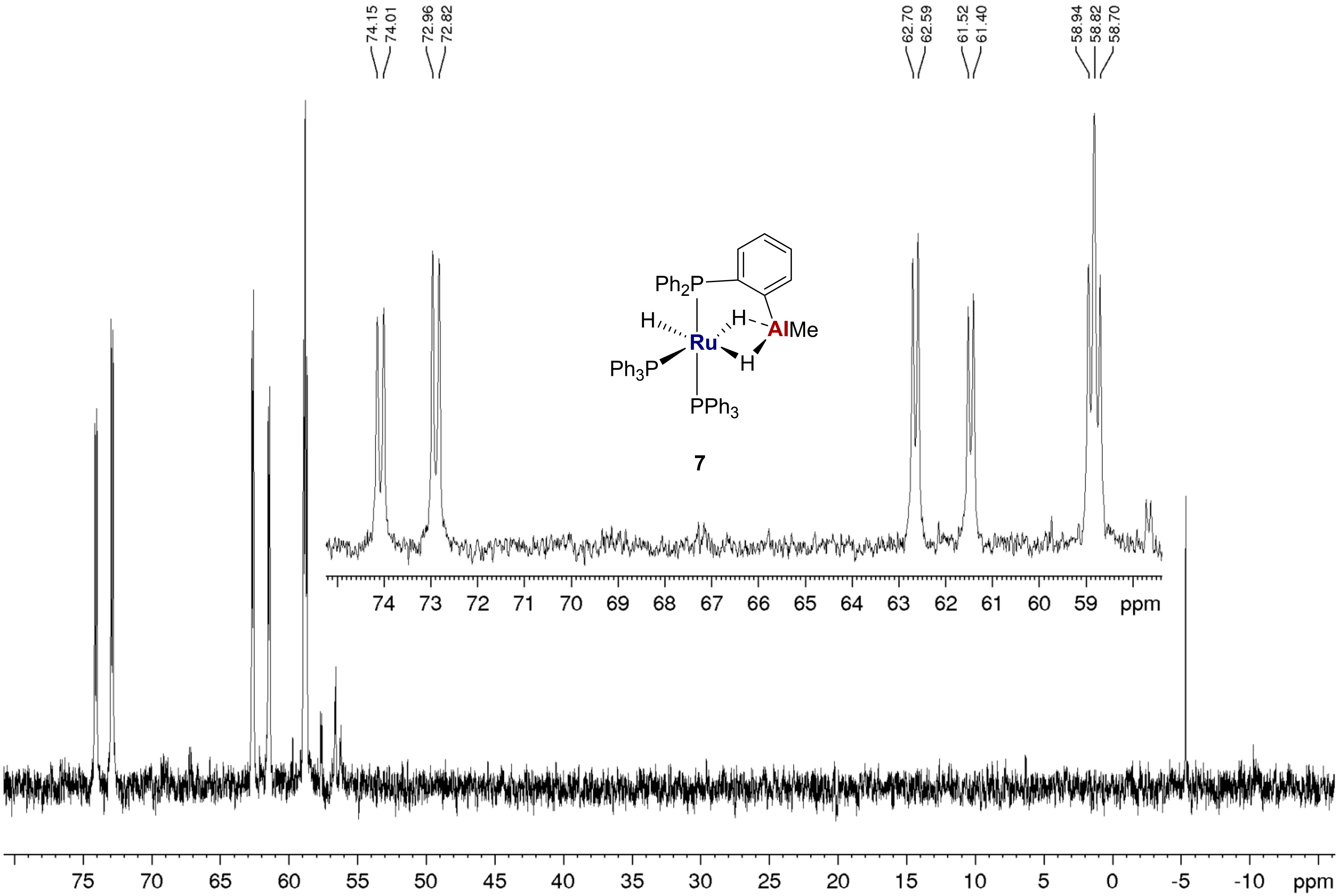

Figure S18. $^{31}P\{^1H\}$ NMR spectrum (202 MHz, C_6D_6, 298 K) from the reaction of [Ru($C_6H_4PPh_2$)$_2$\{$PPh_2C_6H_4$AlMe(THF)\}H] **5** with H_2 at 60 °C, with inset of the resonances assigned to the product [Ru(PPh_3)$_2$\{$PPh_2C_6H_4$AlMe\}H_3] **7**.

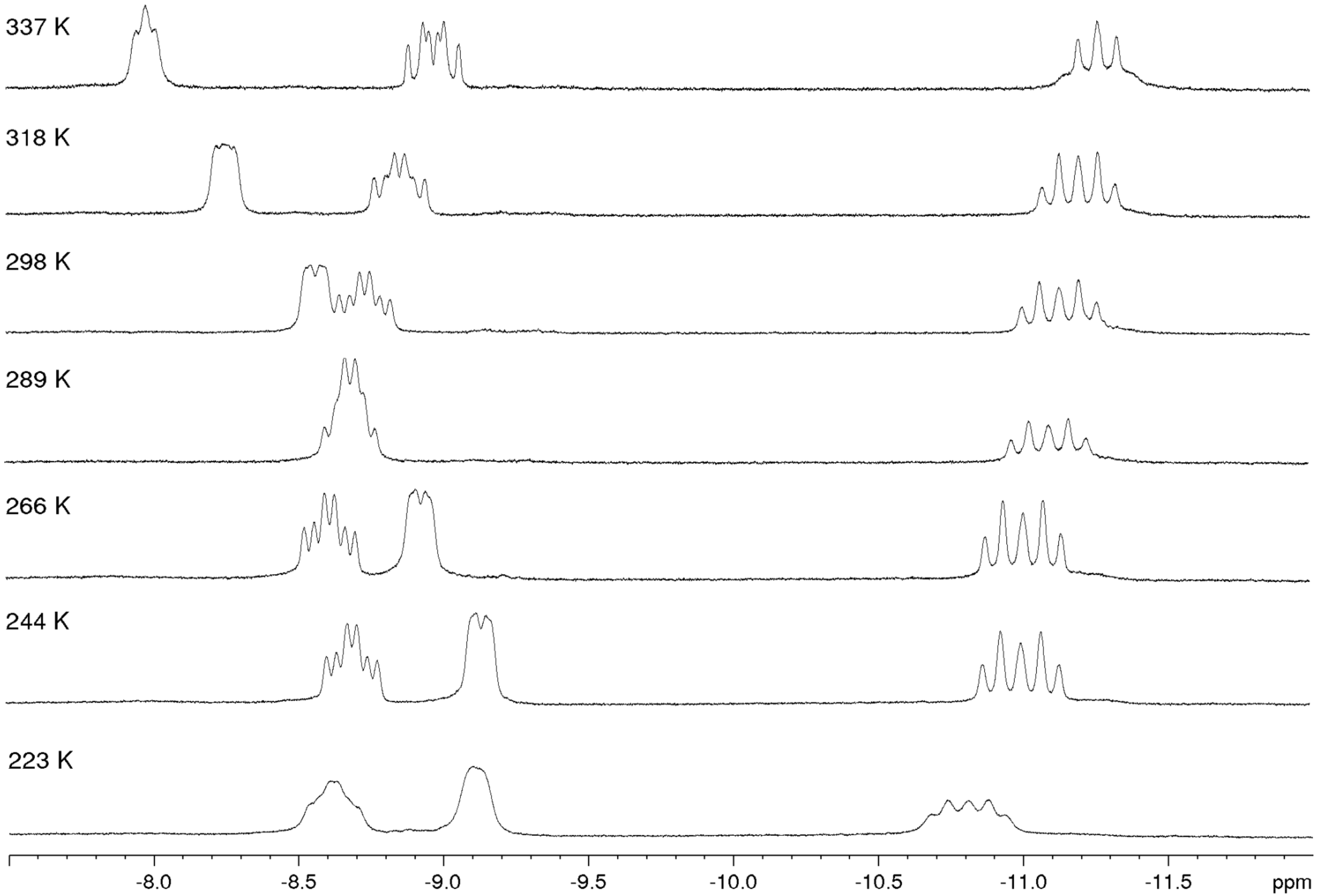

Figure S19. Hydride region of the variable temperature ^{1}H NMR spectra (400 MHz, $C_6D_5CD_3$) of $[Ru(PPh_3)_2\{PPh_2C_6H_4AlMe\}H_3]$ **7**.

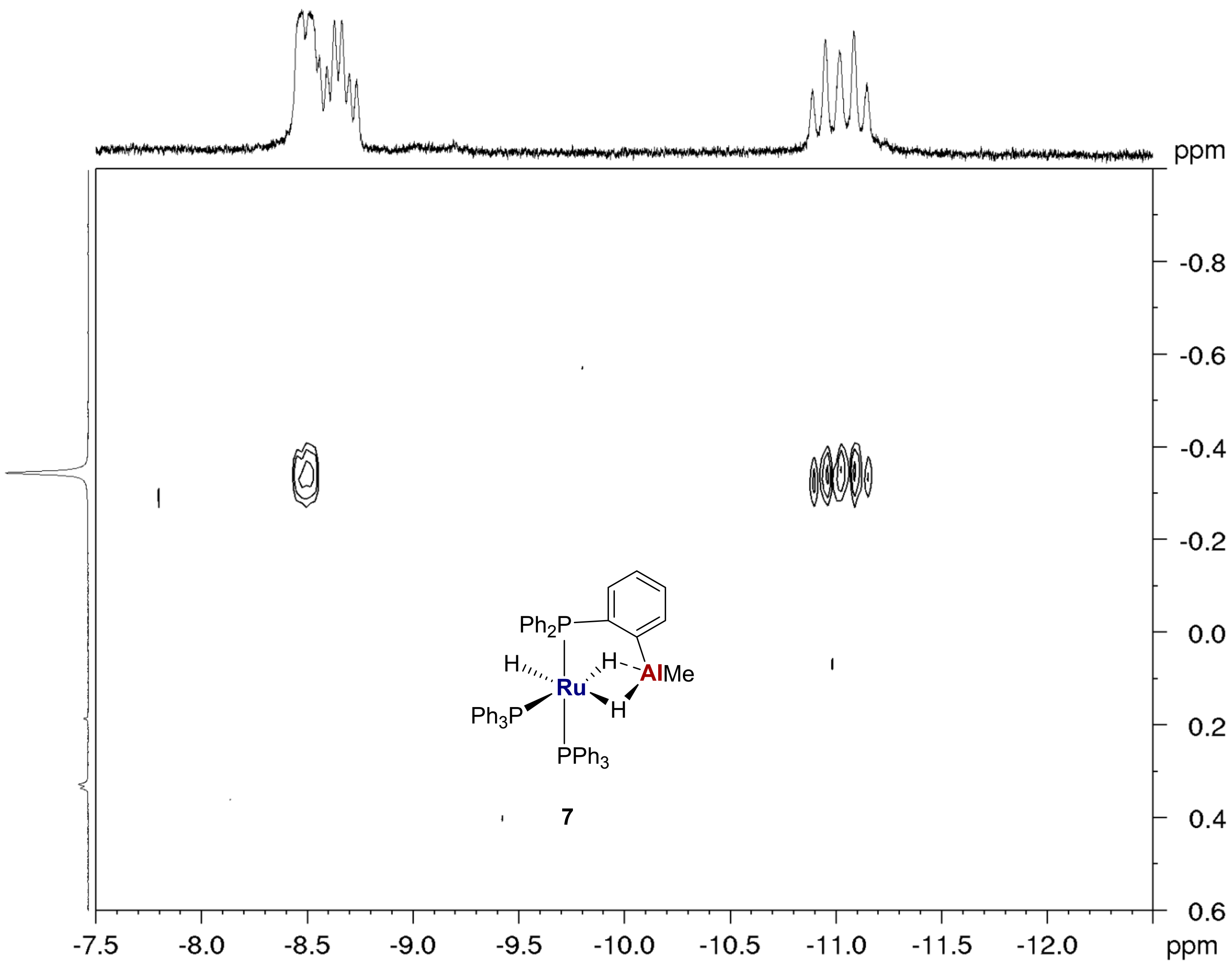

Figure S20. NOESY spectrum (400 MHz, C_6D_6) showing correlation between Al*Me* and two of the hydride resonances in $[Ru(PPh_3)_2\{PPh_2C_6H_4AlMe\}H_3]$ **7**.

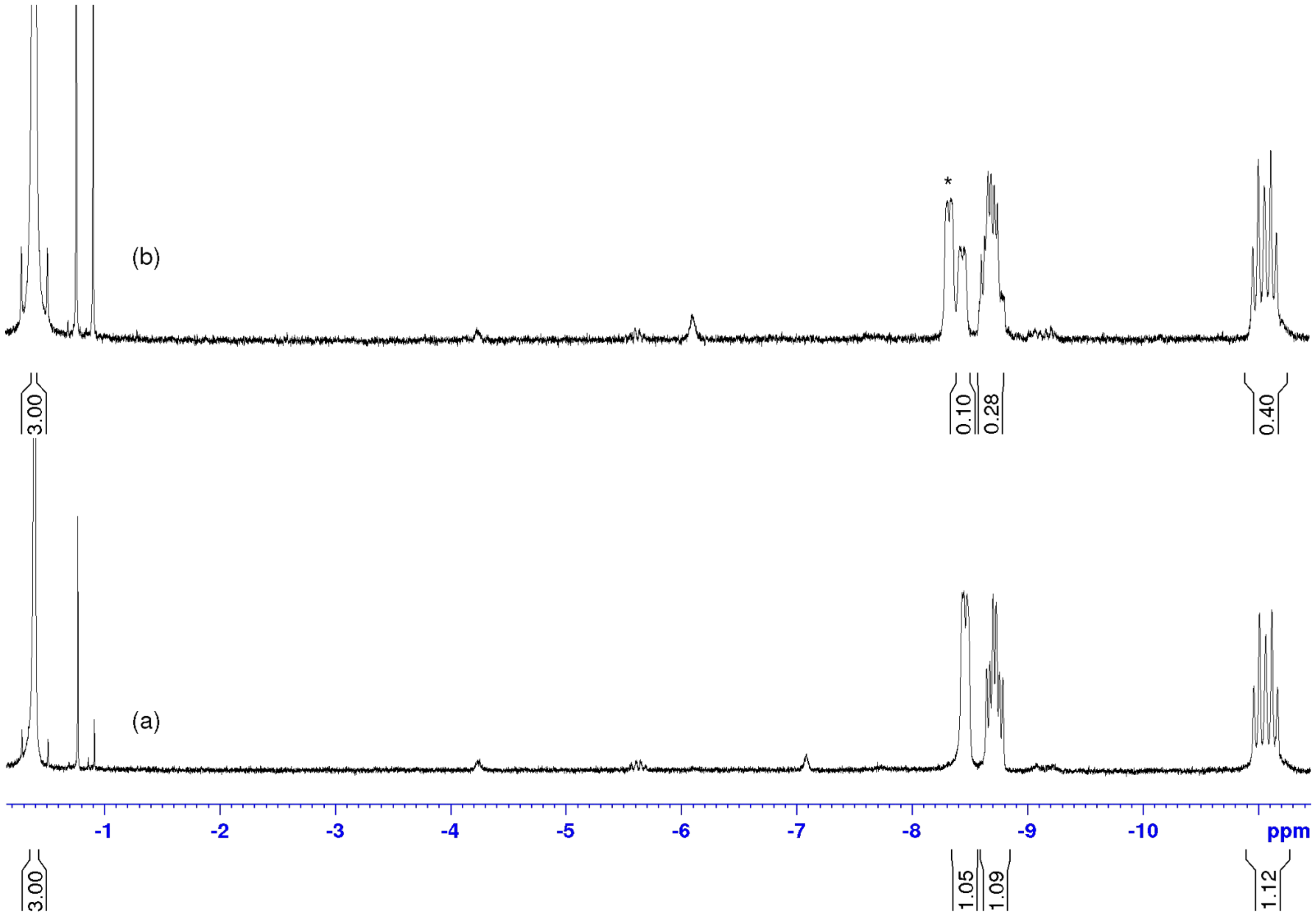

Figure S21. Low frequency region of the ^{1}H NMR spectrum (500 MHz, C_6D_6, 298 K) from the reaction of $[Ru(C_6H_4PPh_2)_2\{PPh_2C_6H_4AlMe(THF)\}H]$ **5** at 60 °C with (a) H_2 and (b) D_2. Hydride integrals relative to 3H signal of Al*Me*. * = unknown signal.

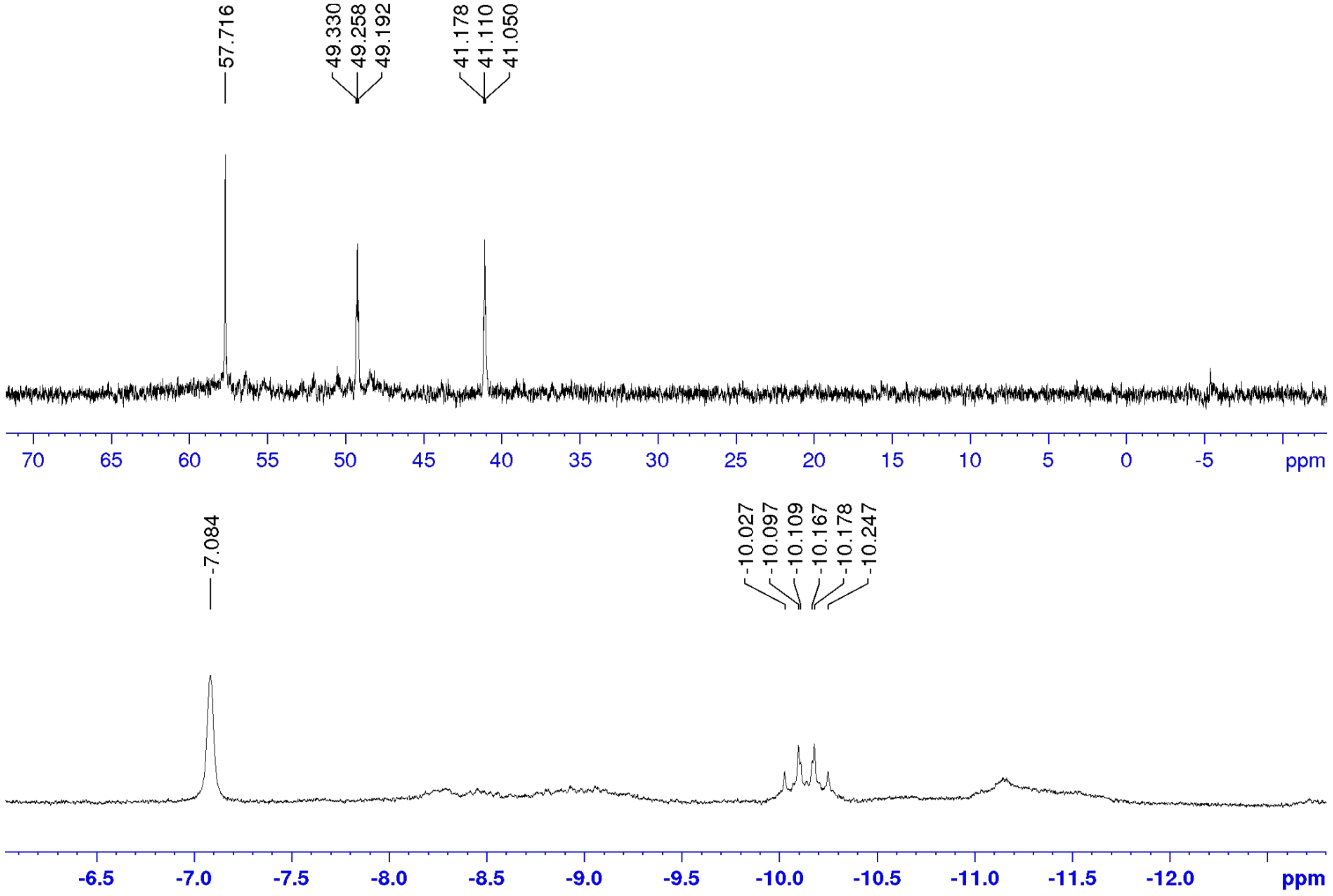

Figure S22. ^{1}H (bottom) and $^{31}P\{^{1}H\}$ (top) NMR spectra (500/202 MHz, C_6D_6, 298 K) following removal of H_2 from a sample of $[Ru(PPh_3)_2\{PPh_2C_6H_4AlMe\}H_3]$ **7** to give signals corresponding to $[Ru(PPh_3)_3(\eta^2\text{-}H_2)H_2]$ and $[Ru(PPh_3)_4H_2]$.

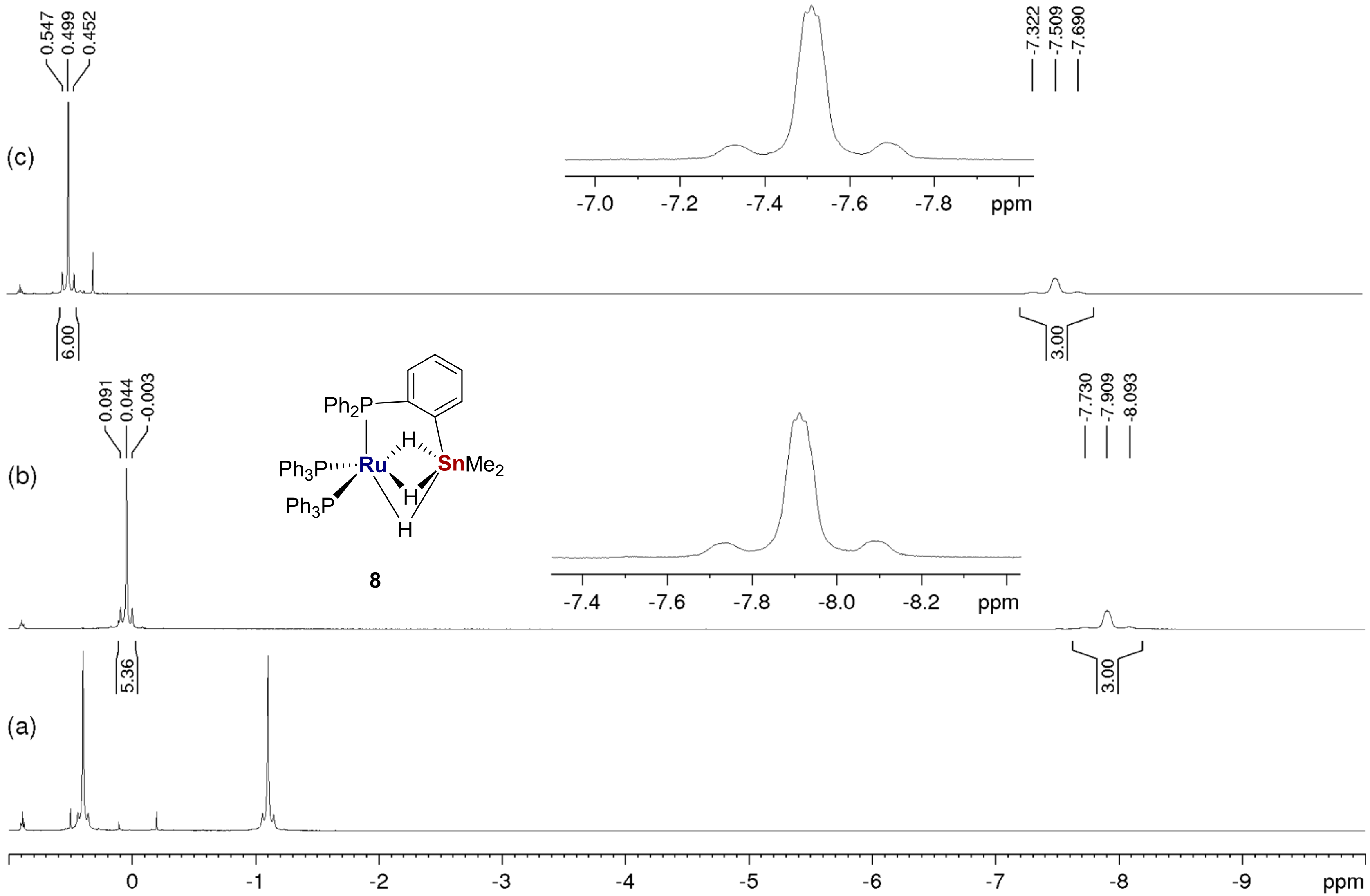

Figure S23. Low frequency region of the ^{1}H NMR spectrum (500 MHz, THF-d_8, 298 K) of $[Ru(PPh_3)(C_6H_4PPh_2)\{PPh_2C_6H_4SnMe_2\}]$ **6** (a) before and (b) 15 min after addition of 1 atm H_2 to give **8**. (c) shows the same reaction occurs in C_6D_6. Samples were unshaken to avoid precipitation of **8**.

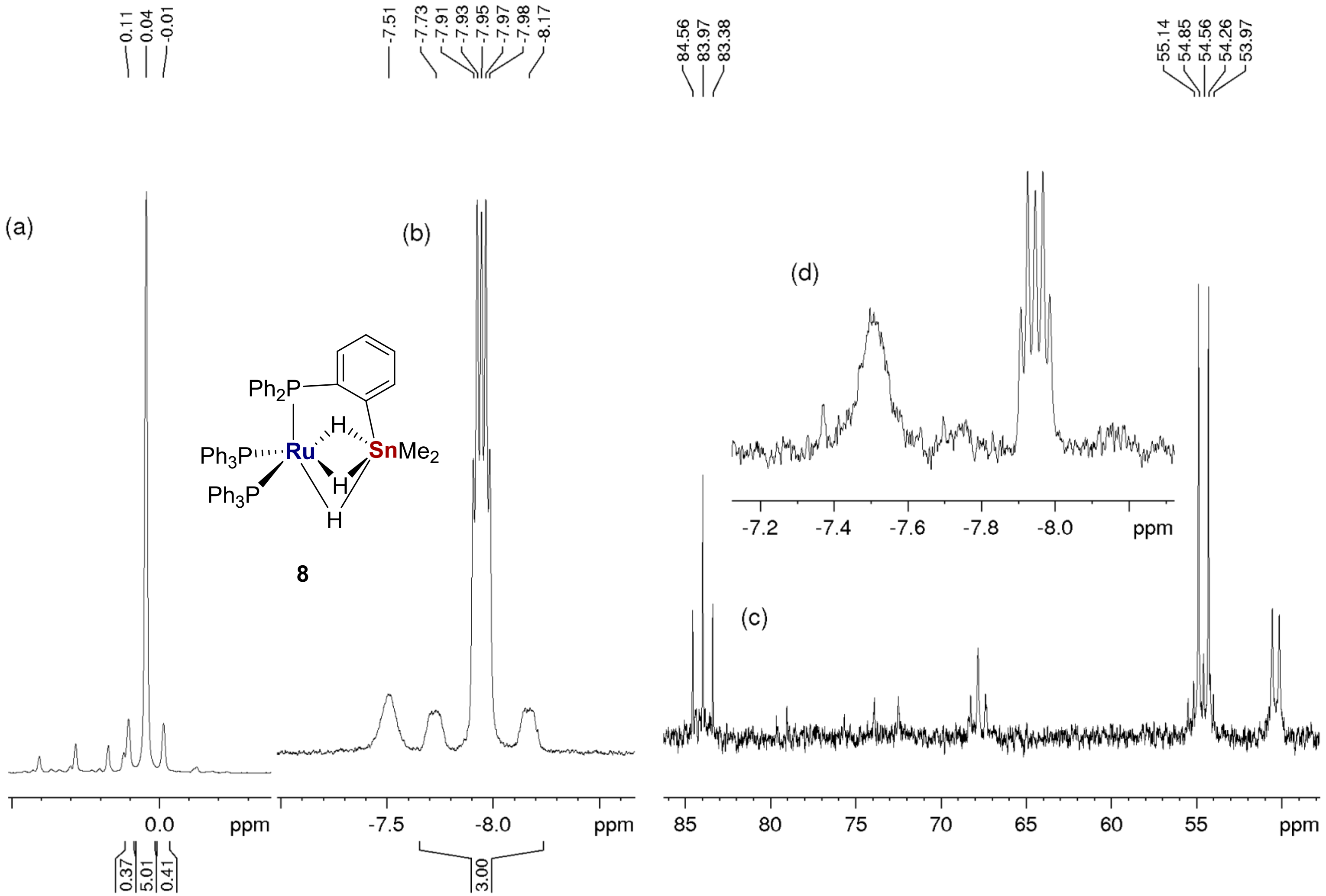

Figure S24. ^{1}H (400 MHz, 332 K) and $^{31}P\{^{1}H\}$ (162 MHz, 332 K) NMR spectra from reaction of $[Ru(PPh_3)(C_6H_4PPh_2)\{PPh_2C_6H_4SnMe_2\}]$ **6** and 1 atm H_2 in THF-d_8. (a) SnMe_2, (b) RuH_3 and (c) $^{31}P\{^{1}H\}$ signals of **8**. (d) ^{1}H NMR evidence for degradation with growth of a new hydride signal at δ -7.5 after overnight at 332 K.

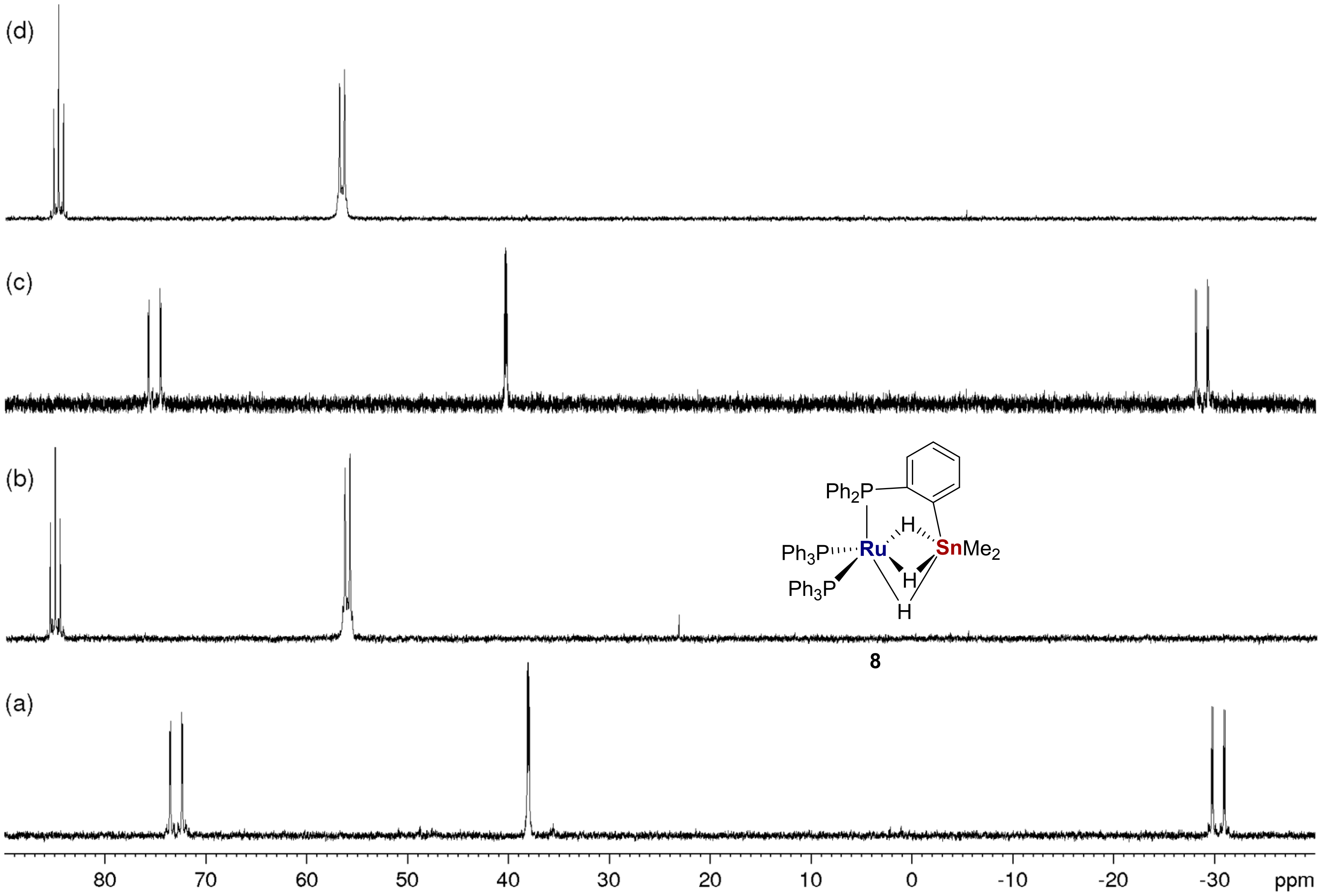

Figure S25. $^{31}P\{^1H\}$ NMR spectra (202 MHz, 298 K) of (a) [Ru(PPh_3)($C_6H_4PPh_2$){$PPh_2C_6H_4SnMe_2$}] **6** in THF-d_8 (a) before and (b) 15 min after addition of 1 atm H_2 to give **8**. (c) and (d) show the corresponding spectra in C_6D_6. Samples were not shaken to avoid precipitation.

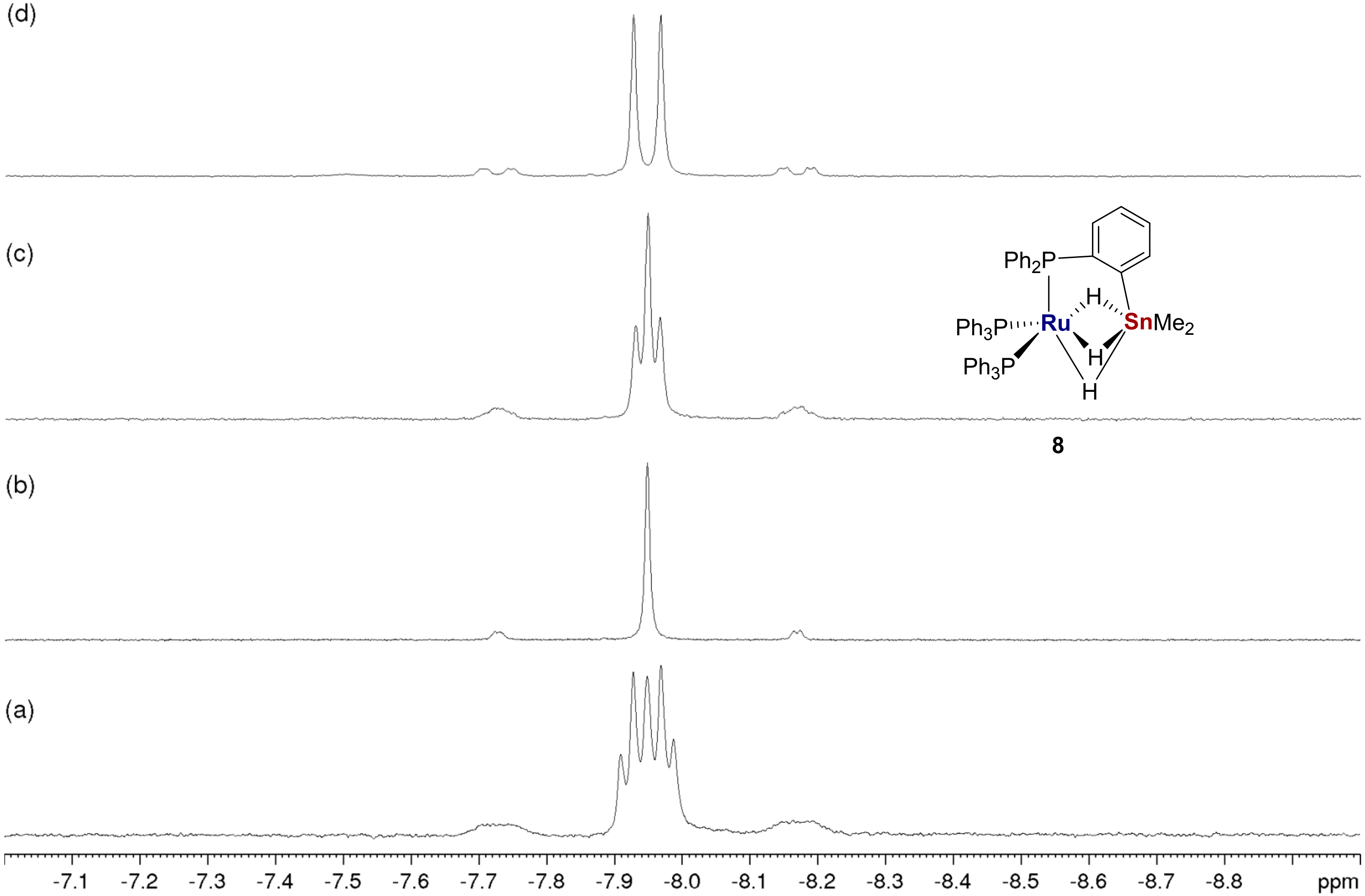

Figure S26. Hydride region (400 MHz, THF-d_8, 332 K) of the (a) ^1H, (b) $^1H\{^{31}P\}$ (broadband), (c) $^1H\{^{31}P\}$ (selective at 84 ppm) and (d) $^1H\{^{31}P\}$ (selective at 55 ppm) NMR spectra of the reaction of $[Ru(PPh_3)(C_6H_4PPh_2)\{PPh_2C_6H_4SnMe_2\}]$ **6** with H_2 to give **8**.

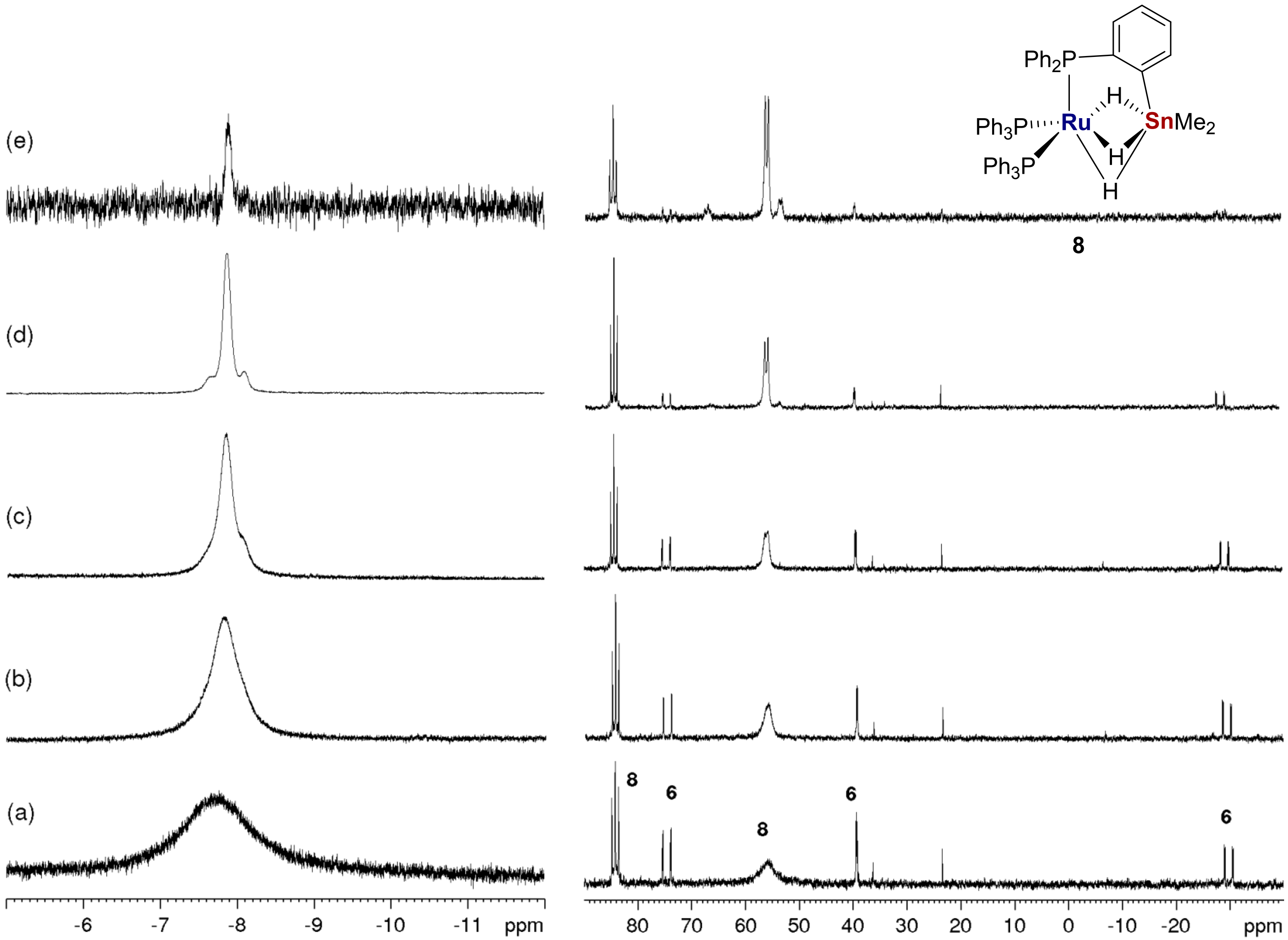

Figure 27. Hydride region of the ^{1}H (400 MHz) and $^{31}P\{^{1}H\}$ NMR (162 MHz, THF-d_8) spectra showing sharpening of signals for **8** and disappearance of $[Ru(PPh_3)(C_6H_4PPh_2)\{PPh_2C_6H_4SnMe_2\}]$ **6** under 1 atm H_2 at (a) 223 K, (b) 236 K, (c) 246 K, (d) 259 K and (e) 272 K.

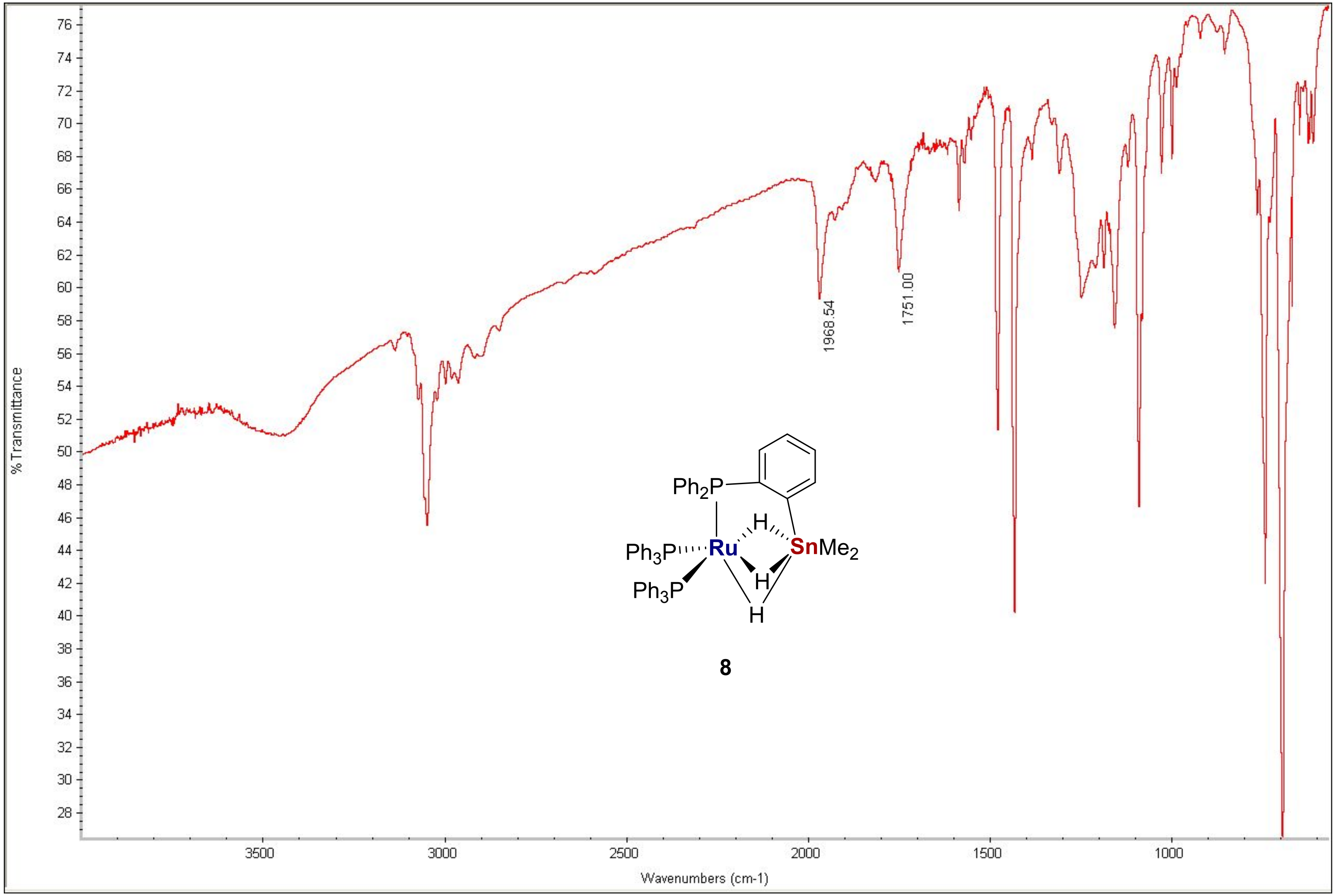

Figure S28. IR spectrum (KBr) of **8** precipitated by shaking a THF solution of [Ru(PPh_3)($C_6H_4PPh_2$){$PPh_2C_6H_4SnMe_2$}] **6** under H_2.

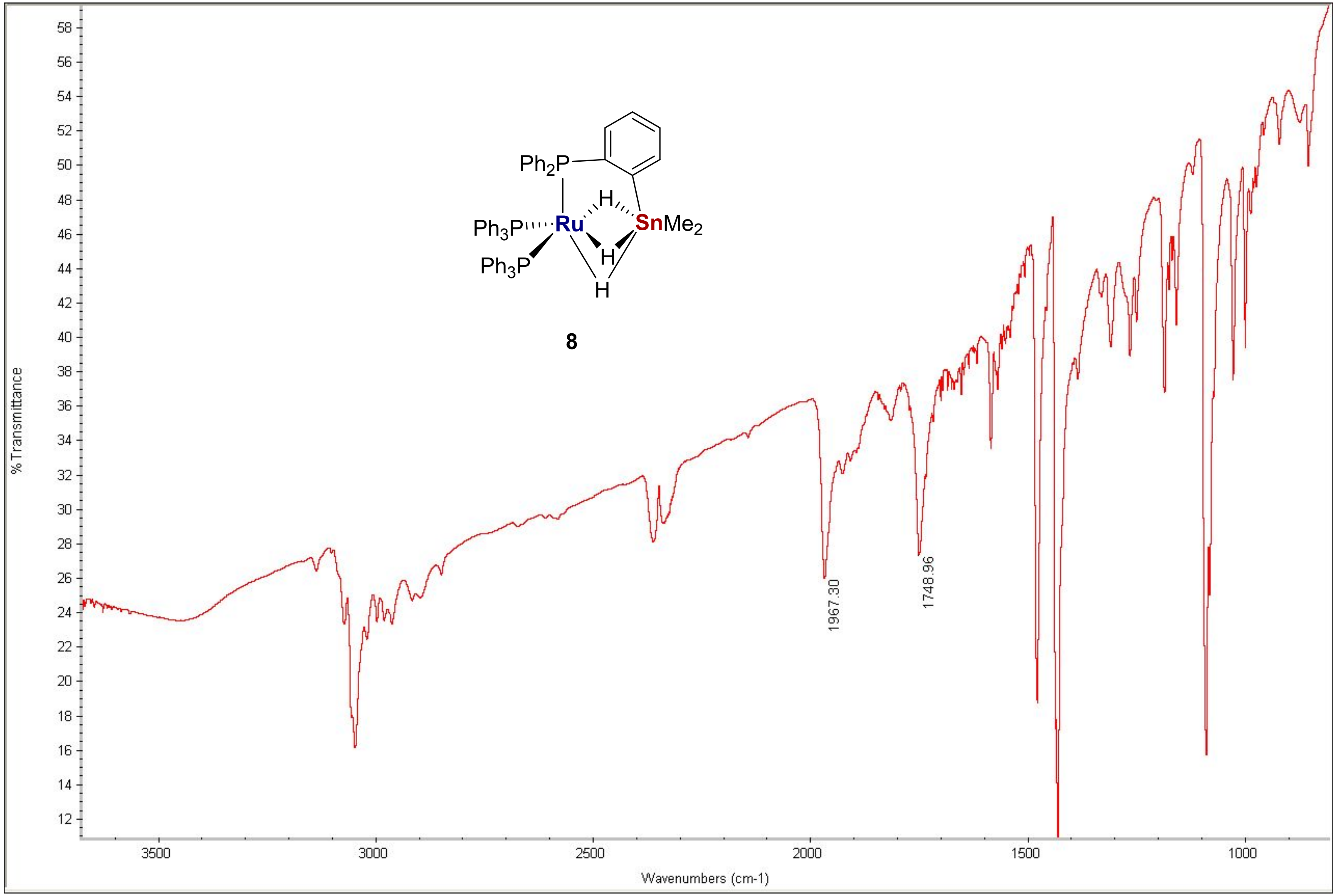

Figure S29. IR spectrum (KBr) of a solid residue of **8** formed following evaporation of a C_6D_6 solution of [Ru(PPh_3)($C_6H_4PPh_2$){$PPh_2C_6H_4SnMe_2$}] **6** through which 1 atm H_2 was allowed to slowly diffuse.

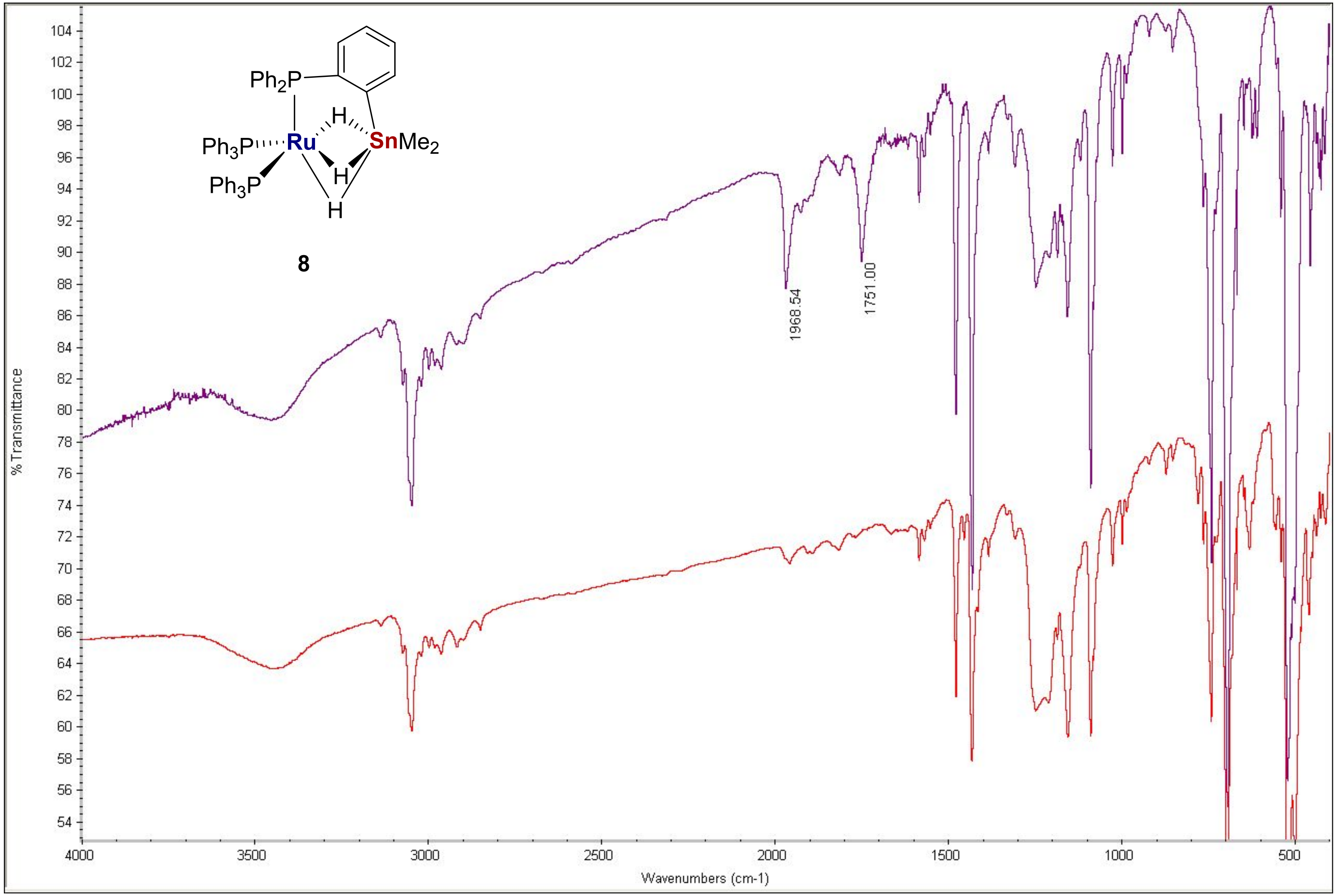

Figure S30. Overlaid IR spectra (KBr) of solid samples of [Ru(PPh_3)($C_6H_4PPh_2$){$PPh_2C_6H_4SnMe_2$}] **6** stirred under (top) 1 atm H_2 and (bottom) 1 atm D_2 showing the absence of the bands assigned to $\nu_{Ru\text{-}H}$ in the latter case.

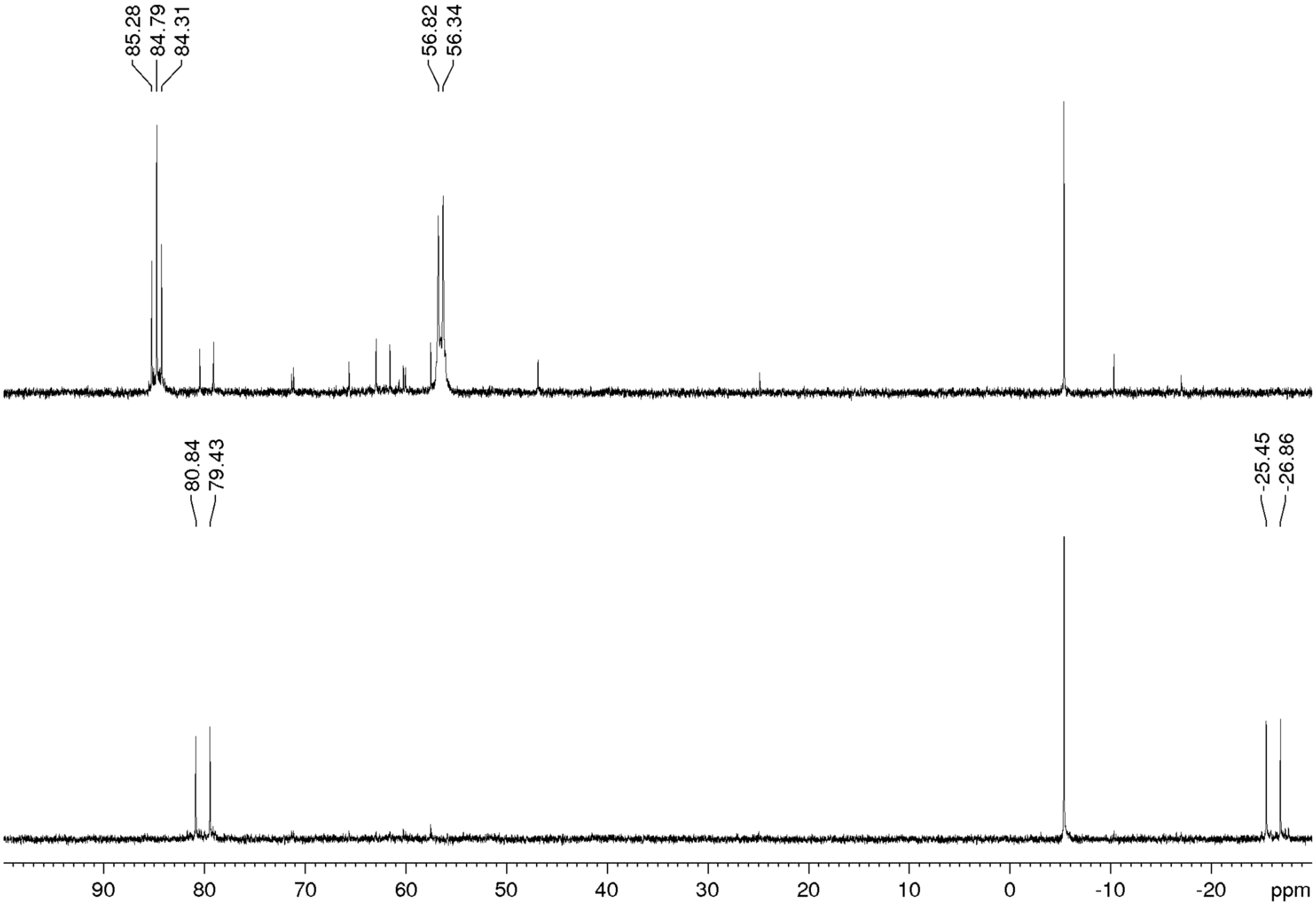

Figure S31. $^{31}P\{^{1}H\}$ NMR spectra (202 MHz, 298 K) showing (bottom) formation of [Ru(NC_5H_5)($C_6H_4PPh_2$)($PPh_2C_6H_4SnMe_2$)] from reaction of [Ru(PPh_3)($C_6H_4PPh_2$){$PPh_2C_6H_4SnMe_2$}] **6** with excess pyridine in C_6D_6 and (top) formation of **8** upon adding H_2 to the sample.

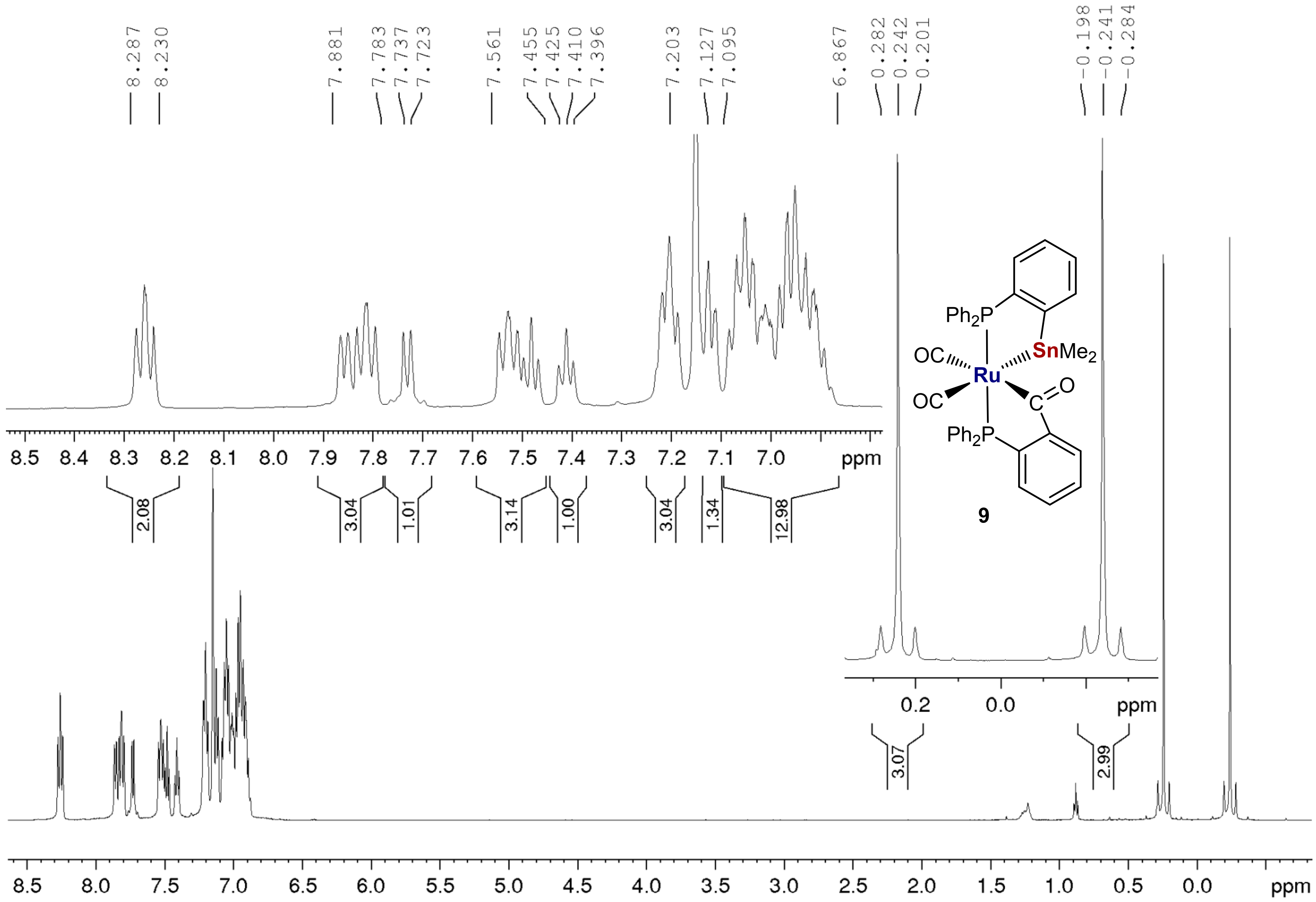

Figure S32. ^{1}H NMR spectrum (500 MHz, C_6D_6, 298 K) of [Ru(CO)$_2$(C(O)C$_6$H$_4$PPh$_2$)(PPh$_2$C$_6$H$_4$SnMe$_2$)] **9**, with insets of aromatic and SnMe_2 resonances.

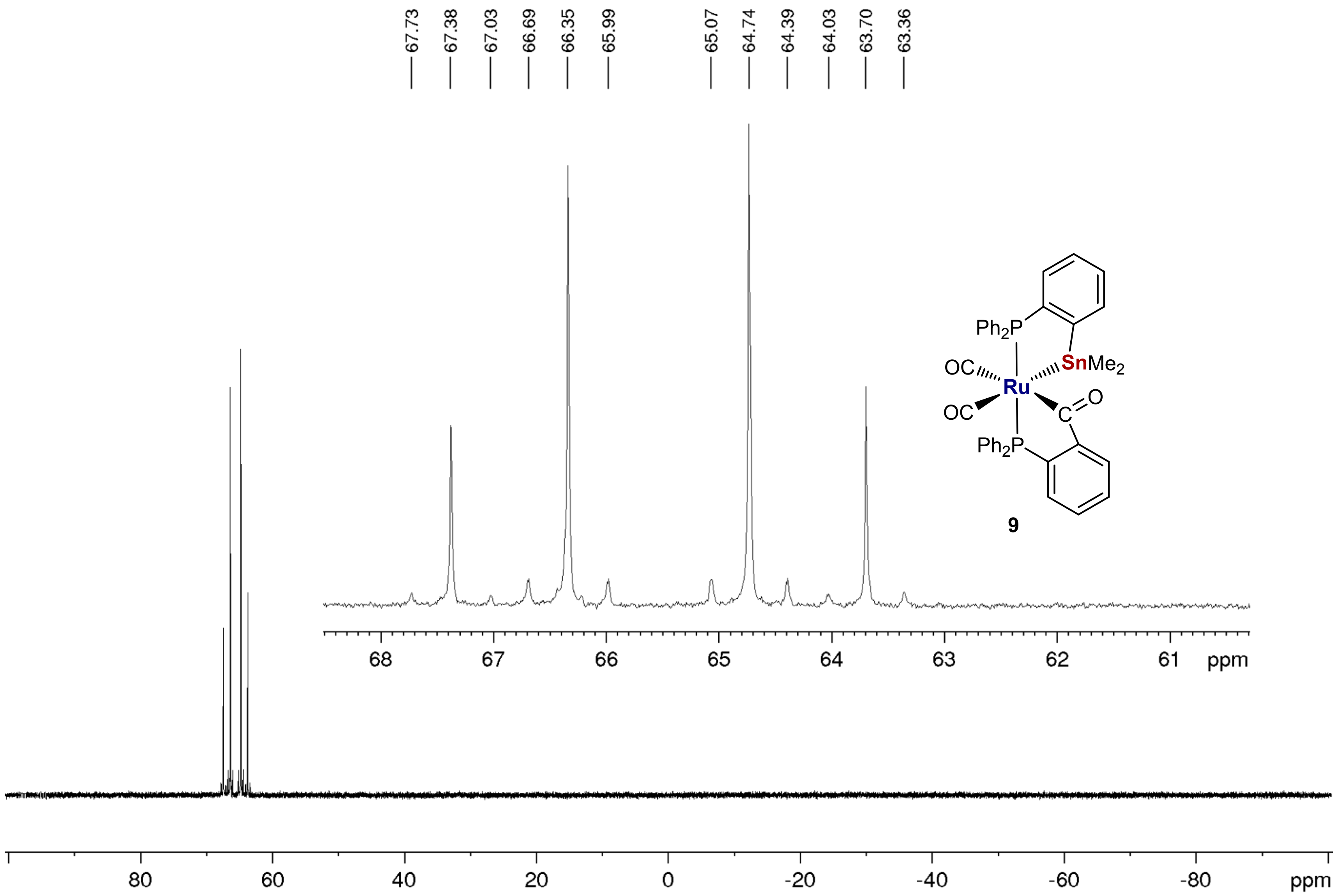

Figure S33. $^{31}P\{^{1}H\}$ NMR spectrum (202 MHz, C_6D_6, 298 K) of $[Ru(CO)_2(C(O)C_6H_4PPh_2)(PPh_2C_6H_4SnMe_2)]$ **9**.

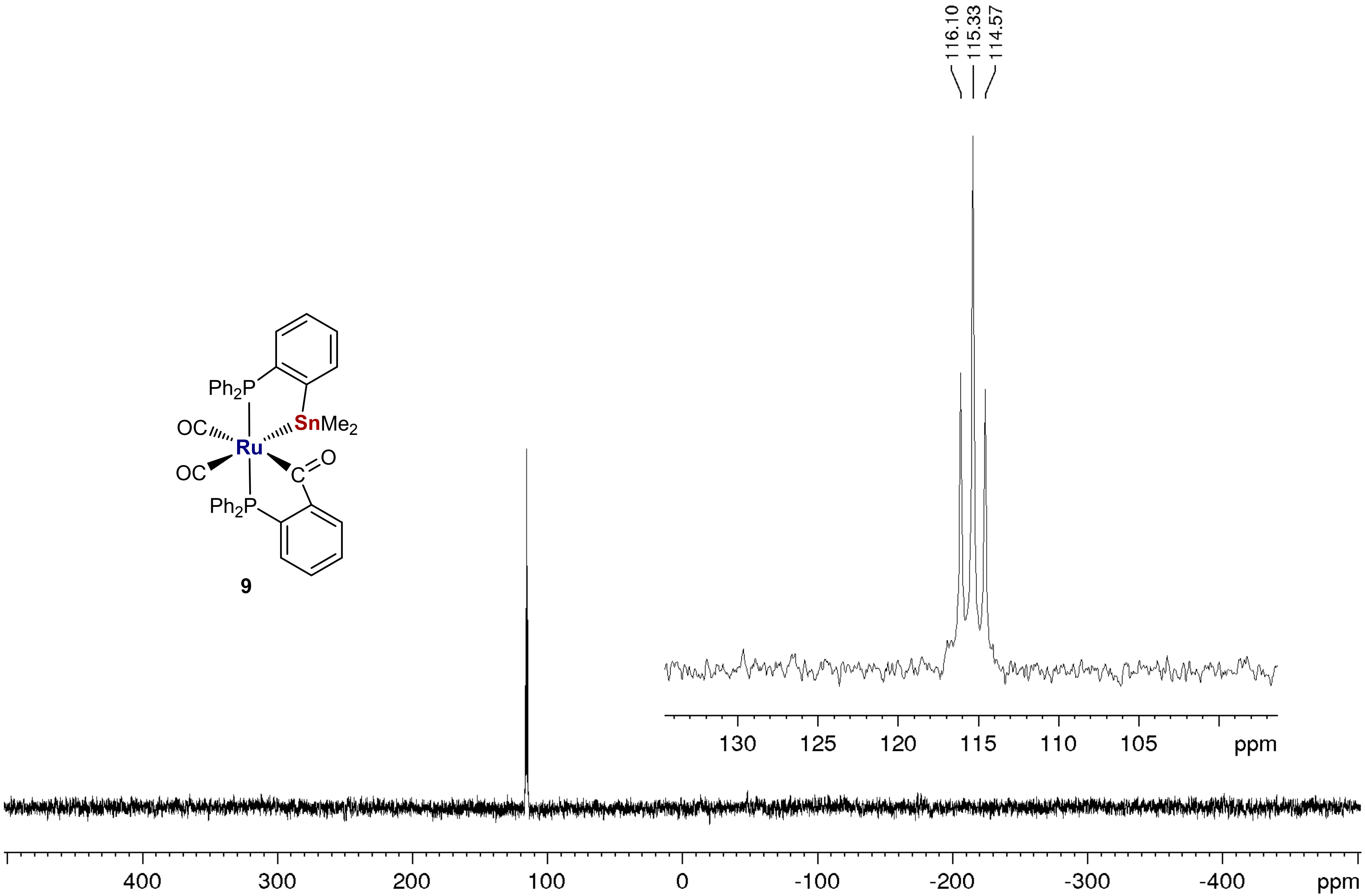

Figure S34. ${}^{119}Sn\{{}^{1}H\}$ NMR spectrum (187 MHz, C_6D_6, 298 K) of $[Ru(CO)_2(C(O)C_6H_4PPh_2)(PPh_2C_6H_4SnMe_2)]$ **9**.

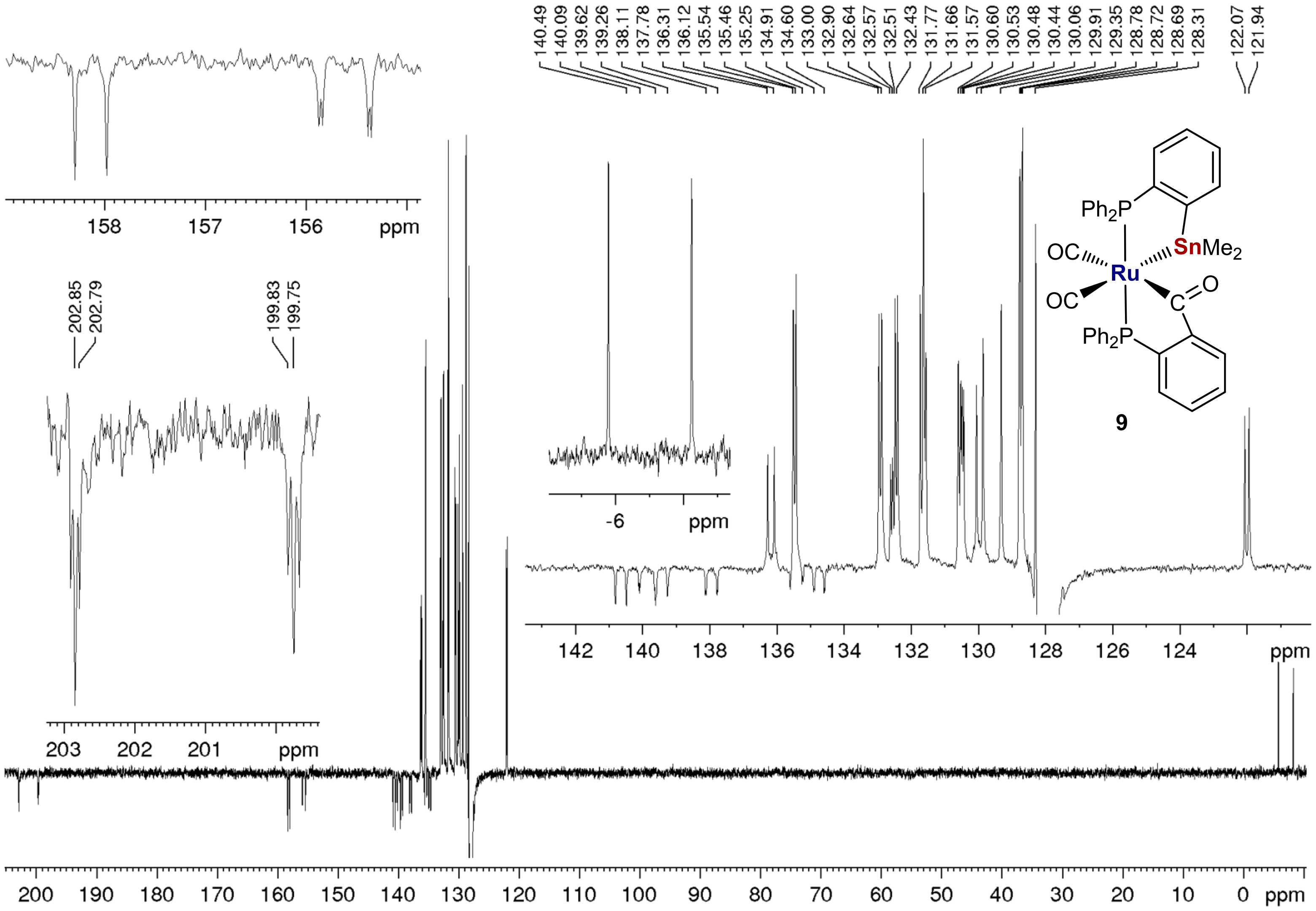

Figure S35. $^{13}C\{^1H\}$ NMR spectrum (126 MHz, C_6D_6, 298 K) of $[Ru(CO)_2(C(O)C_6H_4PPh_2)(PPh_2C_6H_4SnMe_2)]$ **9**, with insets of high and low frequency regions, plus aromatic signals.

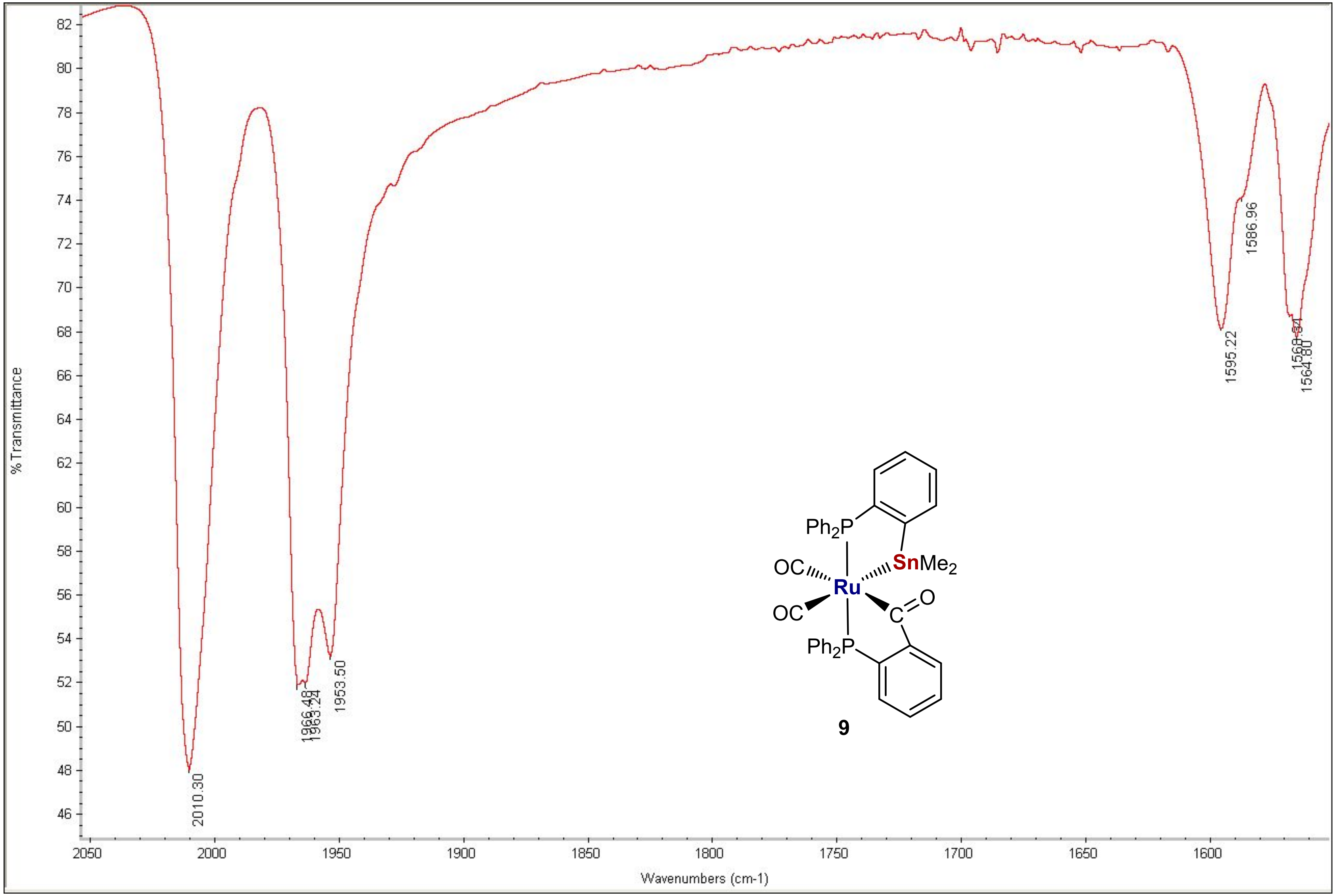

Figure S36. Carbonyl region of the IR spectrum (KBr) of $[Ru(CO)_2(C(O)C_6H_4PPh_2)(PPh_2C_6H_4SnMe_2)]$ **9**.

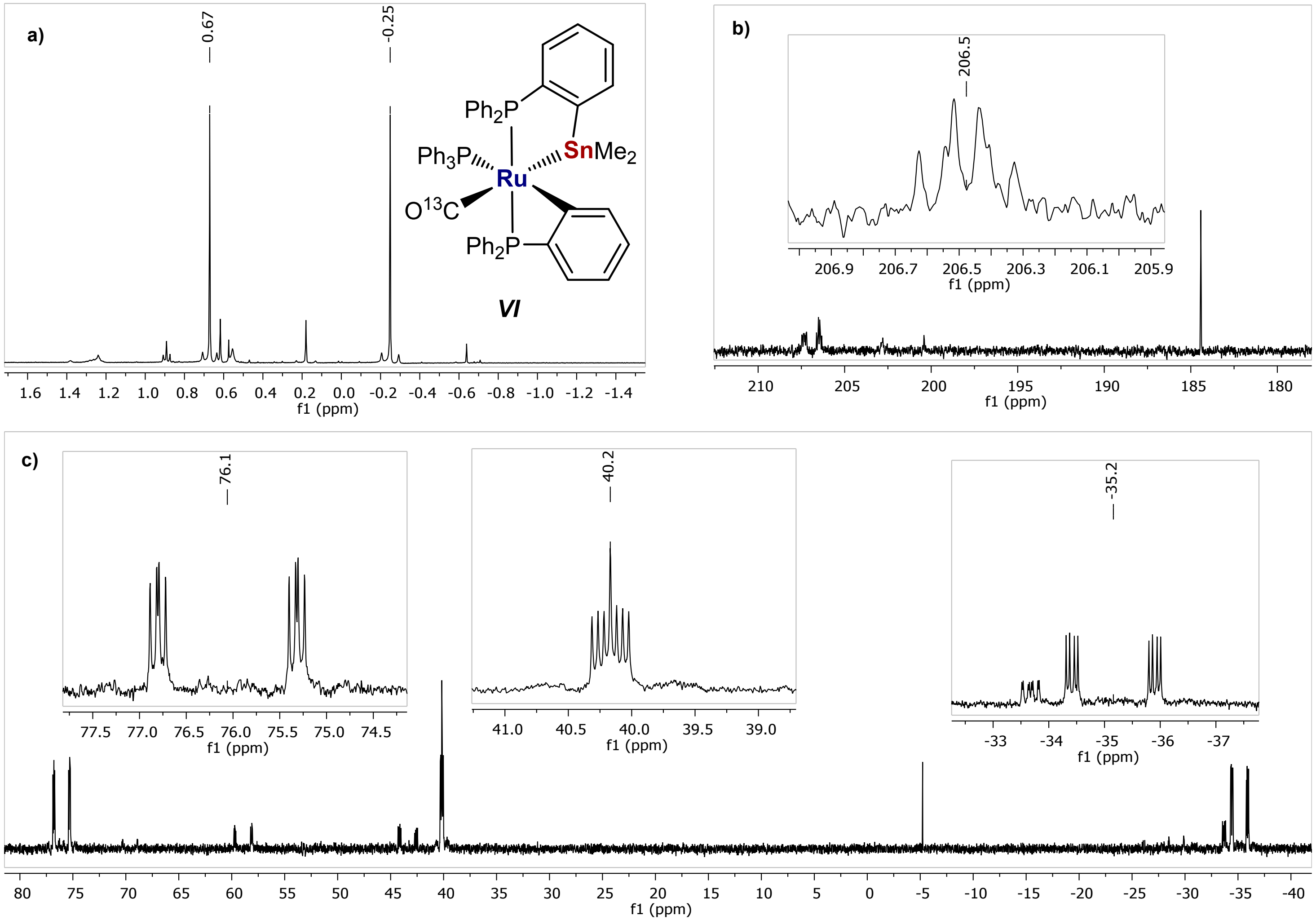

Figure S37. NMR spectra of ***VI-^{13}CO*** from reaction of **6** with ^{13}CO (C_6D_6, 298 K). (a) Sn*Me* region of the ^{1}H NMR spectrum (400 MHz) 15 min after addition of ^{13}CO, (b) Carbonyl region of the ^{13}C{^{1}H} NMR spectrum (101 MHz) 0.5-1 h after addition and (c) ^{31}P{^{1}H} NMR spectrum (162 MHz) 20 min after addition.

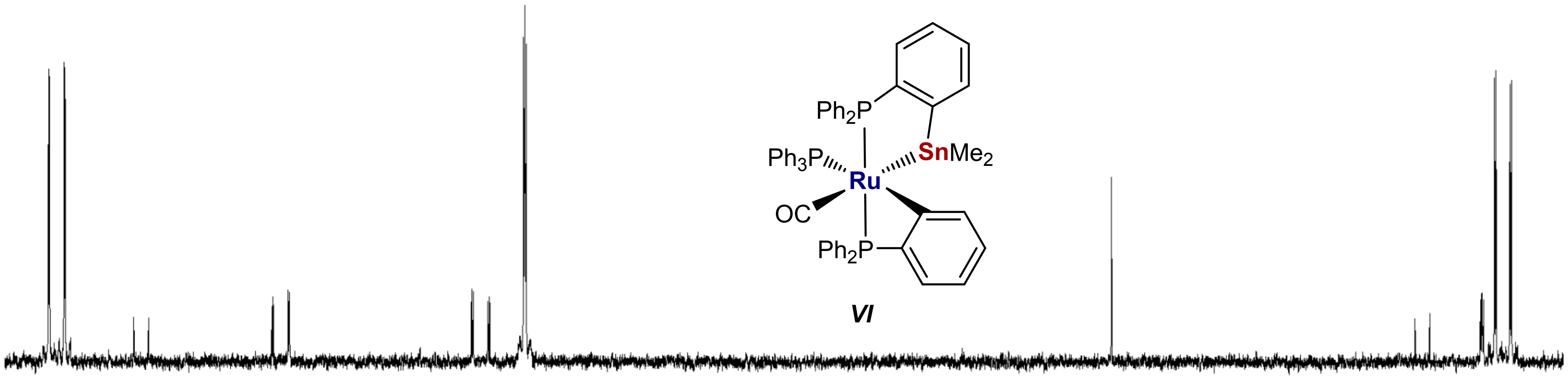
Ph2P
Ph3P
SnMe2
Ru
OC
Ph2P
VI

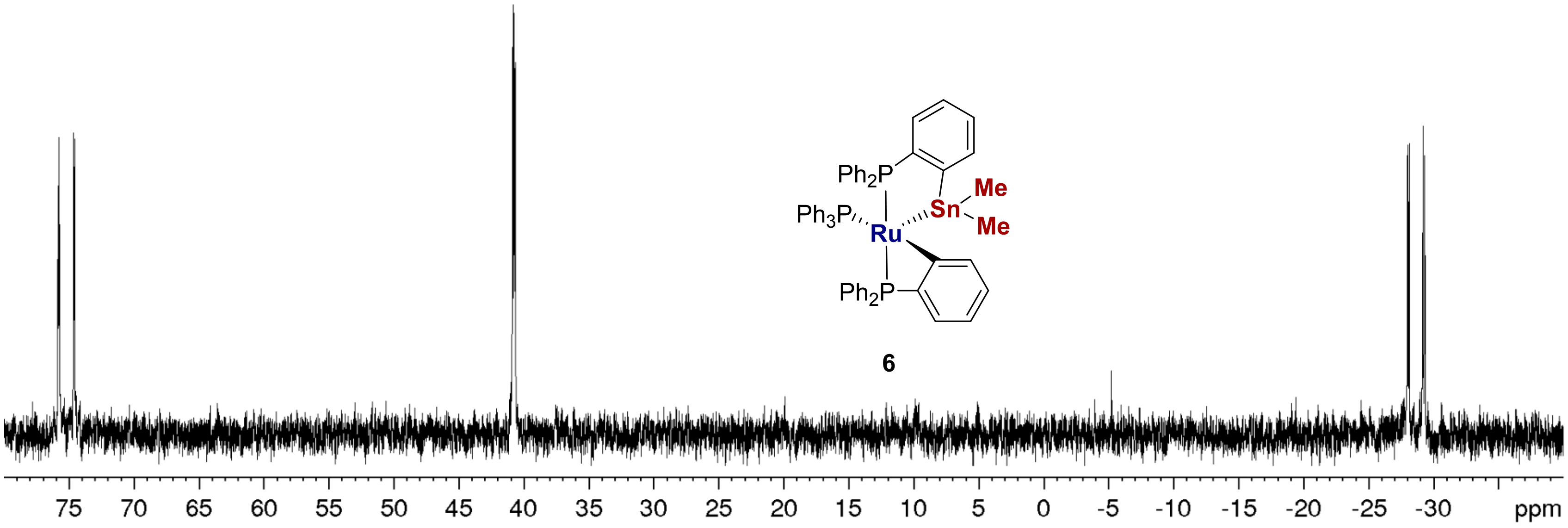
Ph2P
Ph3P
Sn
Me
Me
Ru
Ph2P
6
75 70 65 60 55 50 45 40 35 30 25 20 15 10 5 0 -5 -10 -15 -20 -25 -30 ppm

Figure S38. $^{31}P\{^{1}H\}$ NMR spectra (202 MHz, $C_6D_5CD_3$, 298 K) showing (top) formation of ***VI*** as the main species 5 min after addition of 1 atm ^{12}CO to (bottom) **6**.

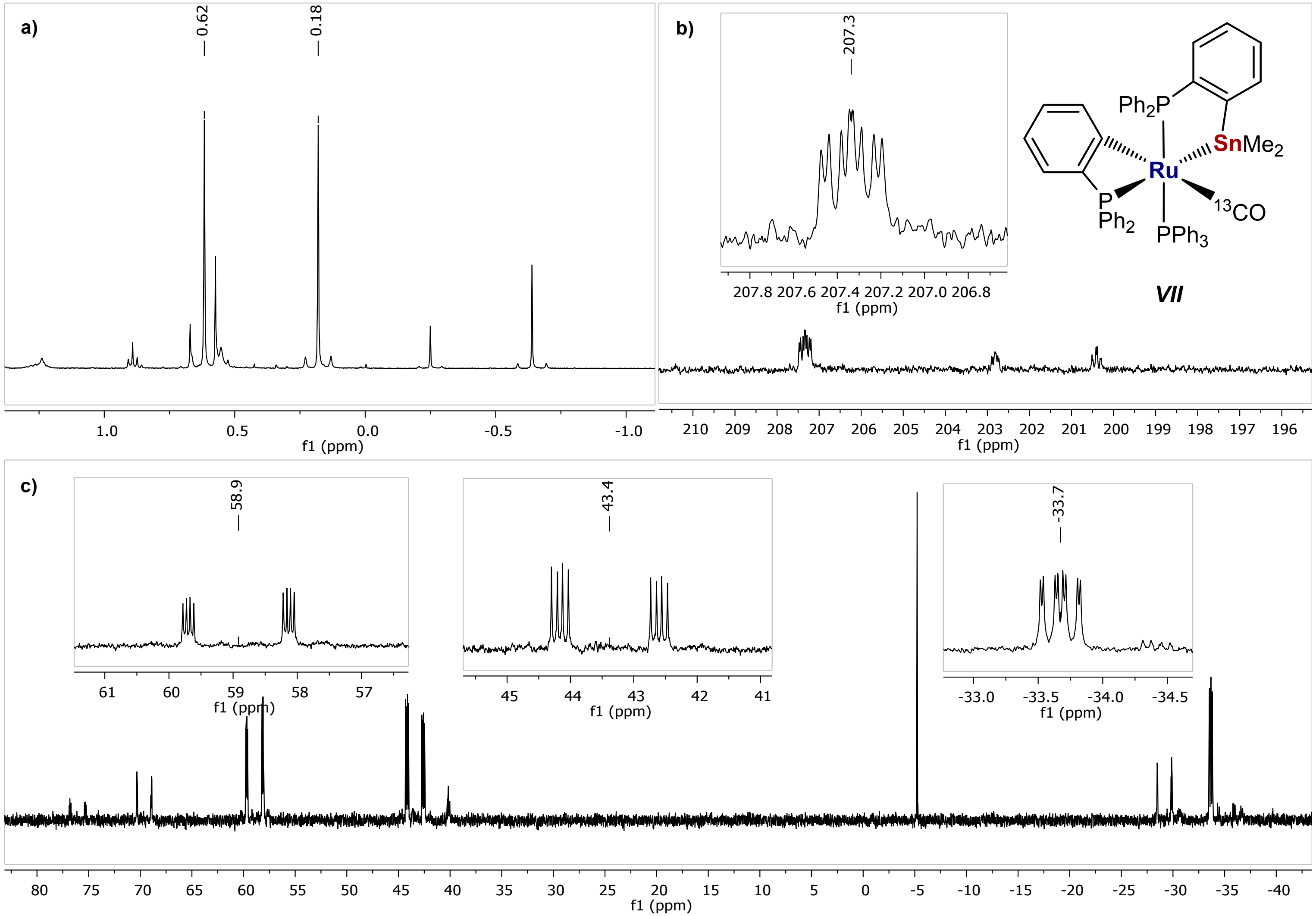

Figure S39. Selected NMR spectra of ***VII-^{13}CO*** formed in the reaction of **6** with ^{13}CO. (a) Sn-CH_3 region of the ^{1}H NMR spectrum (400 MHz, C_6D_6, 298 K) recorded 2.5 h after addition of ^{13}CO, {b) ^{13}C{^{1}H} NMR spectrum (101 MHz, C_6D_6, 298 K) recorded 2.5-3 h after addition of ^{13}CO and (c) ^{31}P{^{1}H} NMR spectrum (162 MHz, C_6D_6, 298 K) recorded 2.5 h after addition of ^{13}CO.

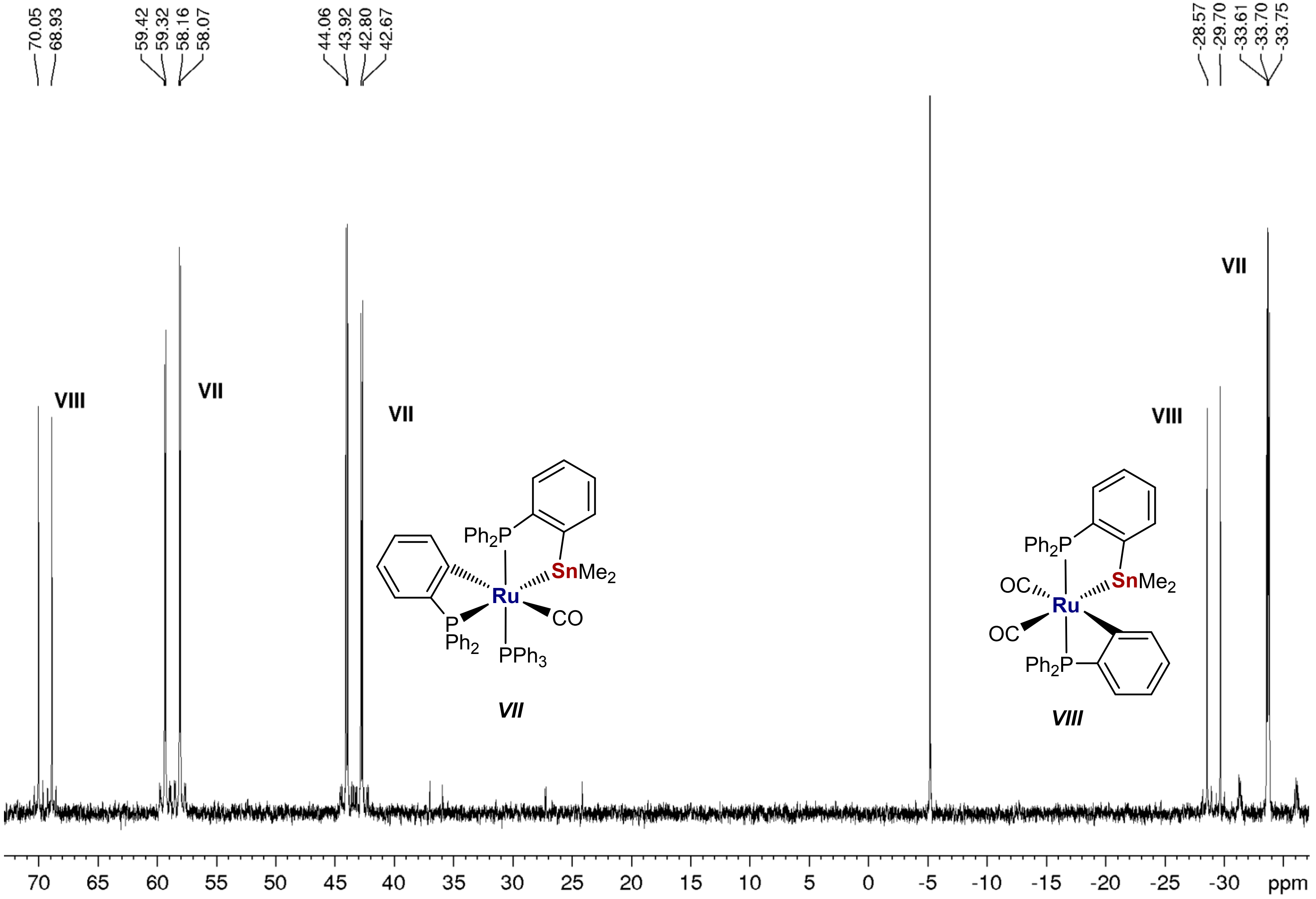

Figure S40. $^{31}P\{^{1}H\}$ NMR spectra (202 MHz, $C_6D_5CD_3$, 298 K) recorded 19.5 h after exposure of **6** to 1 atm ^{12}CO showing the formation of ***VII*** and ***VIII***.

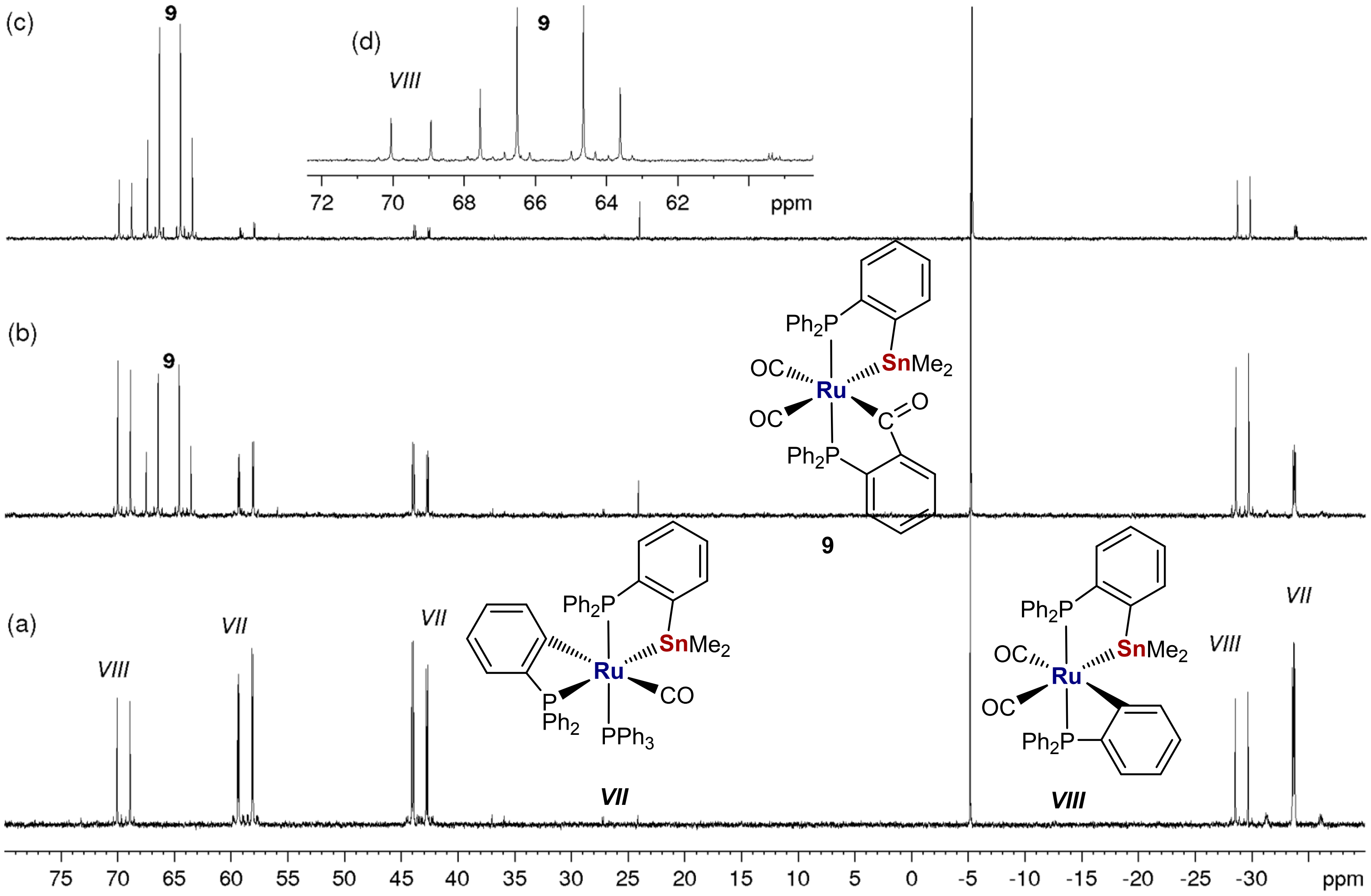

Figure S41. $^{31}P\{^{1}H\}$ NMR spectra (202 MHz, $C_6D_5CD_3$, 298 K) showing evolution of species (see **Scheme 9** for structures) in the reaction of **6** with ^{12}CO. (Bottom) After 19.5 h at room temperature, (middle) an additional 1 h at 80 ºC and (top) a total of 3 h at 80 ºC, with inset of high frequency region of spectrum.

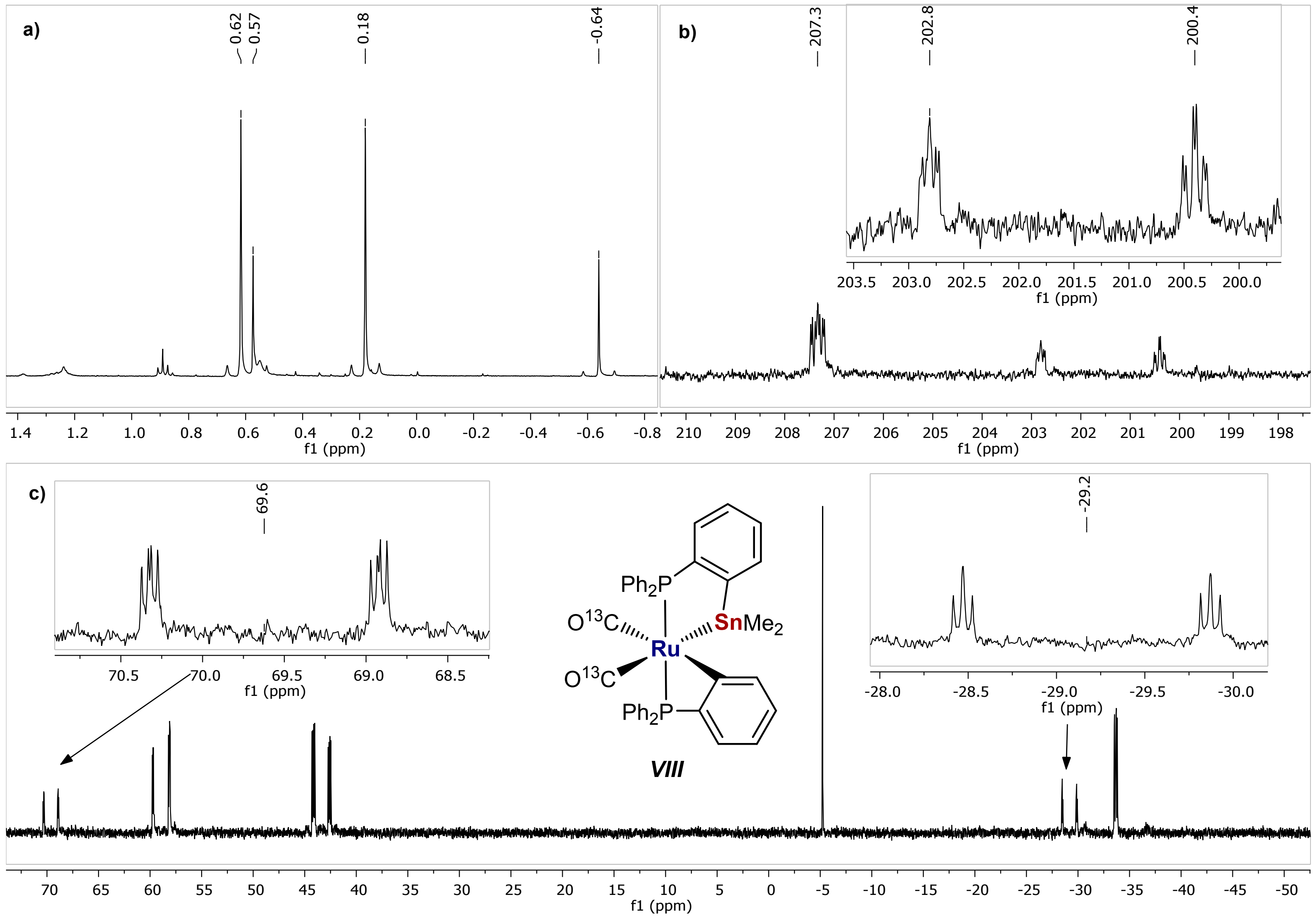

Figure S42. Selected NMR spectra of ***VIII-^{13}CO*** (see **Scheme 9** for structures) formed in the reaction of **6** with ^{13}CO. (a) Sn-CH_3 region of the ^{1}H NMR spectrum (400 MHz, C_6D_6, 298 K) recorded 20 h after addition of ^{13}CO, (b) ^{13}C{^{1}H} NMR spectrum (101 MHz, C_6D_6, 298 K) recorded 20 h after addition of ^{13}CO and (c) ^{31}P{^{1}H} NMR spectrum (162 MHz, C_6D_6, 298 K) recorded 20 h after addition of ^{13}CO.

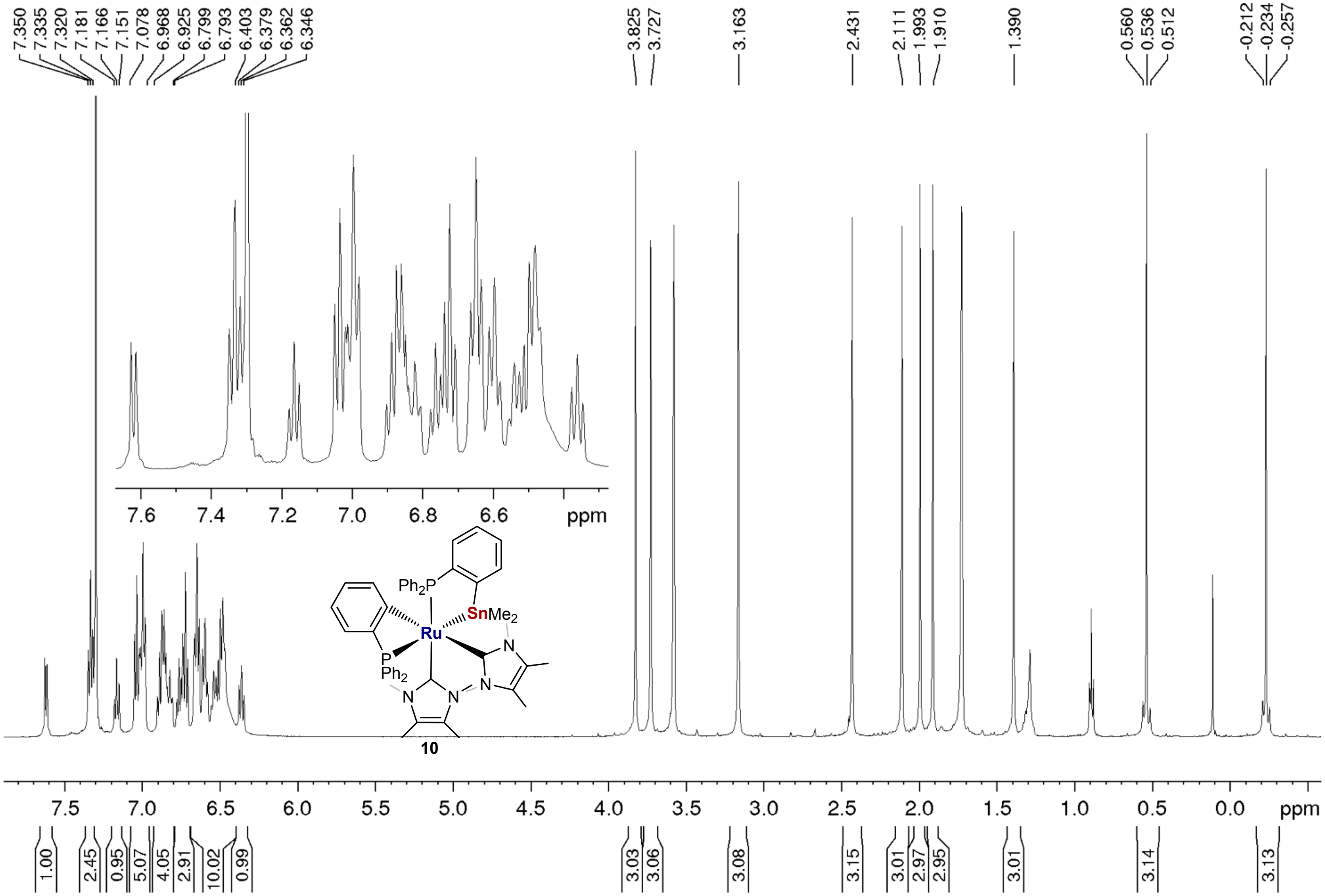

Figure S43. ^1H NMR spectrum (500 MHz, THF-d_8, 298 K) of [Ru(IMe$_4$)$_2$(C$_6$H$_4$PPh$_2$)(PPh$_2$C$_6$H$_4$SnMe$_2$)] **10**, with inset of aromatic region.

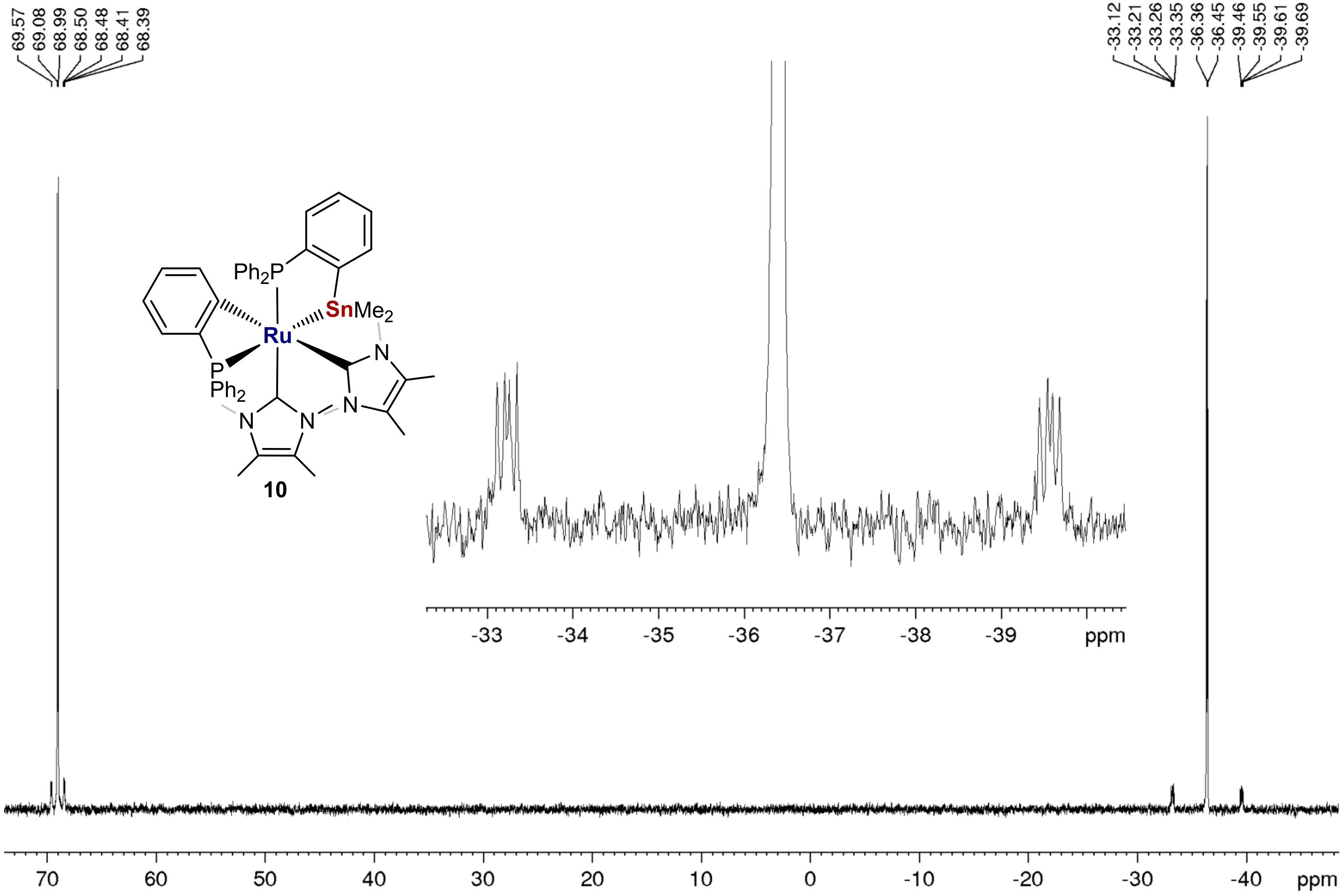

Figure S44. $^{31}P\{^{1}H\}$ NMR spectrum (202 MHz, THF-d_8, 298 K) of [Ru(IMe$_4$)$_2$(C$_6$H$_4$PPh$_2$)(PPh$_2$C$_6$H$_4$SnMe$_2$)] **10**, with inset of lowest frequency signal to highlight couplings to both ^{119}Sn and ^{117}Sn.

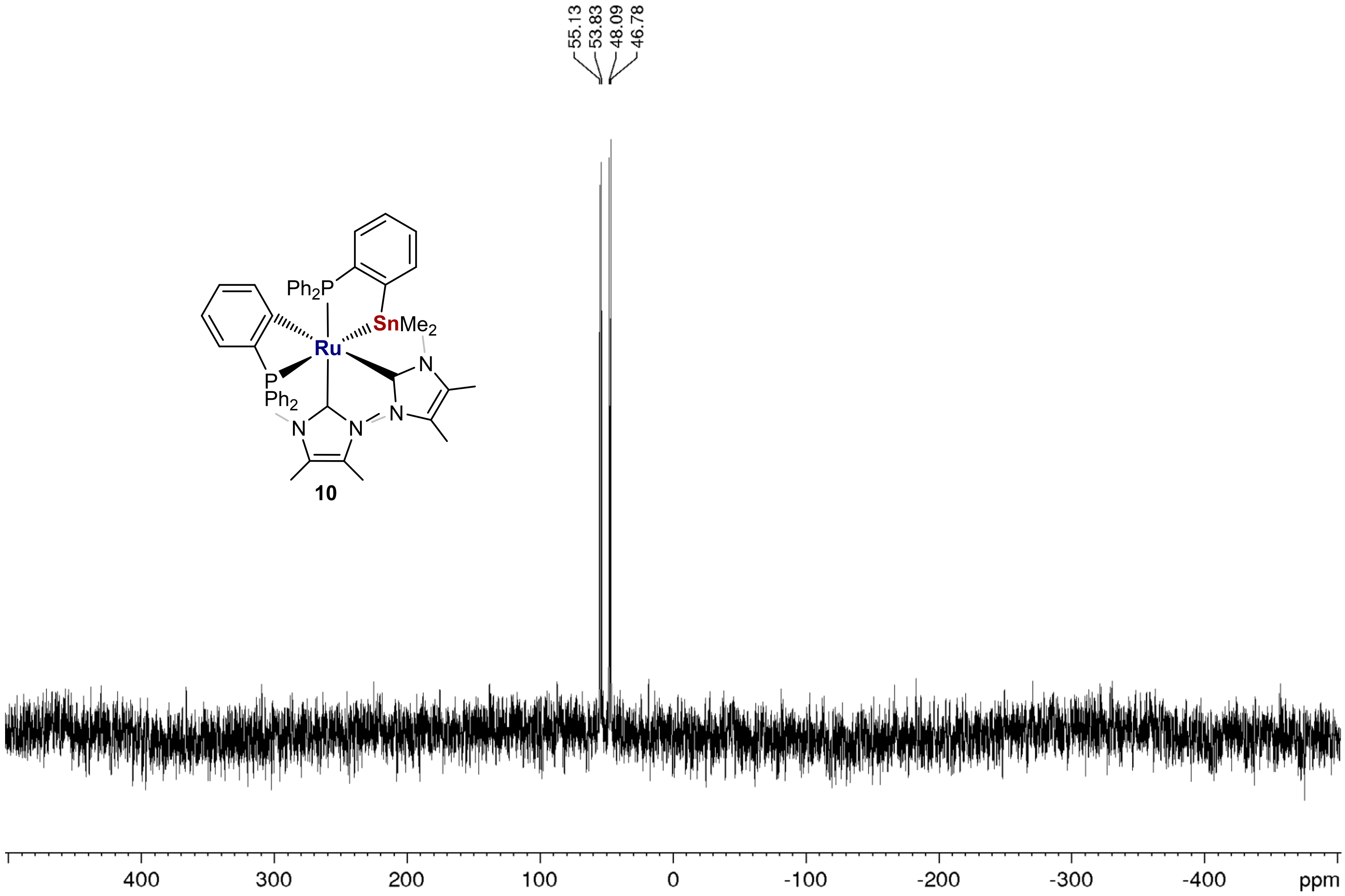

Figure S45. ^{119}Sn{^{1}H} NMR spectrum (187 MHz, THF-d_8, 298 K) of [Ru(IMe$_4$)$_2$(C$_6$H$_4$PPh$_2$)(PPh$_2$C$_6$H$_4$SnMe$_2$)] **10**.

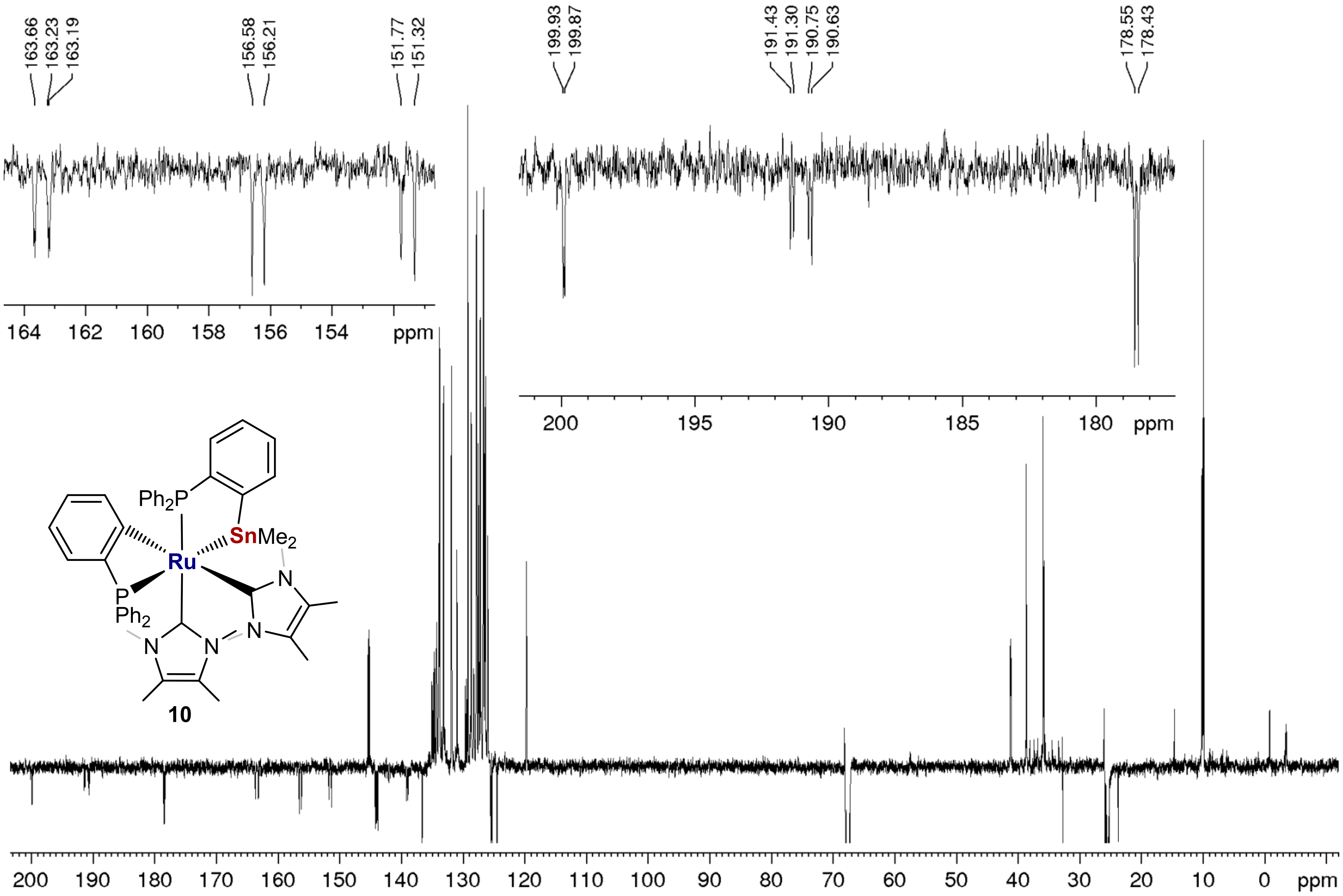

Figure S46. $^{13}C\{^{1}H\}$ DEPT-Q NMR spectrum (126 MHz, THF-d_8, 298 K) of $[Ru(IMe_4)_2(C_6H_4PPh_2)(PPh_2C_6H_4SnMe_2)]$ **10**, with insets of high frequency metalated phenyl and NHC carbenic signals.

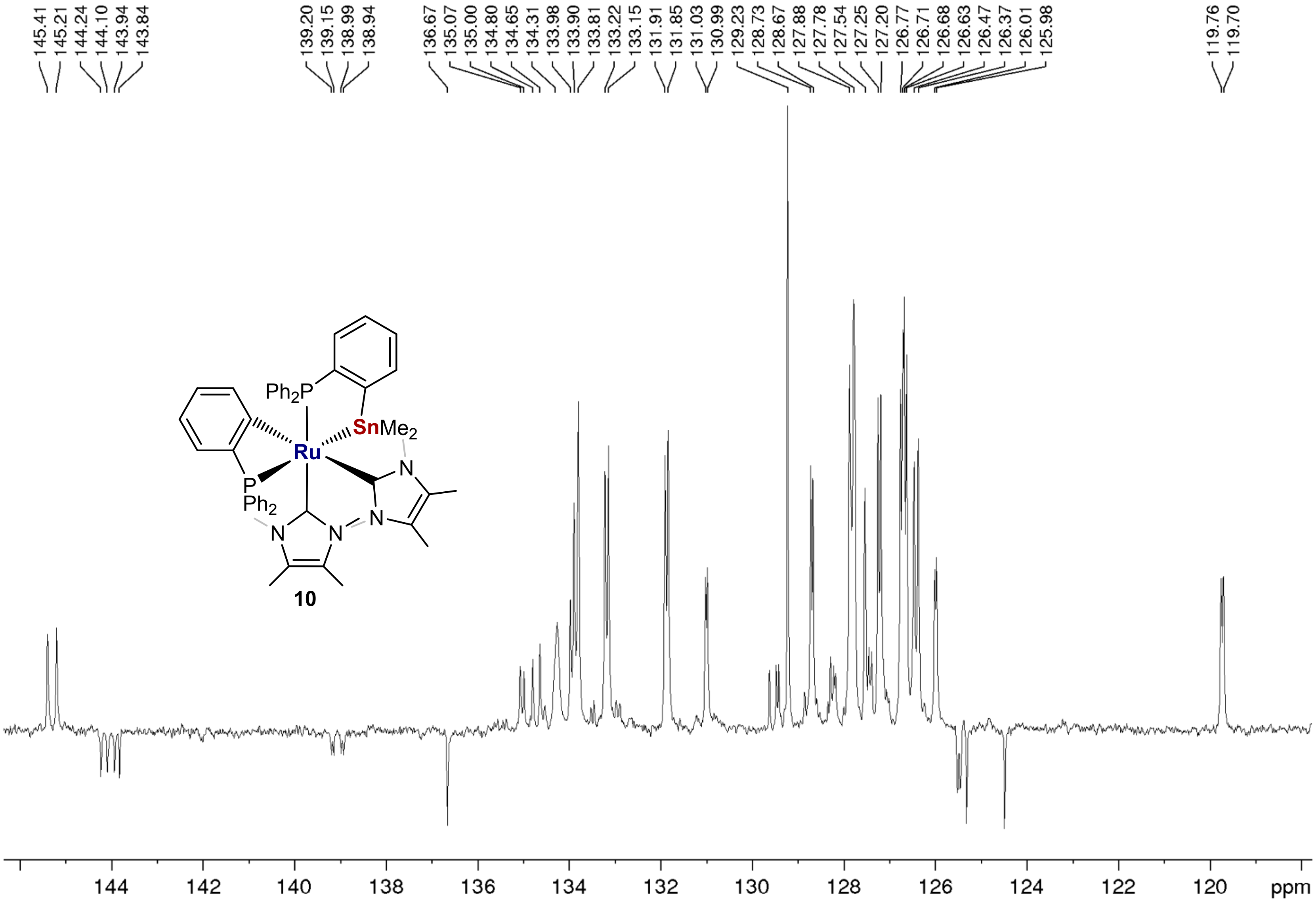

Figure S47. Aromatic region of the $^{13}C\{^1H\}$ DEPT-Q NMR spectrum (126 MHz, THF-d_8, 298 K) of [Ru(IMe$_4$)$_2$(C$_6$H$_4$PPh$_2$)(PPh$_2$C$_6$H$_4$SnMe$_2$)] **10**.

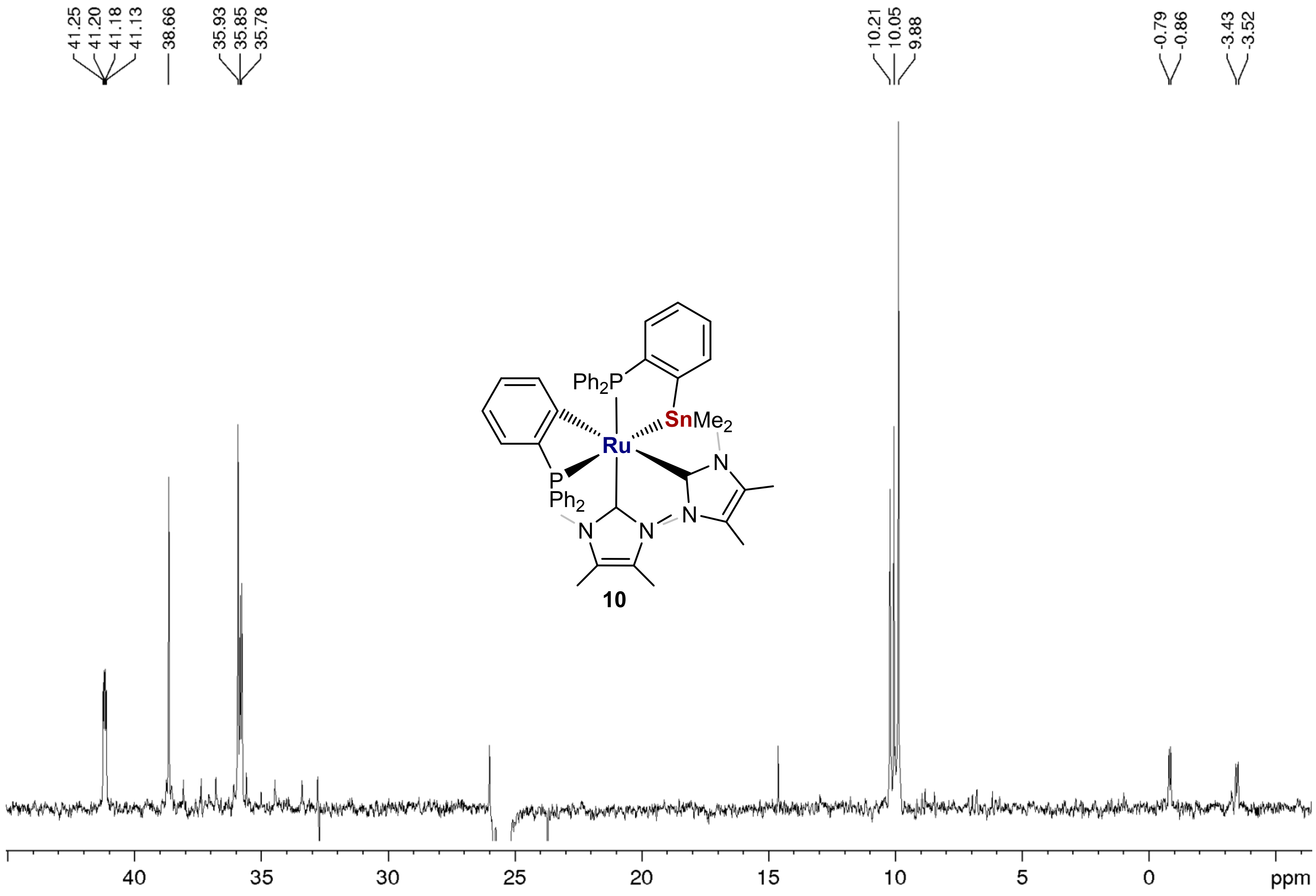

Figure S48. Low frequency region of $^{13}C\{^{1}H\}$ DEPT-Q NMR spectrum (126 MHz, THF-d_8, 298 K) of $[Ru(IMe_4)_2(C_6H_4PPh_2)(PPh_2C_6H_4SnMe_2)]$ **10**.

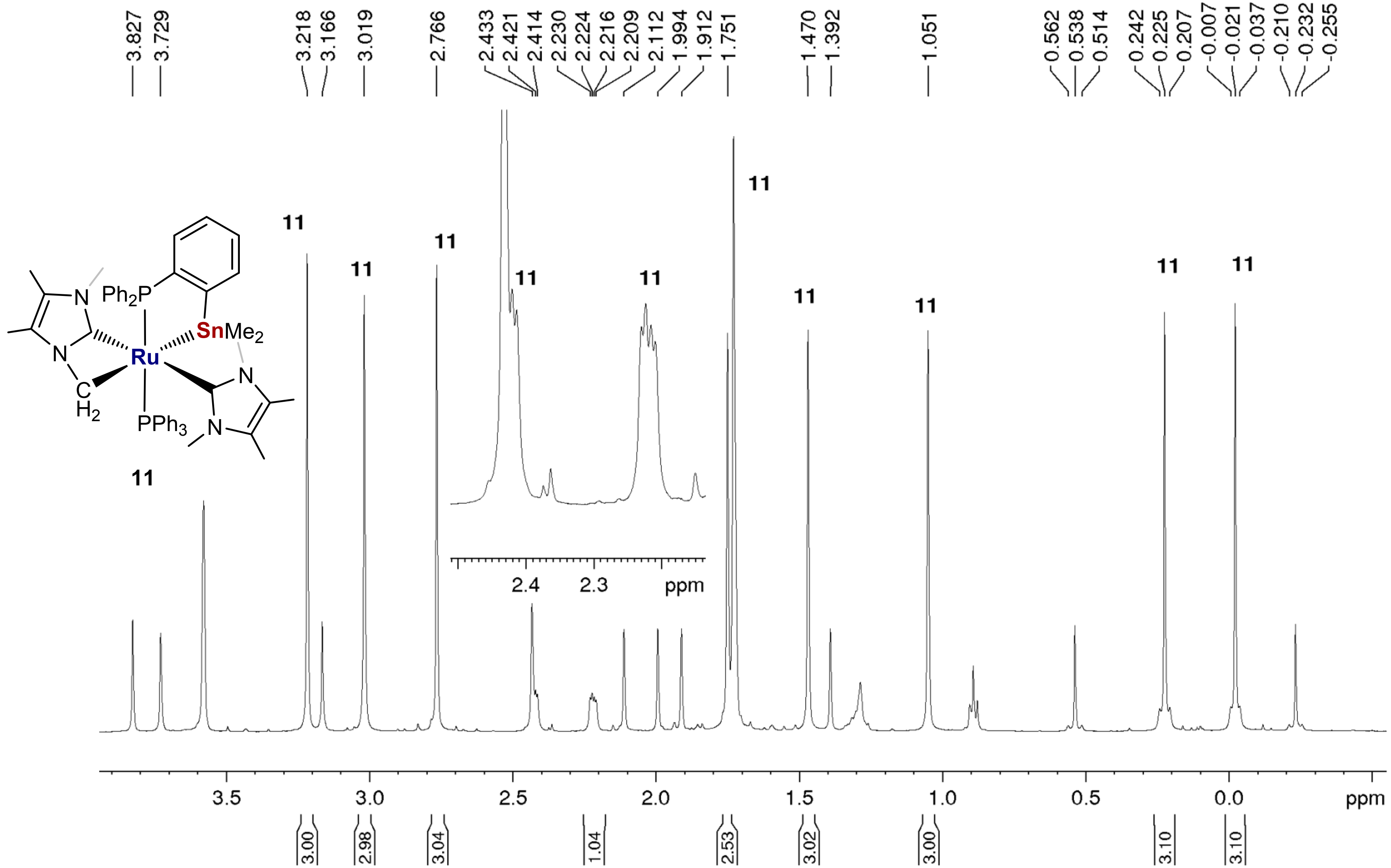

Figure S49. ^{1}H NMR spectrum (500 MHz, THF-d_8, 298 K) of precipitate isolated from thermolysis (2 h, 60 ºC) of $[Ru(IMe_4)_2(C_6H_4PPh_2)(PPh_2C_6H_4SnMe_2)]$ **10**, showing methyl resonances of $[Ru(IMe_4)(PPh_3)(IMe_4')(PPh_2C_6H_4SnMe_2)]$ **11**. Inset shows inequivalent CH_2 protons of IMe_4'.

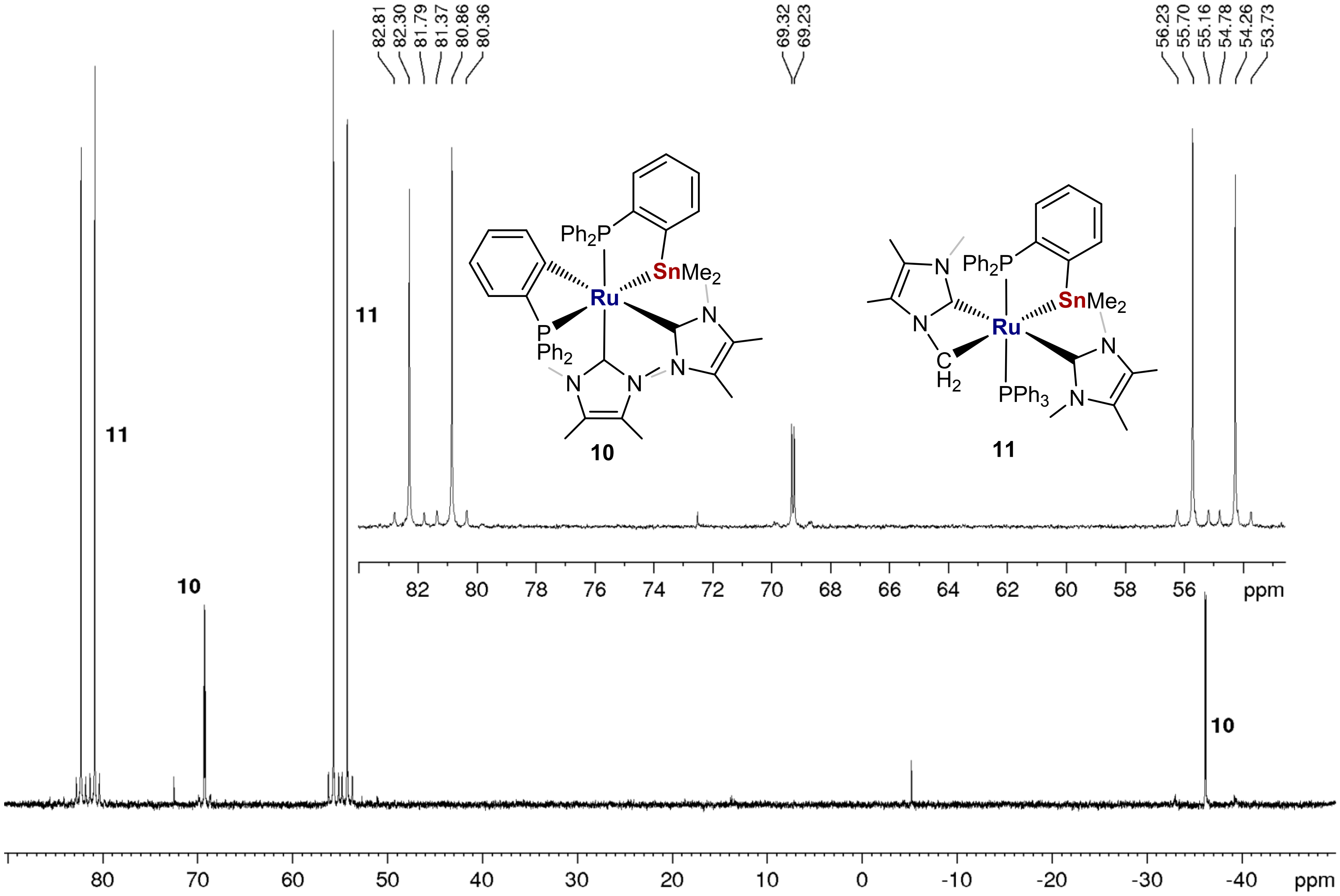

Figure S50. $^{31}P\{^1H\}$ NMR spectrum (202 MHz, THF-d_8, 298 K) precipitate isolated following thermolysis (2 h, 60 ºC) of [Ru(IMe-$_4$)$_2$($C_6H_4PPh_2$)($PPh_2C_6H_4SnMe_2$)] **10**. Inset shows an expansion of the signals for [Ru(IMe_4)(PPh_3)(IMe_4')($PPh_2C_6H_4SnMe_2$)] **11**.

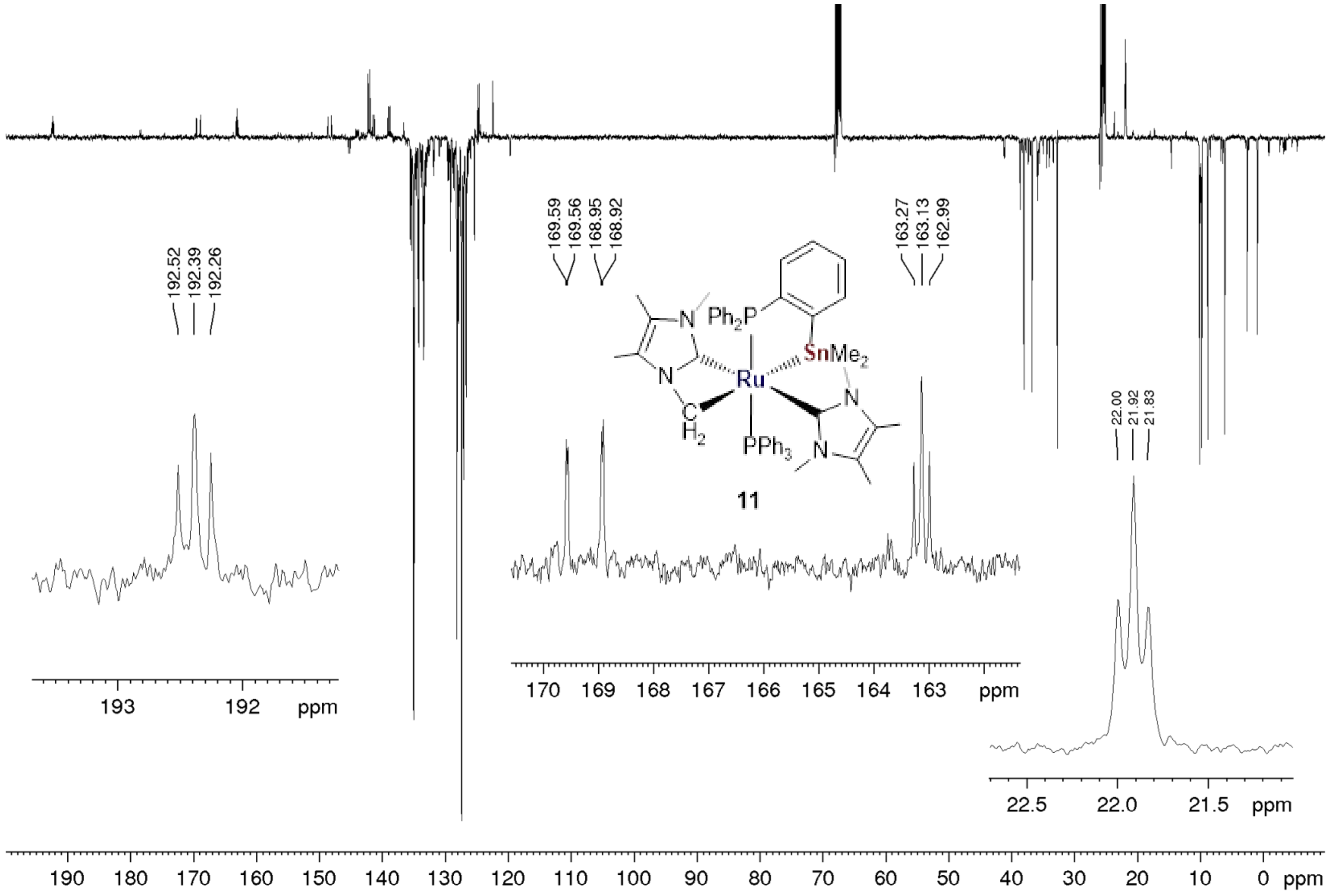

Figure S51. $^{13}C\{^{1}H\}$ DEPT-Q NMR spectrum (101 MHz, THF-d_8, 298 K) precipitate isolated following thermolysis (2 h, 60 ºC) of [Ru(IMe-$_4$)$_2$($C_6H_4PPh_2$)($PPh_2C_6H_4SnMe_2$)] **10**. The spectrum comprises mainly of signals for [Ru(IMe_4)(PPh_3)(IMe_4')($PPh_2C_6H_4SnMe_2$)] **11**; those at high frequency, and the low frequency signal of the Ru-CH_2, are shown in insets.

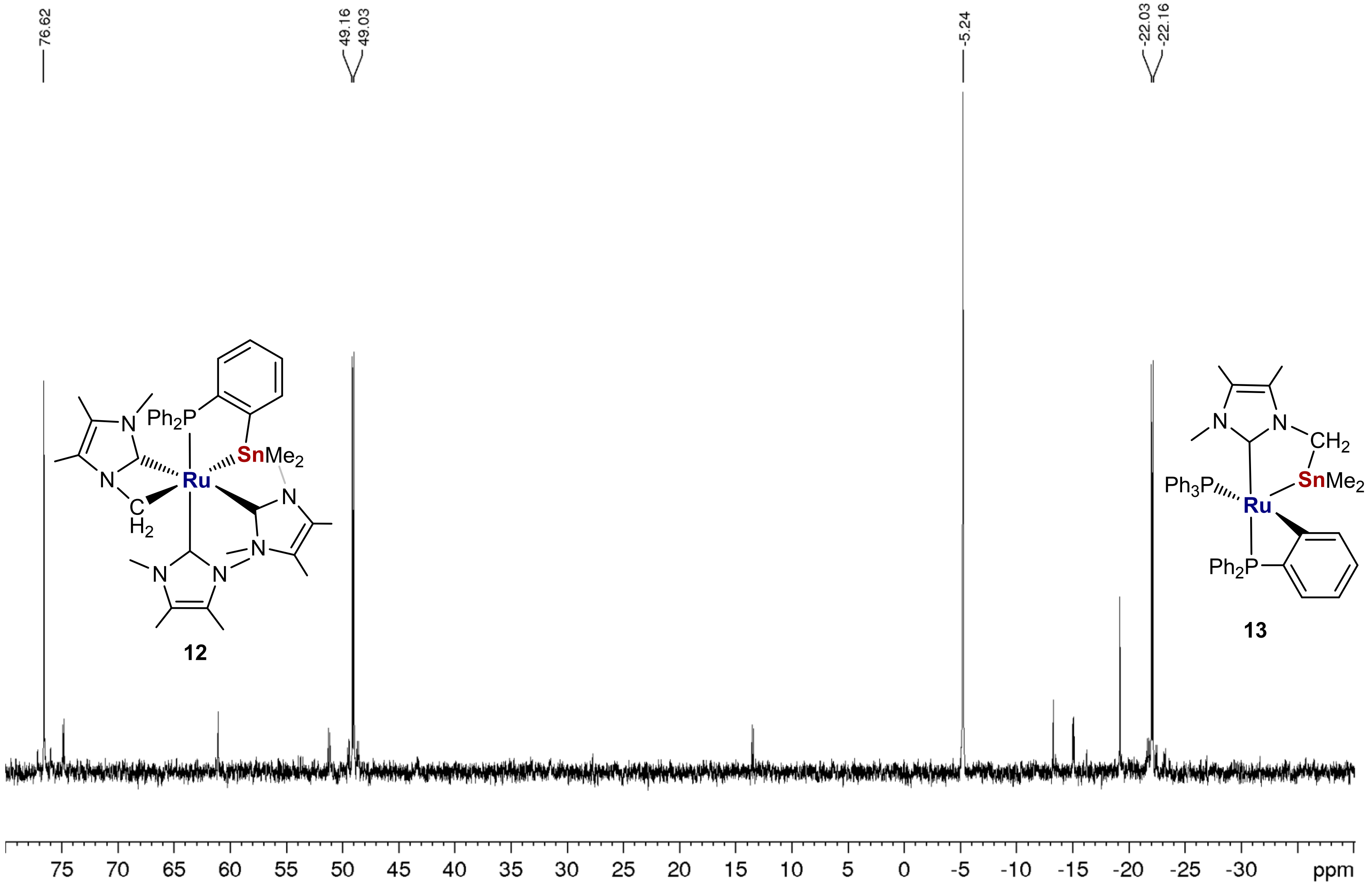

Figure S52. $^{31}P\{^{1}H\}$ NMR spectrum (162 MHz, 298 K) after thermolysis (2 h, 120 ºC) of $[Ru(IMe_4)_2(C_6H_4PPh_2)(PPh_2C_6H_4SnMe_2)]$ **10** in $C_6D_5CD_3$ showing formation of $[Ru(IMe_4)_2(IMe_4')(PPh_2C_6H_4SnMe_2)]$ **12** (δ 76) and $[Ru(PPh_3)(IMe_4'\text{-}SnMe_2)(C_6H_4PPh_2)]$ **13** (δ 49 and -22).

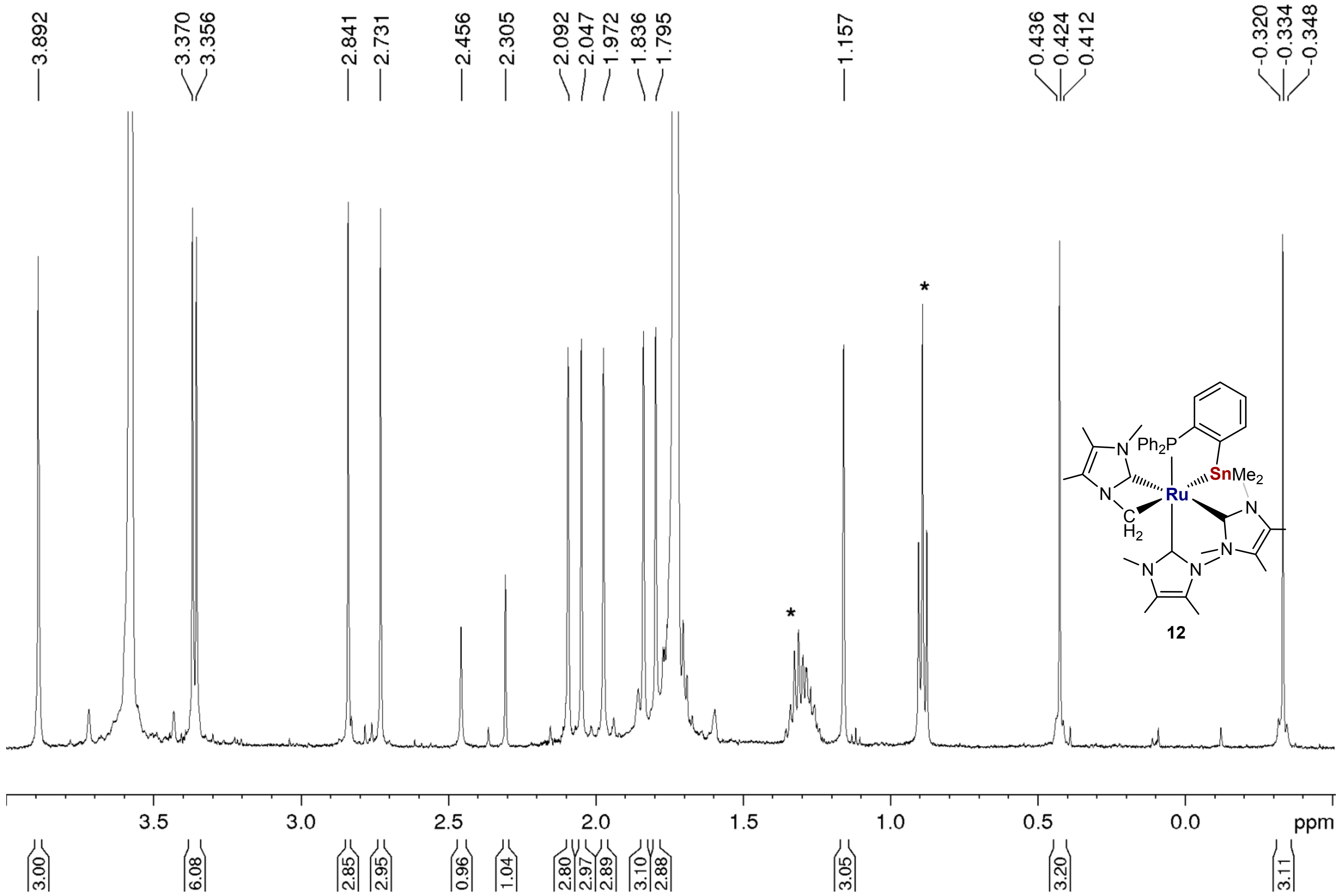

Figure S53. Methyl region of the ¹H NMR spectrum (400 MHz, THF-d_8, 298 K) of a crystal picked sample of $[Ru(IMe_4)_2(IMe_4')(PPh_2C_6H_4SnMe_2)]$ **12**. * = hexane.

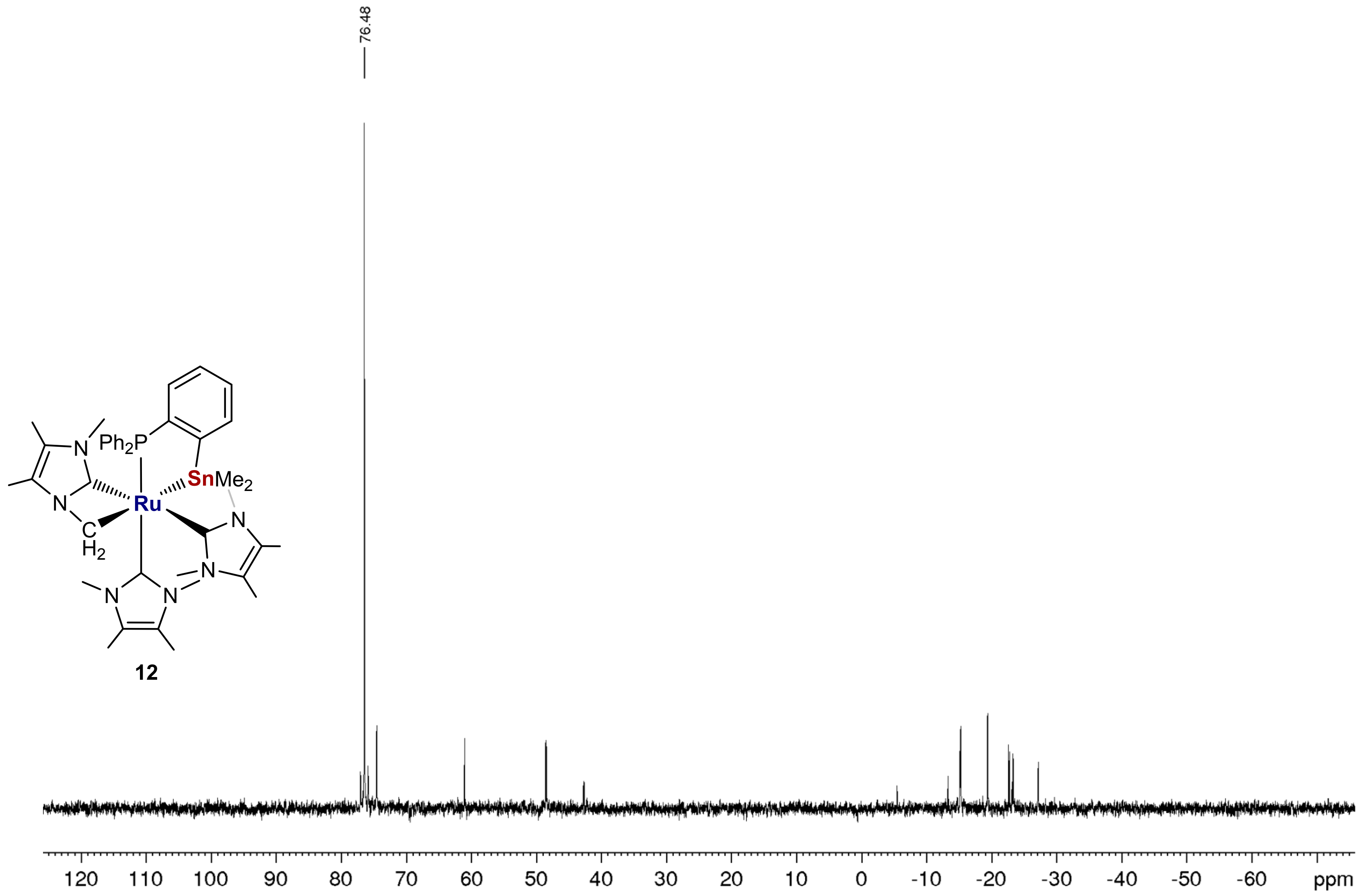

Figure S54. $^{31}P\{^{1}H\}$ NMR spectrum (162 MHz, THF-d_8, 298 K) of a crystal picked sample of $[Ru(IMe_4)_2(IMe_4')(PPh_2C_6H_4SnMe_2)]$ **12**.

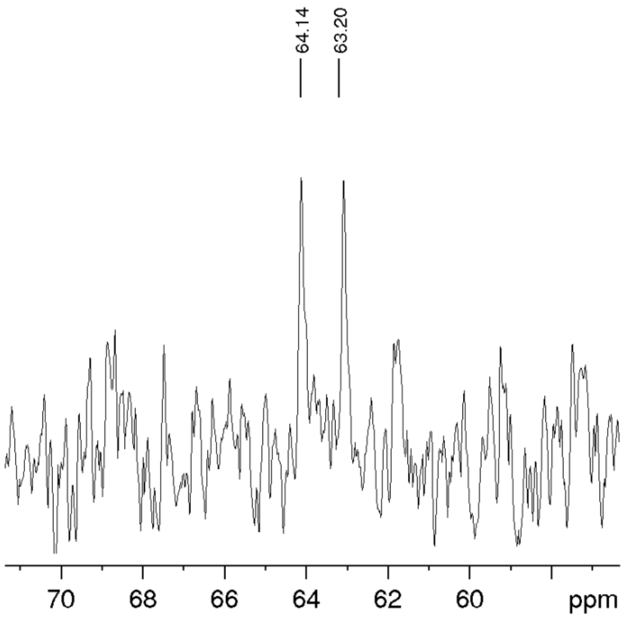

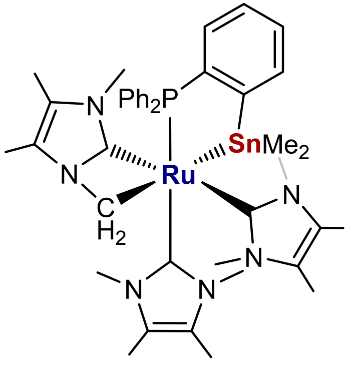

12

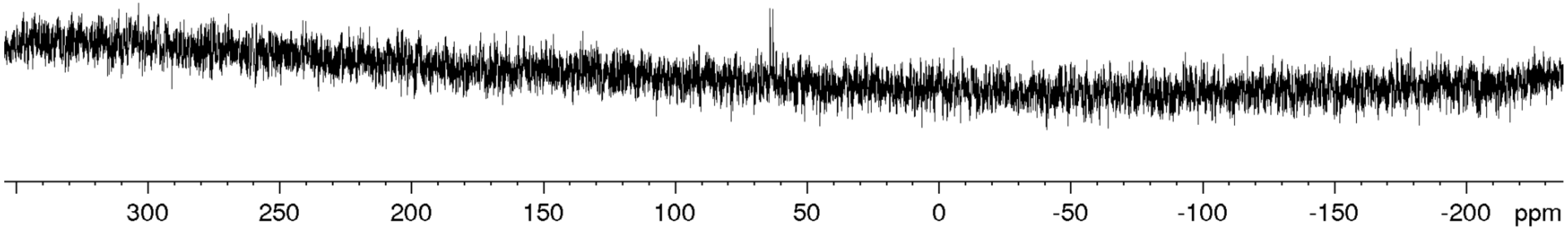

Figure S55. $^{119}Sn\{^{1}H\}$ NMR spectrum (187 MHz, THF-d_8, 298 K) of a crystal picked sample of [Ru(IMe$_4$)$_2$(IMe$_4'$)(PPh$_2$C$_6$H$_4$SnMe$_2$)] **12**.

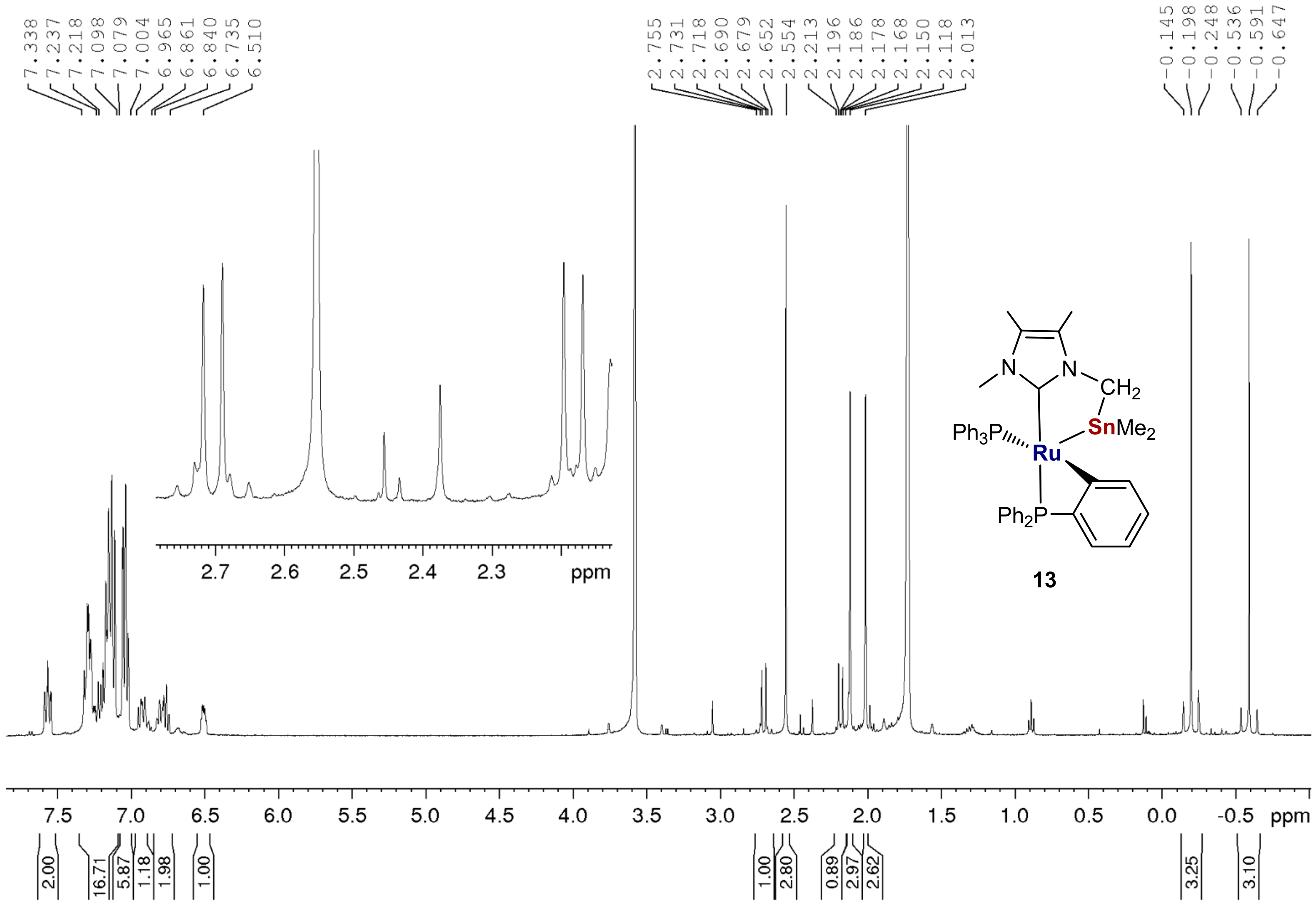

Figure S56. ^{1}H NMR spectrum (400 MHz, THF-d_8, 298 K) of [Ru(PPh_3)(IMe_4'-$SnMe_2$)($C_6H_4PPh_2$)] **13**. Inset highlights the inequivalent CH_2 protons of the IMe_4′-$SnMe_2$ ligand.

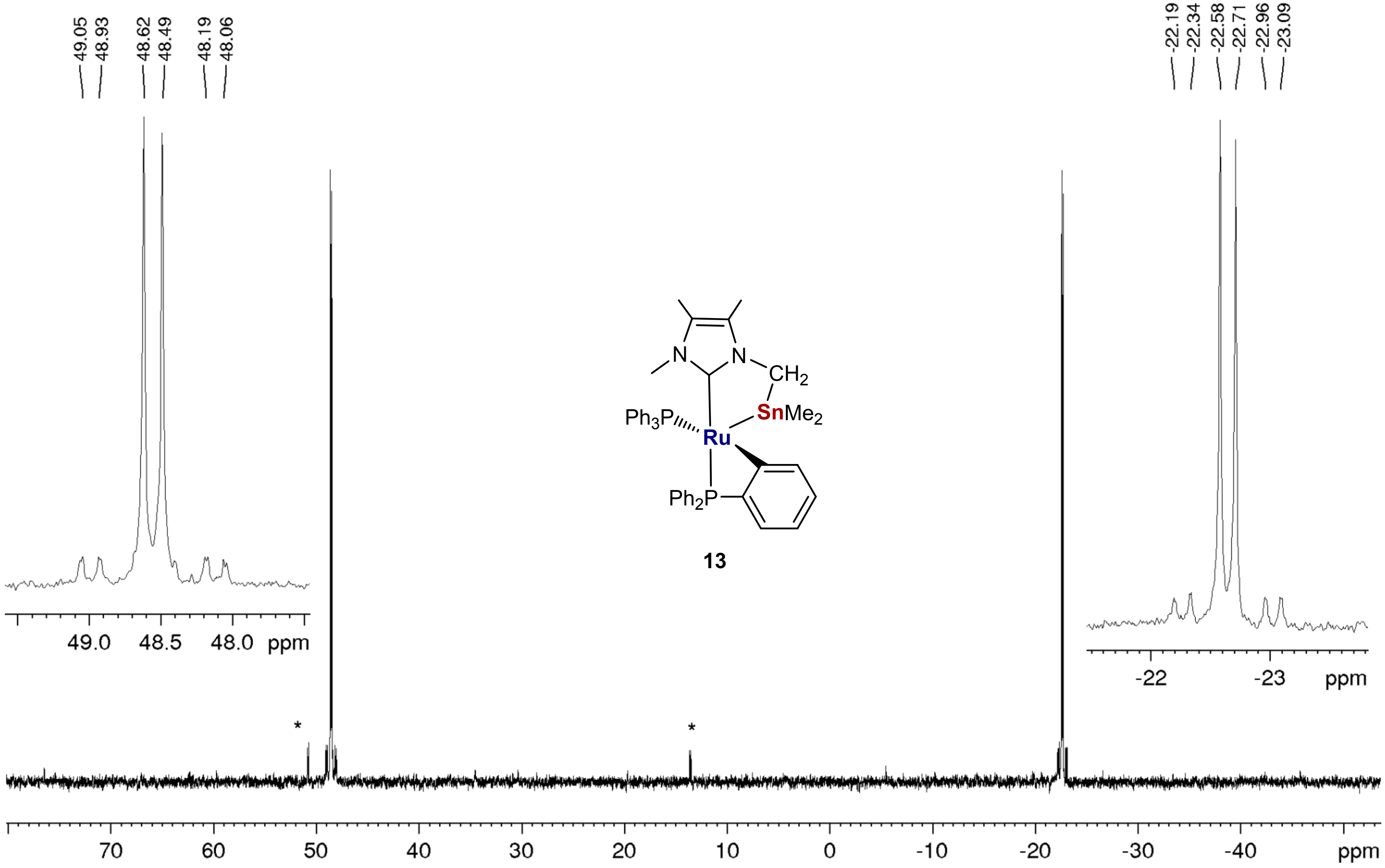

Figure S57. $^{31}P\{^{1}H\}$ NMR spectrum (162 MHz, THF-d_8, 298 K) of [Ru(PPh_3)(IMe_4'-$SnMe_2$)($C_6H_4PPh_2$)] **13**, with inset of the two resonances. The resonances marked * arise from an unknown, secondary product.

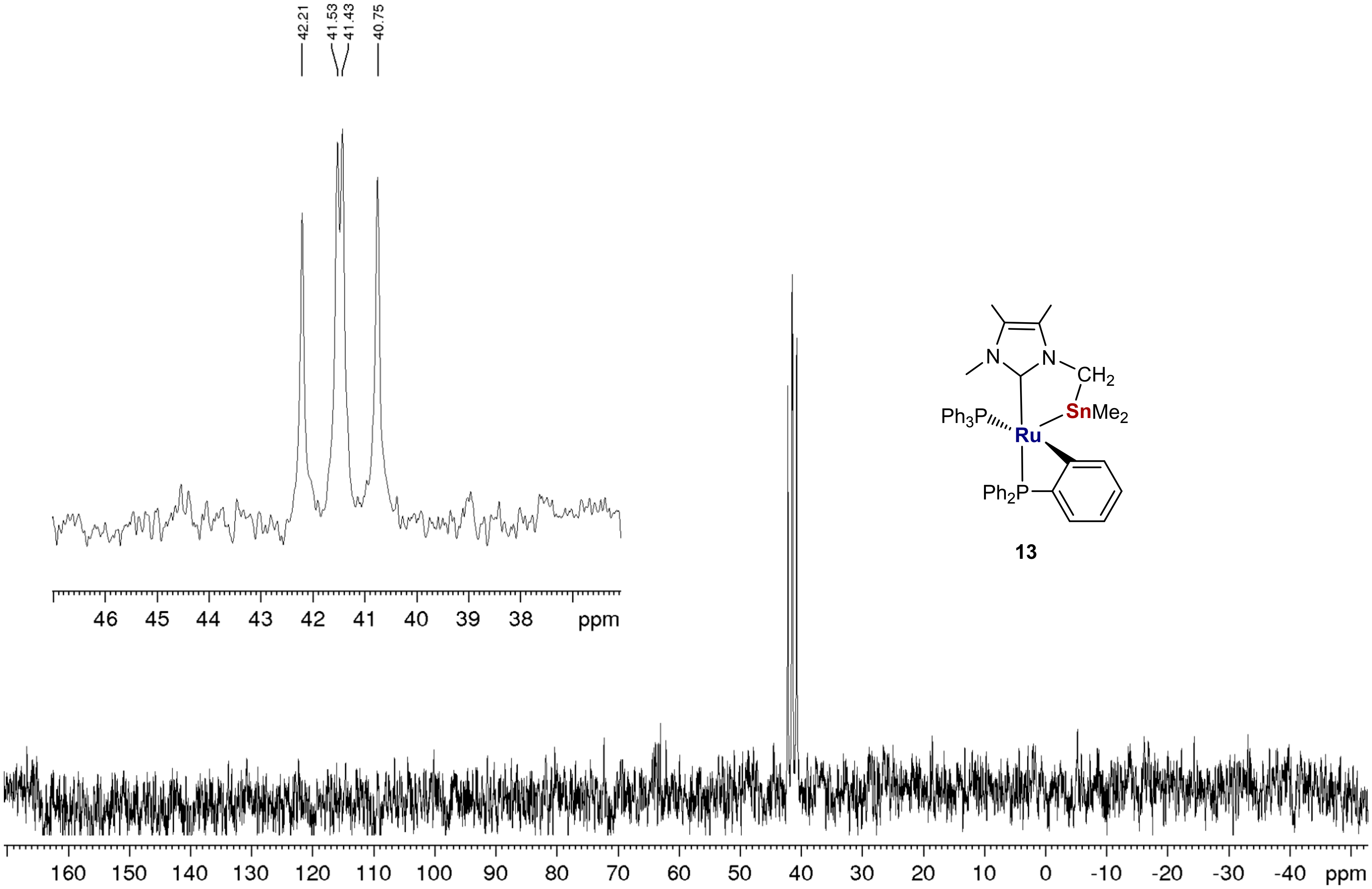

Figure S58. $^{119}Sn\{^{1}H\}$ NMR spectrum (187 MHz, THF-d_8, 298 K) of [Ru(PPh$_3$)(IMe$_4$'-SnMe$_2$)($C_6H_4PPh_2$)] **13**.

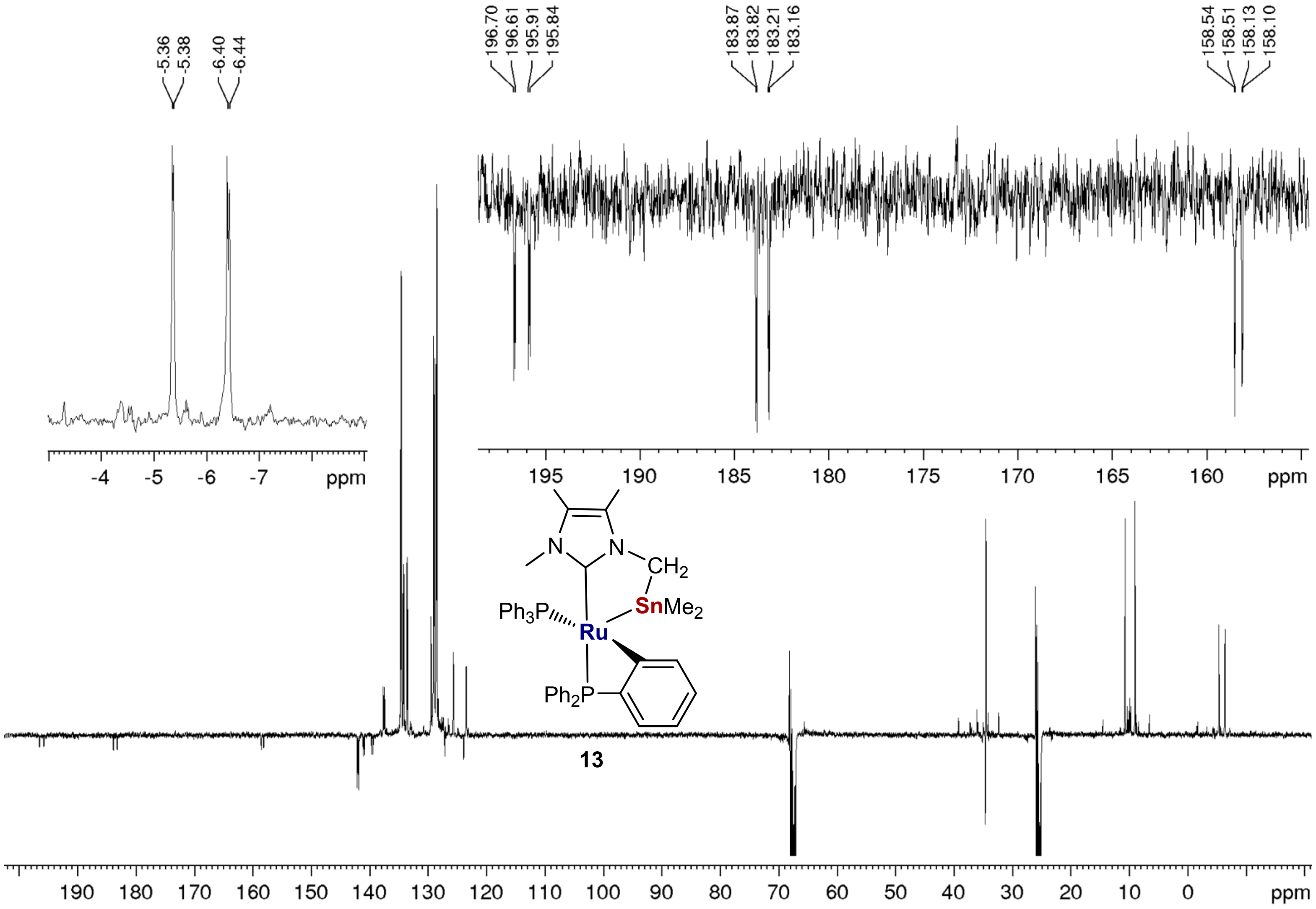

Figure S59. $^{13}C\{^{1}H\}$ DEPT-Q NMR spectrum (101 MHz, THF-d_8, 298 K) of [Ru(PPh$_3$)(IMe$_4$'-SnMe$_2$)(C$_6$H$_4$PPh$_2$)] **13** with highest and lowest frequency signals shown in insets.

Table S1. Crystal data and structural refinement details for compounds **5-6** and **9-13**.

Identification code	**5**	**6**	**9**	**10**	**11**	**12**	**13**
Empirical formula	$C_{67}H_{70}AlO_2P_3Ru$	$C_{56}H_{49}P_3RuSn$	$C_{41}H_{34}O_3P_2RuSn$	$C_{64}H_{70}N_4P_2RuSn$	$C_{52}H_{58}N_4P_2RuSn$	$C_{48}H_{63}N_6PRuSn$	$C_{45}H_{46}N_2P_2RuSn$
Formula weight	1128.19	1034.62	856.38	1176.94	1020.72	974.77	896.54
Crystal system	monoclinic	monoclinic	orthorhombic	monoclinic	monoclinic	triclinic	triclinic
Space group	$P2_1/c$	$P2_1/c$	*Pbca*	$P2_1/c$	$P2_1/n$	$P-1$	$P-1$
a/ Å	10.7869(2)	15.7864(4)	13.4185(2)	11.5411(3)	11.7722(2)	10.4377(4)	10.3666(3)
b/ Å	34.2175(6)	12.6654(2)	17.1831(2)	22.1259(4)	20.4465(3)	11.9373(4)	10.6624(4)
c/ Å	15.7686(3)	24.4671(5)	30.8438(4)	22.0265(5)	20.0653(3)	19.2141(4)	20.9764(8)
α/ °	90	90	90	90	90	77.750(2)	80.871(3)
β/ °	101.294(2)	108.426(2)	90	90.292(2)	100.565(2)	88.569(2)	86.150(3)
γ/ °	90	90	90	90	90	66.613(3)	62.494(4)
U/ Å^3	5707.49(19)	4641.18(18)	7111.70(16)	5624.6(2)	4747.85(13)	2142.63(13)	2030.40(14)
Z	4	4	8	4	4	2	2
ρ_{calc}/ g cm^{-3}	1.313	1.481	1.600	1.390	1.428	1.511	1.466
μ/ mm^{-1}	0.419	8.171	1.255	0.813	7.689	8.156	1.099
F(000)	2360.0	2096.0	3424.0	2424.0	2088.0	1004.0	908.0
Crystal size/ mm^3	0.238 × 0.191 × 0.11	0.217 × 0.115 × 0.079	0.429 × 0.166 × 0.126	0.427 × 0.417 × 0.148	0.058 × 0.053 × 0.042	0.083 × 0.072 × 0.047	0.5 × 0.385 × 0.075
2θ range for data collection/°	6.886 to 56.56	5.902 to 146.428	5.784 to 59.574	5.826 to 60.826	6.226 to 144.154	8.274 to 146.206	5.902 to 60.386
Index ranges	$-14 \le h \le 14$, $-45 \le k \le 45$, $-20 \le l \le 20$	$-19 \le h \le 19$, $-15 \le k \le 10$, $-30 \le l \le 30$	$-18 \le h \le 18$, $-23 \le k \le 23$, $-42 \le l \le 42$	$-15 \le h \le 15$, $-29 \le k \le 30$, $-31 \le l \le 30$	$-14 \le h \le 14$, $-21 \le k \le 25$, $-21 \le l \le 24$	$-12 \le h \le 12$, $-14 \le k \le 14$, $-23 \le l \le 14$	$-14 \le h \le 13$, $-14 \le k \le 13$, $-28 \le l \le 27$
Reflections collected	57540	59904	55779	52488	32059	24315	21816
Independent reflections, R_{int}	14019, 0.0537	9252, 0.0581	9415, 0.0383	15125, 0.0316	9232, 0.0531	8539, 0.0462	10349, 0.0287
Data/restraints/ parameters	14019/173/690	9252/0/552	9415/0/435	15125/27/689	9232/1/558	8539/0/481	10349/2/480
Goodness–of–fit on F^2	1.014	1.049	1.075	1.068	1.018	1.020	1.115
Final $R1$, $wR2$ [I>=2σ(I)]	0.0430, 0.0809	0.0293, 0.0742	0.0290, 0.0548	0.0339, 0.0590	0.0335, 0.0789	0.0320, 0.0658	0.0476, 0.0904
Final $R1$, $wR2$ [all data]	0.0686, 0.0890	0.0326, 0.0765	0.0407, 0.0583	0.0507, 0.0647	0.0412, 0.0832	0.0396, 0.0688	0.0617, 0.0960
Largest diff. peak/hole/ e Å^{-3}	0.46/−0.35	0.58/−0.70	0.52/−0.46	1.09/−0.67	1.63/−0.45	0.66/−0.62	1.46/−0.95

Computational Details

Breakdown of Energy Contributions

The following tables detail the evolution of the relative energies as the successive corrections to the initial SCF energy are included. Terms used are:

ΔE_{BS1} SCF energy computed with the BP86 functional with BS1

ΔH_{BS1} Enthalpy at 0 K with BS1

ΔG_{BS1} Free energy at 298.15 K and 1 atm with BS1

$\Delta G_{BS1/bnz}$ Free energy corrected for benzene solvent with BS1

$\Delta G_{BS1/bnz+D3}$ Free energy corrected for benzene and dispersion effects with BS1

ΔG_{bnz} Free energy corrected for basis set (BS2), dispersion effects and benzene solvent

In each case the final data used in the main article is highlighted in bold.

Table S2. Relative energies (kcal/mol) for computed structures. Data in bold are those used in the main text. All energies are quoted relative to **5** (for the M′ = Al system) or ***V*** (for the M′ = Sn system) at 0.0 kcal/mol. Note that **8′** is an alternative isomer of **8** shown below, the energy of which was also computed for comparison.

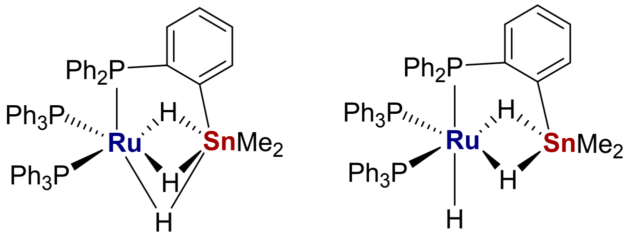

	ΔE_{BS1}	ΔH_{BS1}	ΔG_{BS1}	$\Delta G_{BS1/bnz}$	$\Delta G_{BS1/bnz+D3}$	ΔE_{BS2}	ΔG_{bnz}
**5**	0.0	0.0	0.0	0.0	0.0	0.0	**0.0**
**5 (no THF)**	6.4	5.0	-7.7	6.0	-8.1	3.4	**4.5**
IV	7.7	8.4	-6.6	7.7	-6.6	5.1	**6.0**
***IV* (+THF)**	-0.8	1.8	2.5	-0.2	3.0	-0.4	**-1.6**
**7**	-46.7	-34.6	-21.8	-46.1	-21.2	-45.9	**-20.0**
V	0.0	0.0	0.0	0.0	0.0	0.0	**0.0**
**6**	-17.2	-19.0	-29.3	-17.3	-29.4	-17.7	**-20.6**
**5Sn**	-9.9	-14.1	-23.6	-10.2	-23.9	-10.9	**-12.8**
**8**	-37.1	-27.4	-13.1	-36.7	-12.7	-34.3	**-9.3**
**8′**	-35.2	-25.8	-12.8	-35.0	-12.5	-33.7	**-6.6**

Table S3. Wiberg Bond Index (WBI) values (computed with NBO3.1) for BP86/BS1 optimized geometries at the BP86/BS2 level of theory.

	**Ru-M' Wiberg Bond Index (WBI)**
**5**	0.2531
**5**-THF	0.2742
IV	0.5164
IV +THF	0.4798
V	0.7102
**6**	0.7236
**5Sn**	0.4713

WBI Discussion. ChemDraw representations of species ***IV***, ***IV***+THF, V and **6** all depict an interaction between Ru and the equivalent M atom. This is validated by the Wiberg Bond Indices (WBI) given in Table S3, which show significantly larger covalent interactions than in **5**, **5**-THF and **5Sn** (where a hydride bridges the Ru···M' distance). In particular, for M' = Sn, these covalent interactions are significantly larger for ***V*** and **6**, in comparison to ***IV***, likely due to the larger size of Sn compared to Al. This is further evidenced by the lower WBI values for **5** in contrast to **5Sn**.

Molecular orbital analysis. MOs are orientated in Figure S60 "above" the Ru–M' axis (in the top axial position as ChemDraw structures have been drawn) to best display any orbital interactions along the Ru-M' axis . All carbon-bound hydrogens are hidden, leaving the hydride ligand visible when present. HOMOs and LUMOs do not show clear bonding interactions between Ru and M'; in some instances antibonding or orbitals on the Ru are depicted. Therefore, lower molecular orbitals were investigated to locate orbitals that showed overlap between the Ru and M' atoms. For those species containing a hydride between Ru and M', which also have lower WBI values (**5**, **5**-THF and **5Sn**), HOMO-1 shows Ru orbital

lobes along the Ru-M' axis with little/no matching M' lobes. For the other four computed species, which exhibit a Ru-M' interaction and a larger WBI value (i.e. ***IV***, ***IV***+THF, ***V*** and **6**), HOMO-3 is the MO that first shows orbital lobe overlap between Ru and M', with additional interactions also observed in HOMO-4 for M' = Sn.

<table>
<tr><td>5</td><td></td><td></td><td></td></tr>
<tr><td>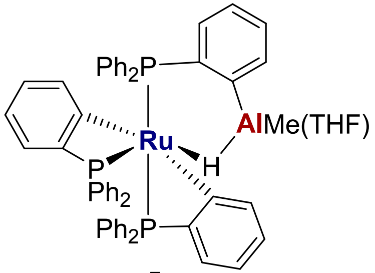

5
0.0</td><td>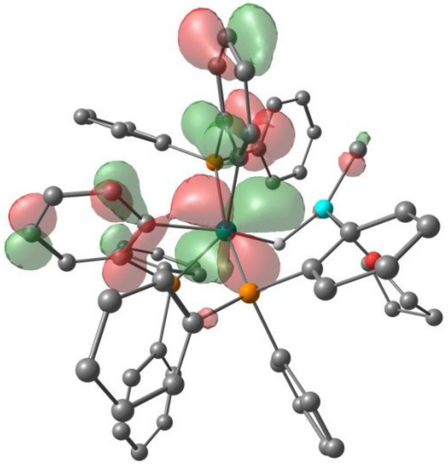
HOMO</td><td>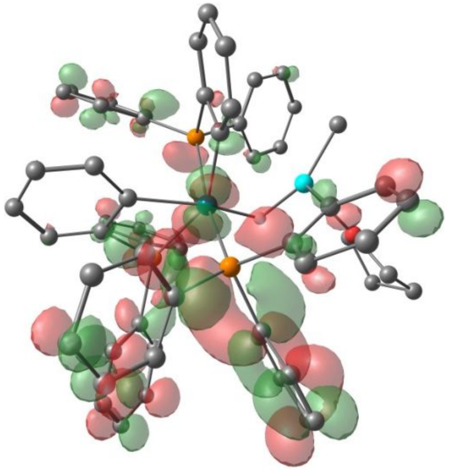
LUMO</td><td>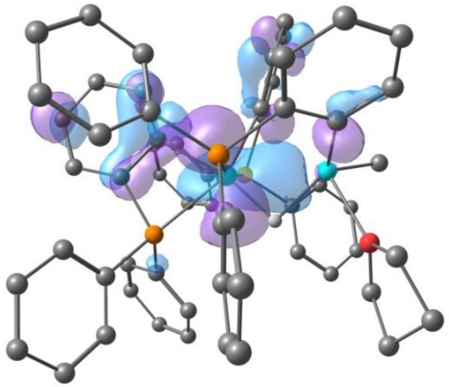
HOMO-1</td></tr>
<tr><td>5-THF</td><td></td><td></td><td></td></tr>
<tr><td>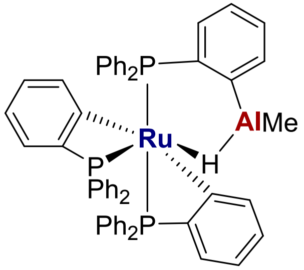

+4.5</td><td>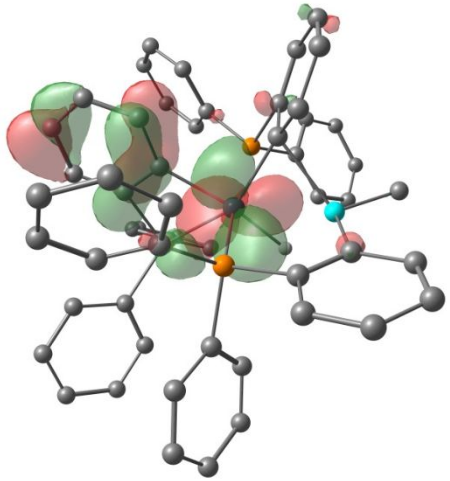
HOMO</td><td>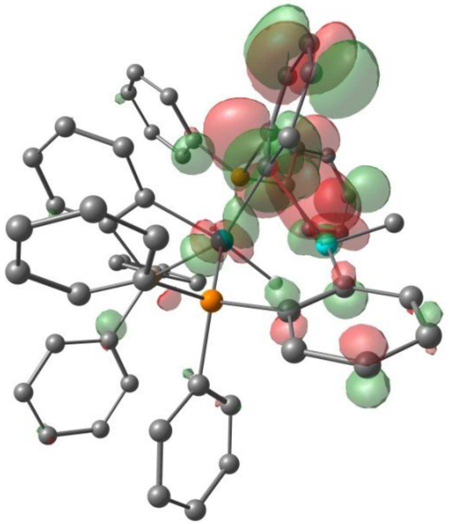
LUMO</td><td>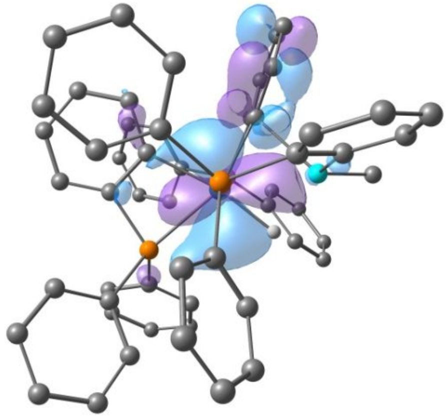
HOMO-1</td></tr>
<tr><td></td><td></td><td></td><td></td></tr>
</table>

IV			
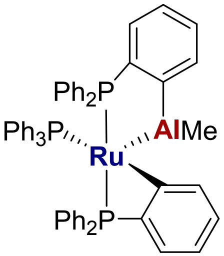 ***IV*** **+6.0**	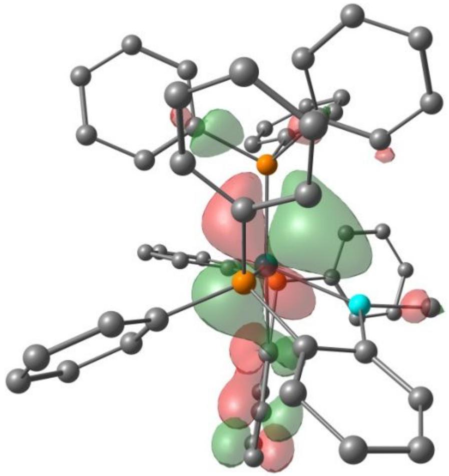 HOMO	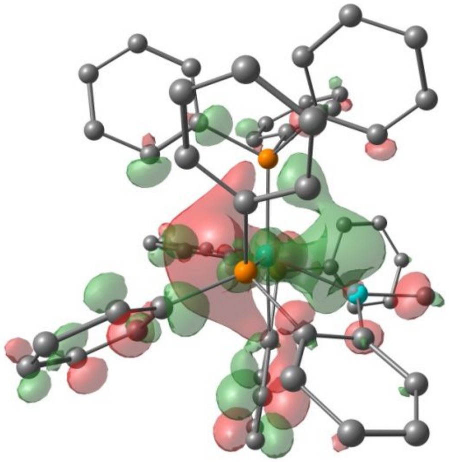 LUMO	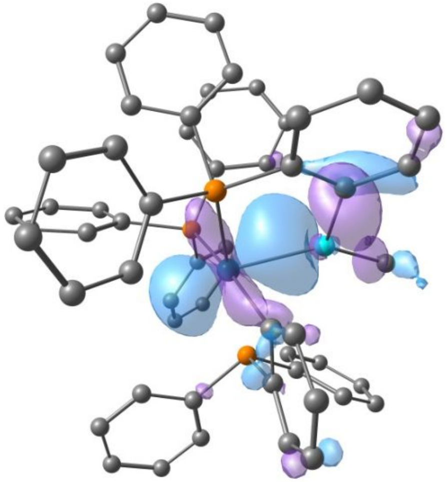 HOMO-3
IV+THF			
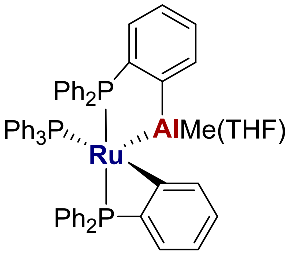 **-1.6**	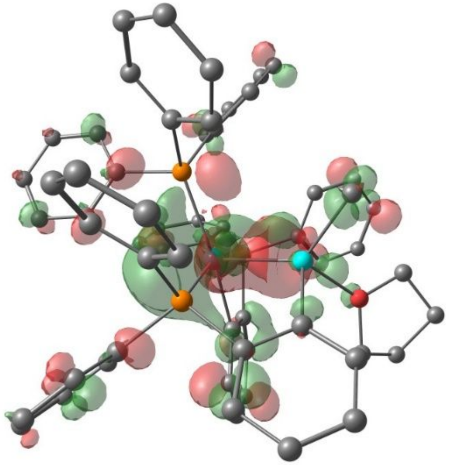 HOMO	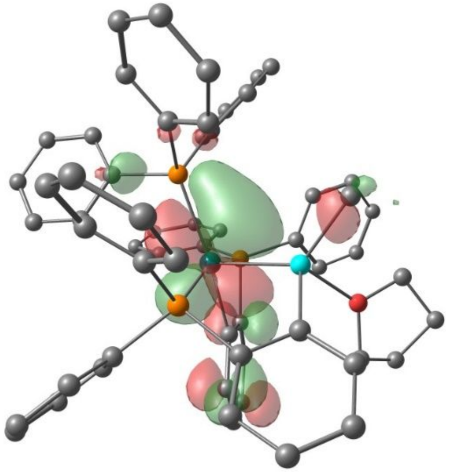 LUMO	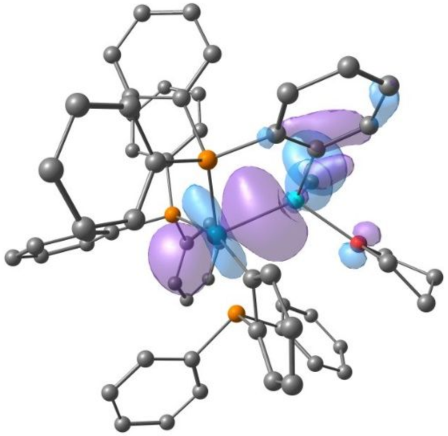 HOMO-3

<table>
<tr><th>V</th><th></th><th></th><th></th></tr>
<tr>
<td>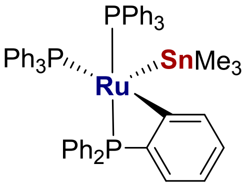

V
0.0</td>
<td>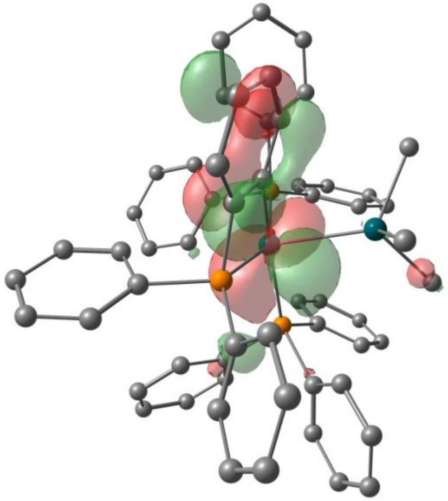
HOMO</td>
<td>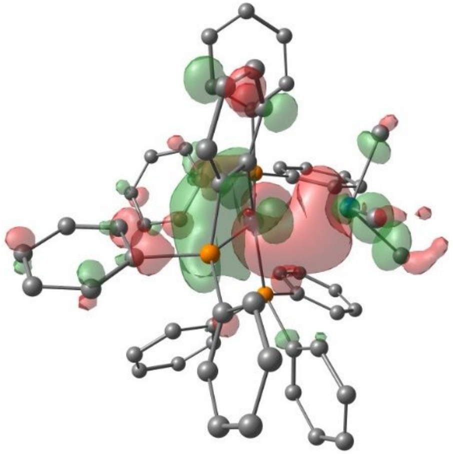
LUMO</td>
<td>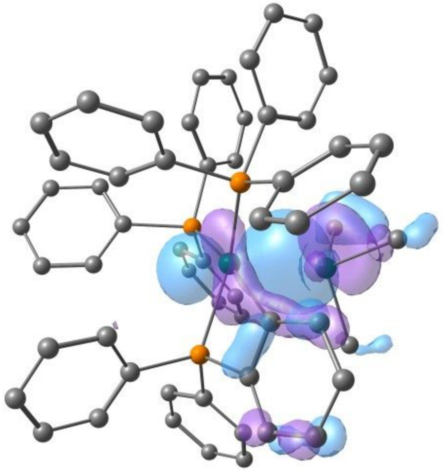
HOMO-3
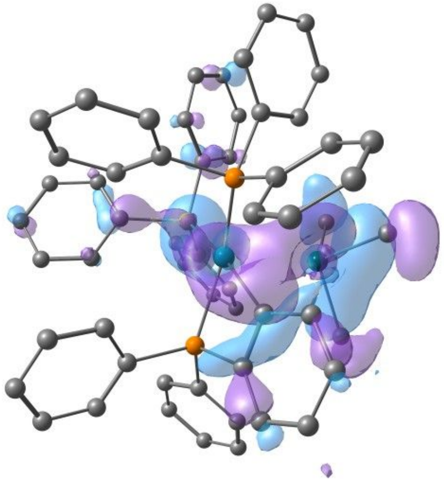
HOMO-4</td>
</tr>
</table>

6			
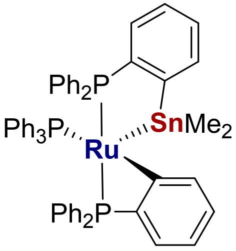<br>**6**<br>**-20.6**	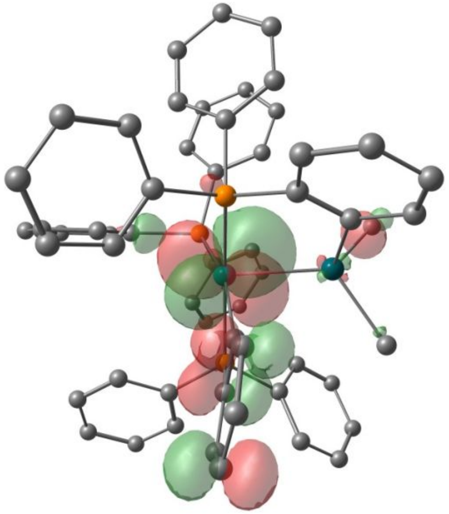<br>HOMO	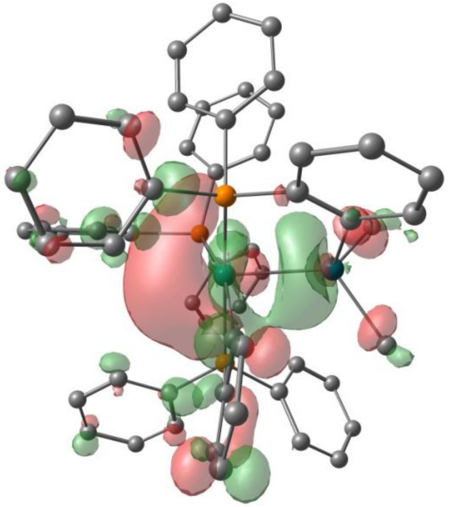<br>LUMO	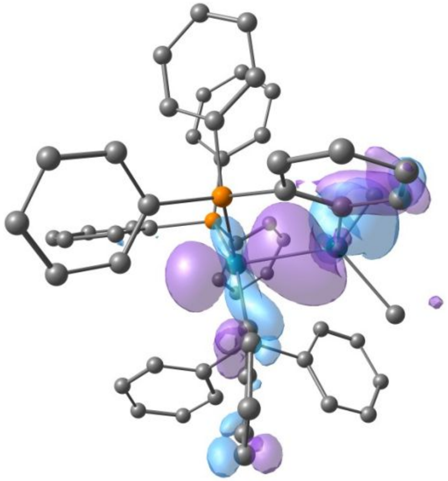<br>HOMO-3<br>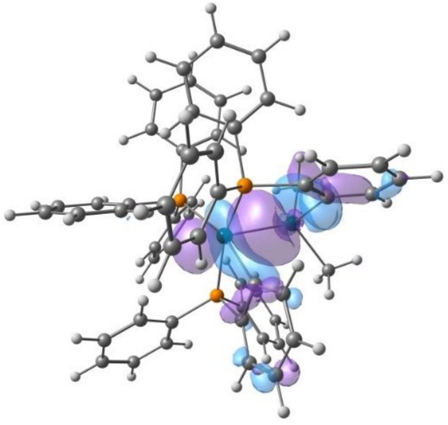<br>HOMO-4

5Sn			
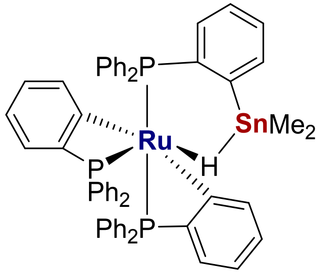 **5-Sn** **-12.8**	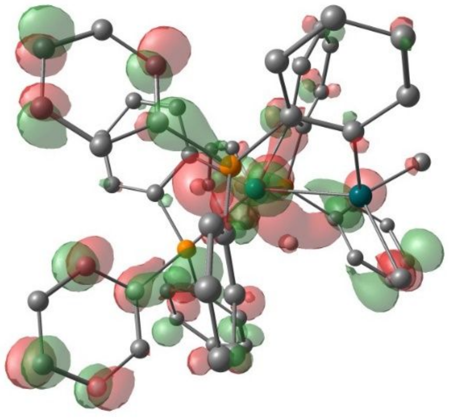 HOMO	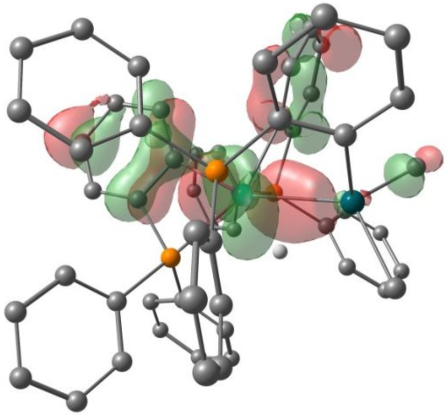 LUMO	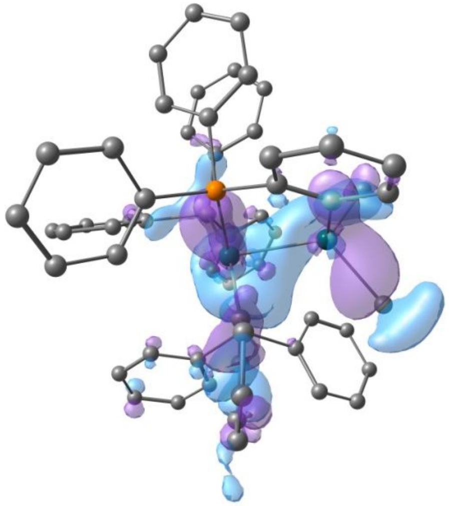 HOMO-1

Figure S60. Molecular orbitals for species **5**, **5**-THF***,*** ***IV***, ***IV***+THF, **V**, **6** and **5Sn**.

Computed Energies (in Hartrees) for Calculated Structures

CH_4
SCF (BP86) Energy = -40.5161891552
Enthalpy 0K = -40.472307
Enthalpy 298K = -40.468489
Free Energy 298K = -40.491996
Lowest Frequency = 1306.0683 cm^{-1}
Second Frequency = 1306.0745 cm^{-1}
SCF (BP86-D3BJ) Energy = -40.5177375140
SCF (C6H6) Energy = -40.5162814709
SCF (BS2) Energy = -40.5270312324

THF
SCF (BP86) Energy = -232.447574761
Enthalpy 0K = -232.333967
Enthalpy 298K = -232.327978
Free Energy 298K = -232.363078
Lowest Frequency = 23.1459 cm^{-1}
Second Frequency = 275.4150 cm^{-1}
SCF (BP86-D3BJ) Energy = -232.461052284
SCF (C6H6) Energy = -232.448881463
SCF (BS2) Energy = -232.511411715

H_2
SCF (BP86) Energy = -1.17646513415
Enthalpy 0K = -1.166541
Enthalpy 298K = -1.163236
Free Energy 298K = -1.178048
Lowest Frequency = 4356.2789 cm^{-1}
SCF (BP86-D3BJ) Energy = -1.17657513782
SCF (C6H6) Energy = -1.17652522742
SCF (BS2) Energy = -1.17751822060

PPh_3
SCF (BP86) Energy = -701.502630433
Enthalpy 0K = -701.237092
Enthalpy 298K = -701.219657
Free Energy 298K = -701.284017
Lowest Frequency = 23.6163 cm^{-1}
Second Frequency = 24.5324 cm^{-1}
SCF (BP86-D3BJ) Energy = -701.583034588
SCF (C6H6) Energy = -701.505223899
SCF (BS2) Energy = -1036.49654774

5
SCF (BP86) Energy = -2472.83842845
Enthalpy 0K = -2471.911618
Enthalpy 298K = -2471.847444
Free Energy 298K = -2472.014386
Lowest Frequency = 16.2002 cm^{-1}
Second Frequency = 16.9930 cm^{-1}
SCF (BP86-D3BJ) Energy = -2473.25901800
SCF (C6H6) Energy = -2472.84582575
SCF (BS2) Energy = -3718.01799197

5 *(without THF)*
SCF (BP86) Energy = -2240.38073462
Enthalpy 0K = -2239.569622
Enthalpy 298K = -2239.511987
Free Energy 298K = -2239.663632
Lowest Frequency = 14.9154 cm^{-1}
Second Frequency = 16.1979 cm^{-1}
SCF (BP86-D3BJ) Energy = -2240.76312427
SCF (C6H6) Energy = -2240.38733254
SCF (BS2) Energy = -3485.50117059

IV
SCF (BP86) Energy = -2240.37865538
Enthalpy 0K = -2239.564269
Enthalpy 298K = -2239.505831
Free Energy 298K = -2239.661877
Lowest Frequency = 10.1313 cm^{-1}
Second Frequency = 13.8267 cm^{-1}
SCF (BP86-D3BJ) Energy = -2240.76158424
SCF (C6H6) Energy = -2240.38475975
SCF (BS2) Energy = -3485.49848785

IV *(with THF)*
SCF (BP86) Energy = -2472.83966882
Enthalpy 0K = -2471.908738
Enthalpy 298K = -2471.844588
Free Energy 298K = -2472.010426
Lowest Frequency = 13.7707 cm^{-1}
Second Frequency = 18.4659 cm^{-1}
SCF (BP86-D3BJ) Energy = -2473.26816391
SCF (C6H6) Energy = -2472.84620755
SCF (BS2) Energy = -3718.01864313

V
SCF (BP86) Energy = -2322.13526107
Enthalpy 0K = -2321.238817
Enthalpy 298K = -2321.174598
Free Energy 298K = -2321.340878
Lowest Frequency = 16.4447 cm^{-1}
Second Frequency = 18.9970 cm^{-1}
SCF (BP86-D3BJ) Energy = -2322.55869504
SCF (C6H6) Energy = -2322.14123657
SCF (BS2) Energy = -3537.85856204

5-Sn
SCF (BP86) Energy = -2281.63480476
Enthalpy 0K = -2280.788938
Enthalpy 298K = -2280.728085
Free Energy 298K = -2280.886465
Lowest Frequency = 16.6100 cm^{-1}
Second Frequency = 19.7196 cm^{-1}
SCF (BP86-D3BJ) Energy = -2282.03745998
SCF (C6H6) Energy = -2281.64115379
SCF (BS2) Energy = -3497.34882760

6
SCF (BP86) Energy = -2281.64644783
Enthalpy 0K = -2280.796748
Enthalpy 298K = -2280.735548
Free Energy 298K = -2280.895500
Lowest Frequency = 17.5381 cm^{-1}
Second Frequency = 19.4915 cm^{-1}
SCF (BP86-D3BJ) Energy = -2282.05351738
SCF (C6H6) Energy = -2281.65259515
SCF (BS2) Energy = -3497.35969703

7
SCF (BP86) Energy = -2242.80806424
Enthalpy 0K = -2241.957824
Enthalpy 298K = -2241.898755
Free Energy 298K = -2242.054447
Lowest Frequency = 14.8296 cm^{-1}
Second Frequency = 17.1774 cm^{-1}
SCF (BP86-D3BJ) Energy = -2243.19005179
SCF (C6H6) Energy = -2242.81382488
SCF (BS2) Energy = -3487.92934619

8
SCF (BP86) Energy = -2284.05848416

Enthalpy 0K = -2283.173415
Enthalpy 298K = -2283.111117
Free Energy 298K = -2283.272504
Lowest Frequency = 17.1298 cm^{-1}
Second Frequency = 19.0250 cm^{-1}
SCF (BP86-D3BJ) Energy = -2284.46481165
SCF (C6H6) Energy = -2284.06415280
SCF (BS2) Energy = -3499.76937982

8′
SCF (BP86) Energy = -2284.05553450
Enthalpy 0K = -2283.170862
Enthalpy 298K = -2283.108300
Free Energy 298K = -2283.272039
Lowest Frequency = 12.4702 cm^{-1}
Second Frequency = 17.0388 cm^{-1}
SCF (BP86-D3BJ) Energy = -2284.45573577
SCF (C6H6) Energy = -2284.06133291
SCF (BS2) Energy = -3499.76848394
